# Current State of Open Source Force Fields in Protein–Ligand Binding Affinity Predictions

**DOI:** 10.1021/acs.jcim.4c00417

**Published:** 2024-06-19

**Authors:** David F. Hahn, Vytautas Gapsys, Bert L. de Groot, David L. Mobley, Gary Tresadern

**Affiliations:** † Computational Chemistry, Janssen Research & Development, Turnhoutseweg 30, Beerse 2340, Belgium; ‡ Computational Biomolecular Dynamics Group, Max Planck Institute for Multidisciplinary Sciences, Am Fassberg 11, Göttingen 37077, Germany; § Department of Chemistry, University of California, Irvine, California 92697, United States; ∥ Department of Pharmaceutical Sciences, University of California, Irvine, California 92697, United States

## Abstract

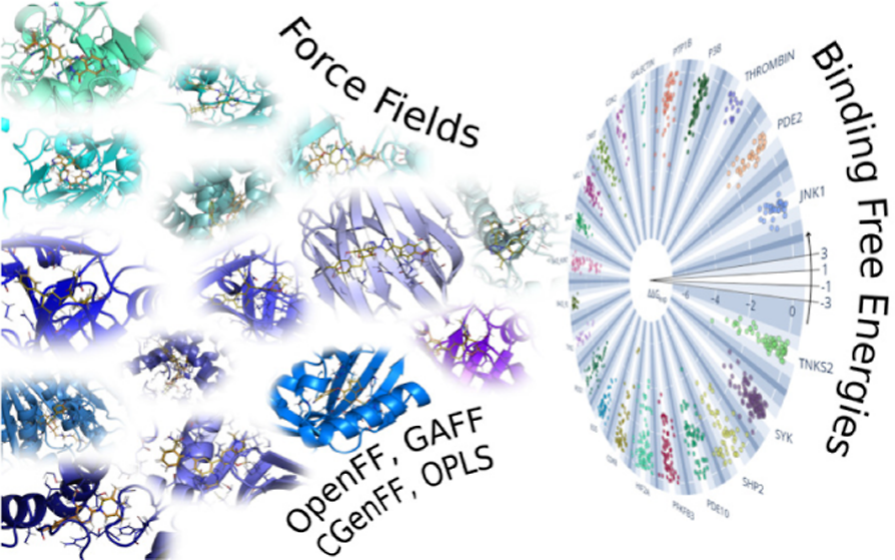

In drug discovery, the in silico prediction of binding affinity is one of the major means to prioritize compounds for synthesis. Alchemical relative binding free energy (RBFE) calculations based on molecular dynamics (MD) simulations are nowadays a popular approach for the accurate affinity ranking of compounds. MD simulations rely on empirical force field parameters, which strongly influence the accuracy of the predicted affinities. Here, we evaluate the ability of six different small-molecule force fields to predict experimental protein–ligand binding affinities in RBFE calculations on a set of 598 ligands and 22 protein targets. The public force fields OpenFF Parsley and Sage, GAFF, and CGenFF show comparable accuracy, while OPLS3e is significantly more accurate. However, a consensus approach using Sage, GAFF, and CGenFF leads to accuracy comparable to OPLS3e. While Parsley and Sage are performing comparably based on aggregated statistics across the whole dataset, there are differences in terms of outliers. Analysis of the force field reveals that improved parameters lead to significant improvement in the accuracy of affinity predictions on subsets of the dataset involving those parameters. Lower accuracy can not only be attributed to the force field parameters but is also dependent on input preparation and sampling convergence of the calculations. Especially large perturbations and nonconverged simulations lead to less accurate predictions. The input structures, Gromacs force field files, as well as the analysis Python notebooks are available on GitHub.

## Introduction

Prioritizing the synthesis of compounds by means of computationally predicted binding affinities among equally important absorption, distribution, metabolism, excretion, and toxicity properties has become one of the central strategies in small-molecule drug discovery.^1^ There are different methods, ranging from data-driven artificial intelligence to more rigorous physics-based models. Among the latter, the calculation of relative binding free energies (RBFE) from alchemical molecular dynamics (MD) simulations is probably the most frequently used and accurate method, given the accessible time scales for the size of the ligand–protein complexes. RBFE calculations involve alchemical perturbations, where a ligand is changed into another via a chemically unrealistic pathway. This can only be achieved in silico, such as by changing the atoms of one element into those of another. Following the alchemical pathways across the thermodynamic cycle will result in the same double free energy difference for the perturbation in solvent and protein as when traversing the physical pathways, i.e., monitoring the unbinding of one ligand and the binding of another. However, the alchemical transitions offer a clear sampling advantage over the physical ligand binding/unbinding pathway, thus reducing the computational cost of free energy calculations. In addition, RBFE calculations benefit from the cancellation of errors arising from calculating the separate solvation and protein legs for similar ligands.^2^ The final result of the calculation is the relative affinity of the ligand to a protein with respect to the other ligand. The reader is referred to a recent review of alchemical methods and recommendations for their use.^3^

Due to tremendous algorithmic advances, the development of user-friendly software, and the continuous increase in accuracy and computational power in the last decades, these calculations are nowadays frequently utilized. However, the calculations are still costly (compute costs of approximately 10 US$ per relative free energy difference^4^ and, in addition, potential software licensing costs). The accuracy with respect to experimental affinities is typically in the range of 1–2 kcal mol^–1^ with the best performing cases arguably capable of approaching experimental accuracy.^5−11^ When comparing to experiments, there are mainly four sources of error encountered in binding free energy calculations: system setup, force field (FF) parameters, sampling time, and experimental uncertainty. First, the setup of the system has a significant impact on the prediction accuracy. This includes the exact chemical composition of the system, consisting of proteins, ligands, solvents, potential ions, and cofactors.^12^ All the molecules need to be in their relevant tautomeric and charge states. Also, the initial coordinates of all atoms will strongly affect the results, as well as the simulation parameters mimicking the experimental conditions.^13,14^ Here, careful preparation and well-considered parameters keep this error contribution low, but this typically involves extensive manual work. The potential pitfalls and best practices to circumventing errors in system preparation were recently summarized.^15^ Furthermore, there are many approximations required to model such systems, which include the number of degrees of freedom treated, the treatment of finite-size effects, and especially the FF parameters used in classical mechanic simulations.

Another source of error in free energy estimates comes from finite sampling. Current computational power allows reaching microsecond simulation time scales, yet in large scale free energy scans, shorter sampling (up to tens of nanoseconds) is often employed. Depending on the system, such short sampling times may not be sufficient to converge the populations along the relevant degrees of freedom, e.g., ligand pose changes, amino acid rotamer motions, and water positions in the binding site. Therefore, the limited sampling does not always ensure a proper representation of the thermodynamic ensemble underlying the modeled system. This issue may be minimized by employing different or enhanced sampling protocols^16^ such as replica exchange^17,18^ or related replica methods,^19,20^ metadynamics/local elevation,^21,22^ and umbrella sampling or well designed sampling (MC) moves.^23^ Performing multiple-independent simulation repeats allows for more reliable phase space exploration and uncertainty estimation.^24,25^ Sampling improvements in relative free energy calculations may also arise by optimally planning the perturbations to be calculated,^26−28^ altering the alchemical pathway,^29−32^ using different atom mapping as in the separated topology approach,^33^ or using no atom mapping at all as in enveloping distribution sampling.^34−37^ To sample the water position sufficiently, enhanced water sampling protocols^38,39^ can be employed. Multiple options exist, such as explicit water perturbations,^40^ Monte Carlo moves,^23,41,42^ or grand canonical ensemble simulations.^43−45^

Finally, uncertainty in the experimental measurements for the reference data limits the achievable prediction accuracy.^11^ Typically, one compares the result of calculations to the experimentally measured bioactivity data, which itself has errors and is only an approximation or model to the ideal or true affinity. Additionally, the experimental data might be unsuitable for comparison because the experimental conditions differ from the simulation conditions (e.g., the temperature) or because the experiment did not measure the same observables (e.g., phenotypic vs functional assays). To keep this error low, one should use high-quality and well curated data for the comparison and above all appreciate the maximum expected performance given the underlying experimental error.^46^

While some analyses suggest that the sampling, FF, and experimental errors might contribute in a quantitatively similar manner,^47^ generally, the magnitude of each source of error is unknown and will likely be case-dependent. In the current work, we concentrate on quantifying FF-related errors by comparing six small molecule mechanic FFs in a benchmark of relative protein–ligand binding free energy calculations. For each FF, we obtained up to 1116 ΔΔ*G* estimates across 22 protein targets. The large and diverse set of systems allows a statistically meaningful comparison of not only distinct FF families—GAFF, CGenFF, OPLS, and OpenFF—but also different versions of OpenFF: v1.0, v1.2, and v2.0. With OpenFF presenting a novel direction in FF development,^48−50^ here, we demonstrate the ability of this FF to deliver high accuracy binding free energy predictions.

## Methods

### Dataset

The employed benchmark dataset is listed in the Supporting Information, Table S.1. A total of 22 protein targets, 598 ligands, and 1116 alchemical perturbations were considered.

In order to compare them to other calculations, we selected benchmark sets from previously published literature. Eight datasets originate from Wang et al.^5^ and contain the targets JNK1, TYK2, BACE, MCL1, CDK2, THROMBIN, PTP1B, and P38. Another eight datasets were assembled in the benchmark study of Schindler et al.^7^ Furthermore, we included protein–ligand systems that have appeared in various other free energy perturbation (FEP) studies: GALECTIN-3,^51^ PDE2,^52^ PDE10,^53^ ROS1,^54^ and two additional BACE datasets.^55−58^ To keep our results as comparable as possible to prior calculations, we used the same input coordinates of the prepared systems as were previously used in the studies of Gapsys et al.,^8^ Schindler et al.,^7^ and Pérez-Benito et al.^54^ The input structures are provided in the protein–ligand-benchmark repository, release 0.2.1.^59^

### Calculation Details

#### pmx/GROMACS Nonequilibrium Switching Approach

The prepared protein and ligand structures were parameterized using the corresponding FF parameters (see below). The remainder of the preparation and the simulation protocol followed the nonequilibrium thermodynamic integration protocol from the study of Gapsys et al.^8^ and is summarized as follows. For each perturbation, hybrid coordinates and topologies were generated from the physical end state ligand coordinates and topologies using pmx.^60^ A mapping between the atoms of two molecules was established following a predefined set of rules to ensure minimal perturbation and system stability during the simulations. The pmx method follows a sequential, dual mapping approach. In the first step, pmx identifies the maximum common substructure between the two molecules and proposes this as a basis for mapping. In the second step, pmx superimposes the molecules and suggests a mapping based on the interatomic distances. Finally, the mapping with more atoms identified for direct morphing between the ligands is selected. Additionally, pmx incorporates a number of empirical rules to ensure simulation stability, e.g., avoiding ring and bond breaking, preventing mappings that result in disconnected fragments, and disallowing mapping heavy atoms to hydrogens. The obtained mapping is used to create hybrid structures and topologies following a combination of single and dual topology approach.

The two branches of the thermodynamic cycle were prepared for simulation: ligand in water and ligand bound to the protein. The systems were placed in a dodecahedral box with a minimal distance of 1.5 nm to the box wall. The solutes were solvated with the TIP3P^61^ water, and sodium and chloride ions were added to neutralize the system and reach a 150 mM salt concentration.

The Amber99sb*ILDN^62−64^ FF was used to parameterize the proteins for the simulations with OpenFF and GAFF2.1x ligand FFs. The ion parameters for these simulations were taken from Joung and Cheatham.^61^ The Charmm36m^65^ protein FF was used in combination with the MATCH/CGenFF ligand parameters.

To calculate relative free energy differences, first, every system was simulated at equilibrium in its physical state, e.g., ligand X representing state A and ligand Y representing state B. The simulation protocol involved energy minimization, followed by a brief 10 ps *NVT* equilibration and finally a production run for 6 ns in the *NPT* ensemble, where frames were written to file every 47 ps. From the generated trajectories, the first 48 frames (2.256 ns simulation time) were discarded, and from the rest, 80 snapshots were extracted. These configurations were used to perform rapid (50 ps) alchemical transitions between the physical states: from state A to state B when starting from the equilibrium ensemble generated at the state A and vice versa. The whole procedure, starting with energy minimization and ending with the fast alchemical transitions, was repeated 3 times. Each repeat used different random initial ion coordinates and initial velocities for the *NVT* equilibration. All in all, the simulation time for one leg of the thermodynamic cycle of 3 replicas adds up to 60 ns for each double free energy difference. This is an equivalent simulation time to a classical equilibrium FEP approach using twelve 5 ns lambda windows, which happens to be the default in the commercial FEP+ software and is used in many published studies.^5^

The simulation temperature was kept at 298 K by means of the stochastic dynamics integrator with a friction of 0.5 ps^–1^.^66^ This protocol is in line with that previously described in ref (8) except that ref (8) used MD integrator in combination with the velocity rescaling thermostat^67^ with a time constant of 0.1 ps. The pressure was controlled by means of the Parrinello–Rahman barostat^68^ with a time constant of 5 ps, keeping pressure at 1 bar. Electrostatic interactions were treated by means of the particle mesh Ewald (PME) method^69,70^ with a direct space cutoff of 1.1 nm, a relative strength of interactions at a cutoff of 10^–5^, and a Fourier grid spacing of 0.12 nm. Van der Waals interactions were switched starting at 1.0 nm distance, and were completely turned off for the distances reaching 1.1 nm. Dispersion correction was used to adjust energy and pressure. Nonbonded interactions during the alchemical transitions were softened. The functional form of the softcore potential described in ref (29) (with the default set of parameters) was used for the transitions in PDE2, GALECTIN, BACE (Hunt), BACE, BACE (P2), CMET, JNK1, TYK2, MCL1, CDK2, THROMBIN, PTP1B, and P38 systems. For the alchemical transitions in the other systems, the softcore potential described in ref (71) was used with the parameters α = 0.3 and σ = 0.25 nm. The bonds were constrained by means of the LINCS algorithm.^72^

From the alchemical transitions, work values were collected, and free energy differences were calculated based on the Crooks fluctuation theorem^73^ using a maximum likelihood estimator.^74^

### Free Energy Perturbation Using FEP+

The free energy calculations using Schrodinger’s FEP+^5^ were retrieved from published results, and the calculation details can be found therein.^7,8,54^ The calculations use the same input structures as those available in the reference dataset as well as the same alchemical perturbations.^59^ The previously published FEP+ results were generated by the automated Schrodinger protocol with default settings, i.e., 5 ns simulation time, 12–24 λ points per perturbation, Hamiltonian replica exchange, and the replica exchange solute tempering protocol. The proteins and ligands were parameterized using the OPLS3e FF with custom parameters,^75^ as described in the respective publications.^7,8,54^ The results for targets BACE, BACE (HUNT), BACE (P2), CDK2, GALECTIN, JNK1, MCL1, P38, PDE2, PTP1B, THROMBIN, and TYK2 are retrieved from ref (8). Reference (7) is the source of the results for targets CDK8, CMET, EG5, HIF2A, PFKFB3, SHP2, SYK, and TNKS2. Finally, the results of targets PDE10 and ROS1 are taken from ref (54).

### Small Molecule Force Field Parameterizations

Below, we provide small molecule parameterization details. As the simulation data was collected from multiple literature sources, we summarize the particular FF version used for each system in the Supporting Information, Table S.1.

#### Open Force Field

Open Force Field (OpenFF) parameters were used in 3 different versions (Parsley v1.0.0^49^ and v1.2.1 and Sage v.2.0.0^50^). The OpenFF toolkit 0.8.4^48,76^ was used to parameterize the ligands with Austin Model 1-bond charge correction (AM1-BCC) charges.^77,78^ In the following, the three FFs are named OpenFF-1.0, OpenFF-1.2, and OpenFF-2.0, without the last patch number of the release.

#### GAFF2.1x

GAFF parameters were assigned by means of Antechamber^79^ and ACPYPE.^80^ The AM1-BCC partial charge model was used.^77,78^ Off-site charges on chlorine and bromine were added according to the rules, as described in ref (81). The effect of the off-site charges in perturbations concerning chlorine and bromine atoms is analyzed in the Supporting Information, Figure S.15. We specify the FF as “GAFF2.1x” as results across the dataset are pulled from two different studies, with some systems using GAFF2.1^8^ and a later study using GAFF2.11.^82^Table S.1 lists the exact FF used for each target.

#### CGenFF/MATCH*

Small molecule parameterization with the CGenFF^83^ was performed by assigning atom types with the MATCH^84^ tool and subsequently replacing the bonded parameters with those in CGenFF v3.0.1. For the BACE inhibitor sets, the MATCH algorithm was unable to identify the appropriate atom types; therefore, in these cases, a web-based atom-typing and parameter assignment server^85,86^ was used in combination with the CGenFF v4.1 parameters. As for GAFF2.1x above, virtual charged sites were added to chlorine and bromine containing ligands (Supporting Information, Figure S.15).^87^ Throughout the article, we refer to this parameterization as CGenFF/MATCH* to mark that several different tools were employed in the parameterization procedure, which may lead to differences in assigned parameters depending on the atom-typing, generalized FF version, and even structure converter used.^88^

#### OPLS3e

The Schrodinger FF OPLS3^89^ and OPLS3e^75^ were used in the FEP+ results presented, which were taken from published sources.^7,8,54^Table S.1 lists the source of the results for each target. For simplicity, we labeled all the FEP+ results in the plots and tables as “OPLS3e”. Note that differences in results between OPLS3e and the other FFs are not only due to the FF parameters, but may additionally originate from the different MD engine and sampling protocol.

#### Consensus Approach

For the consensus approach “Consensus”, the results were averaged over the first repeat of the simulations using OpenFF-2.0, GAFF2.1x, and CGenFF/MATCH*. This sums up to the same sampling time as the results from the single FFs.

Two alternative consensus approaches were calculated, which are presented in the Supporting Information. The first one was obtained from an average over GAFF2.1x and OpenFF2.0 (referred to as “Consensus (OFF, GAFF)”), while the second one was obtained as an average over GAFF2.1x, OpenFF2.0, CgenFF/MATCH*, and OPLS3e (referred to as “Consensus (all)”).

### Analysis

All the graphs and analyses presented in this article can be followed and reproduced with the Python notebooks available at https://github.com/dfhahn/protein-ligand-benchmark-analysis.^90^

#### Calculation of ΔΔ*G* and Δ*G* Values

For the RBFE (ΔΔ*G*) values, we used the raw values without any cycle closure correction as they reflect better potential shortcomings of FFs. For the pmx results, we calculated the ΔΔ*G* values as averages over three repeats, and the standard deviation across the repeats was used as an error estimate.

For the binding free energy estimates (Δ*G*), we calculated the maximum likelihood estimate with the package arsenic^91^ for ΔΔ*G* values coming both from FEP+ and pmx.

#### Metrics

The performance of the calculations employing different FFs is evaluated based on various error and ranking metrics. The aggregated statistics are calculated as the pairwise root mean squared error (RMSE) and mean unsigned error (MUE) of the calculated relative binding free energies (ΔΔ*G*) compared to the experimental values. These were calculated for the individual target sets and the whole set of 1116 edges.

For the final binding free energies of ligands (Δ*G*), the node-based RMSE and MUE were calculated, as well as the ranking coefficients Kendall’s τ_K_ and Spearman’s ρ. Again, we calculated the statistics for various subsets of the full dataset as well as for the whole set of 598 ligands. For the calculation of Kendall’s τ_K,overall_ considering the whole dataset, we calculated the weighted average of the Kendall’s τ_K_ of all individual targets
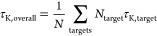
1where *N* is the sum of all considered ligands across targets, *N*_target_ is the number of ligands of a target, and τ_K,target_ is the corresponding Kendall’s τ_K_ of the target. Note that only resulting RMSE values and Kendall’s τ_K_ are discussed in the main text, but values for MUE and Spearman’s ρ can be found in the Supporting Information.

#### Error Calculation

If not stated otherwise, all results are given with a 95% confidence interval, obtained from bootstrapping using 1000 bootstrap samples. The lower and upper bounds of the interval are given as sub- and superscripts behind the actual value.

#### Significance Test

To evaluate if there is a significant difference between two calculated sets compared to the experiment, we calculated the significance by bootstrapping using a confidence interval of 95%.

#### Convergence Criteria for Perturbations

To discriminate the error of FF parameters from sampling errors, the set of all edges was filtered according to two convergence criteria indicating issues with sampling. The first criterion is the convergence criterion α based on the overlap of the work distributions from the nonequilibrium sampling. α is defined in the range −1 ≤ α ≤ 1 and is described in more detail in ref (92), eq 5. The second criterion is the standard deviation of the ΔΔ*G* values σ(ΔΔ*G*) over the three repeats. For a perturbation to be considered converged, both requirements α < 0.8 and σ(ΔΔ*G*) < 1.5 kcal mol^–1^ must be true.

#### Parameter Analysis

We performed a parameter analysis to investigate the influence of certain OpenFF parameters on the errors. For each perturbation, the FF parameters involved in the perturbations were identified, i.e., only the parameters that were either changed or annihilated during the perturbation. For each parameter, the RMSE across all perturbations involving this parameter was calculated. As parameters are often used in the same combination (e.g., the bond, angle, and torsion parameters describing an ester group), the correlation between parameters used in the same edges was calculated using the Matthew’s correlation coefficient,^93^ as it is suited to correlate binary vectors (parameters either used or not used in edges). The obtained correlation matrix between parameters was then clustered with spectral clustering^94^ to identify groups of parameters, which are used simultaneously in perturbations. To analyze the influence of a parameter change from OpenFF-1.0 to OpenFF-2.0 on the prediction error, the ΔRMSE of parameter *p* was calculated as
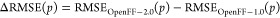
2where RMSE_FF_(*p*) is the RMSE between predicted ΔΔ*G* with FF and experimental ΔΔ*G* of all perturbations involving a perturbation of parameter *p*.

## Results and Discussion

### Prediction Accuracy

#### Overall Performance of Various Force Fields Analyzed Based on ΔΔ*G*

**Figure 1:**
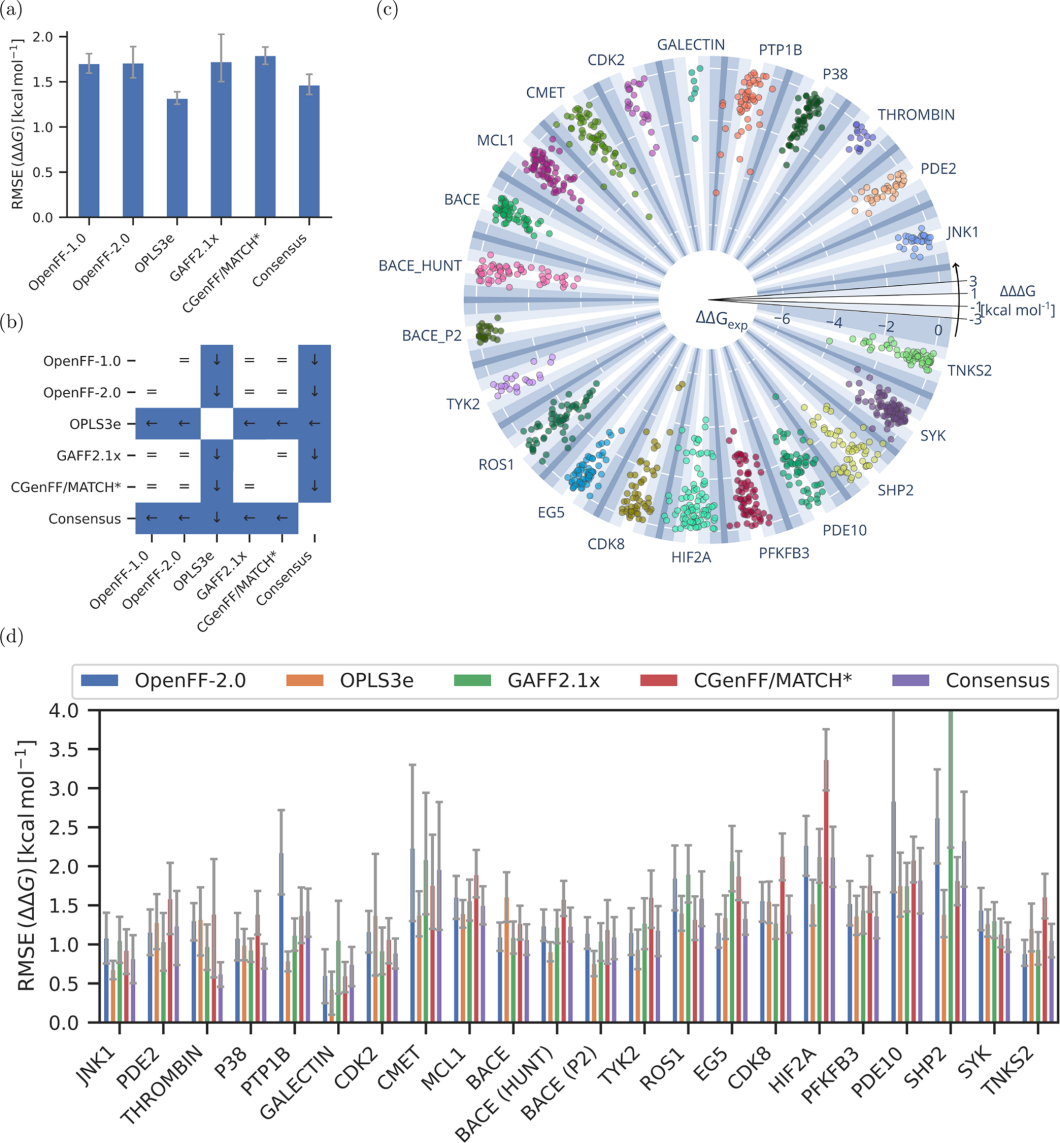
Comparison of ΔΔ*G* values of the perturbations obtained from calculations using the five force fields OpenFF-1.0, OpenFF-2.0, GAFF2.1x, CGenFF/MATCH*, and OPLS3e and the consensus approach. (a) Overall RMSE comparison across all targets and all 1116 perturbations. (b) Illustration of significant differences between pairs of force fields. White matrix element with an equal sign (“=”) means that the differences between the two force fields are statistically insignificant. Colored matrix element denotes a significant difference considering a 95% confidence interval. Arrow in a blue matrix element points at the force field, which has the lower error (either left or down). (c) Comparison of all experimental and calculated binding free energy differences for the OpenFF-2.0 Sage force field. All edges belonging to one target are shown in one color in a segment of the circle. Radial distance denotes the experimental ΔΔ*G*_exp_. Deviation of the calculation from experiment is shown on the angular axis as deviation from the segment center (white background). Scale of this deviation is illustrated in the right segment and also coded in background color. (d) RMSE values for each target separately. Each group represents a target set with the RMSE values between experimental and calculated value for the respective force fields in different colors. Lower and upper bound of the 95% confidence interval are given as error bars. Corresponding graph with MUE instead of RMSE can be found in the Supporting Information, Figure S.2.

The general summary of the benchmark study is provided in Figure 1 illustrating all performed RBFE calculations (1116 edges) for 22 targets. In Figure 1c, we used the recent OpenFF, OpenFF-2.0 (Sage), to exemplify the accuracy achievable with the open source FF. The results for each target are shown in different colors in separate segments of the circle. The radial distance denotes experimental ΔΔ*G*_exp_, showing that there are varying dynamic ranges among the targets. The deviation of the calculation from experiment ΔΔΔ*G* = ΔΔ*G*_calc_ – ΔΔ*G*_exp_ is shown on the angular axis as a deviation from the segment center (white background). Based on the ΔΔ*G* values of the edges, a RMSE of 1.7_1.6_^1.9^ kcal mol^–1^ (MUE = 1.2_1.1_^1.3^ kcal mol^–1^) was obtained. This is in line with current industry standards.^7^

Overall, the open source FFs performed comparably to one another and did not show significant differences in terms of ΔΔ*G* prediction for the results averaged over the whole set of targets and chemical series (Figure 1a,b). The obtained RMSE values from the experiment are: GAFF2.1x 1.7_1.5_^2.0^, OpenFF-1.0 1.7_1.6_^1.8^, OpenFF-2.0 1.7_1.6_^1.9^, and CGenFF/MATCH* 1.8_1.7_^1.9^ kcal mol^–1^. It is interesting to note that a consensus variant constructed as a linear combination over three open source FFs significantly outperformed each of the open source FFs considered separately (RMSE of 1.5_1.4_^1.6^). The OPLS3e FF shows a significantly lower RMSE of 1.3_1.3_^1.4^ kcal mol^–1^ when averaged over all ΔΔ*G* values calculated in this work. Note that more recent versions of FEP+ using the OPLS4^95^ FF should lead to more accurate results.^10^ However, we refrain from comparing to OPLS4 results as there are no results available using the same input structures.

Table 1 and Figure 1d list the per-target accuracy reached by each FF in terms of ΔΔ*G* RMSE from experimental measurement. The corresponding ΔΔ*G* MUE values can be found in Table S.3 and Figure S.2. This illustrates well that the prediction accuracy is case-dependent. For example, the predicted ΔΔ*G* for GALECTIN in Figure 1 all fall close to the experimentally measured values. Whereas, several other cases, e.g., HIF2A and SHP2, have a widespread distribution of calculated relative free energy differences when compared to the experimental measurement.

**Table 1:** Comparison of the Five Force Fields OpenFF-1.0, OpenFF-2.0, *GAFF*2.1*x*, *CGenFF*/MATCH*, OPLS3e, and the Consensus Approach Based on the RMSE of the ΔΔ*G* Values of the Perturbations^a^

	*N*	RMSE [kcal mol^–^^1^]					
		OpenFF 1.0	OpenFF 2.0	CGenFF/MATCH*	GAFF 2.1x	OPLS 3e	Consensus
ALL	1116	1.7_1.6_^1.8^	1.7_1.6_^1.9^	1.8_1.7_^1.9^	1.7_1.5_^2.0^	1.3_1.3_^1.4^	1.5_1.4_^1.6^
BACE	58	1.0_0.8_^1.2^	1.1_0.9_^1.3^	1.3_1.0_^1.5^	1.1_0.9_^1.3^	1.6_1.3_^1.9^	1.1_0.9_^1.3^
BACE (HUNT)	60	1.1_0.9_^1.3^	1.3_1.0_^1.4^	1.5_1.4_^1.8^	1.2_1.0_^1.4^	0.9_0.8_^1.0^	1.2_1.0_^1.5^
BACE (P2)	26	1.1_0.9_^1.3^	1.2_1.0_^1.3^	1.2_0.8_^1.6^	1.1_0.8_^1.3^	0.8_0.6_^0.9^	1.1_0.8_^1.3^
CDK2	25	1.0_0.8_^1.2^	1.2_0.9_^1.4^	1.0_0.8_^1.4^	0.9_0.6_^1.2^	1.4_0.6_^2.1^	0.9_0.7_^1.1^
CDK8	54	1.7_1.4_^2.0^	1.6_1.3_^1.8^	2.1_1.8_^2.4^	1.2_1.1_^1.5^	1.5_1.3_^1.8^	1.4_1.2_^1.6^
CMET	57	1.9_1.4_^2.6^	2.2_1.3_^3.3^	1.7_1.2_^2.4^	2.1_1.4_^2.9^	1.3_1.1_^1.7^	2.0_1.2_^2.9^
EG5	65	1.7_1.4_^2.2^	1.1_1.0_^1.4^	1.8_1.6_^2.2^	2.1_1.6_^2.5^	1.3_1.1_^1.6^	1.4_1.1_^1.5^
GALECTIN	7	1.0_0.5_^1.4^	0.6_0.3_^0.9^	0.6_0.4_^0.8^	1.0_0.4_^1.6^	0.4_0.1_^0.6^	0.7_0.5_^1.0^
HIF2A	80	2.2_1.8_^2.7^	2.3_1.9_^2.7^	3.5_3.0_^3.8^	2.1_1.8_^2.5^	1.4_1.2_^1.8^	2.1_1.8_^2.5^
JNK1	31	0.9_0.7_^1.2^	1.1_0.8_^1.4^	0.9_0.6_^1.2^	1.0_0.8_^1.4^	0.7_0.6_^0.8^	0.8_0.5_^1.1^
MCL1	71	1.5_1.3_^1.8^	1.6_1.3_^1.9^	1.8_1.6_^2.2^	1.6_1.3_^1.8^	1.4_1.2_^1.6^	1.5_1.3_^1.7^
P38	56	1.3_1.1_^1.6^	1.0_0.8_^1.4^	1.3_1.1_^1.7^	0.9_0.8_^1.1^	1.0_0.8_^1.2^	0.9_0.7_^1.0^
PDE10	59	1.9_1.5_^2.3^	2.9_1.6_^4.2^	2.1_1.8_^2.4^	1.7_1.4_^2.1^	1.7_1.4_^2.1^	1.7_1.4_^2.3^
PDE2	34	1.3_0.9_^1.7^	1.1_0.8_^1.4^	1.5_1.2_^2.0^	1.0_0.7_^1.4^	1.2_0.9_^1.6^	1.2_0.7_^1.7^
PFKFB3	66	1.8_1.6_^2.1^	1.5_1.2_^1.8^	1.6_1.4_^2.1^	1.4_1.1_^1.7^	1.4_1.1_^1.6^	1.4_1.1_^1.6^
PTP1B	49	1.6_1.1_^2.1^	2.3_1.6_^2.7^	1.4_1.0_^1.8^	1.1_0.9_^1.3^	0.8_0.7_^0.9^	1.5_1.1_^1.7^
ROS1	61	2.3_1.8_^3.3^	1.8_1.4_^2.2^	1.3_1.1_^1.6^	1.9_1.5_^2.3^	1.5_1.2_^1.6^	1.6_1.2_^1.9^
SHP2	56	2.6_2.3_^3.1^	2.6_2.0_^3.2^	1.8_1.5_^2.1^	4.3_2.3_^6.1^	1.3_1.1_^1.7^	2.3_1.7_^3.0^
SYK	101	1.3_1.2_^1.5^	1.4_1.1_^1.7^	1.1_1.0_^1.3^	1.4_1.1_^1.5^	1.2_1.1_^1.4^	1.1_0.9_^1.3^
THROMBIN	16	1.3_1.0_^1.6^	1.3_1.1_^1.5^	1.5_0.5_^2.1^	1.0_0.6_^1.2^	1.2_0.9_^1.7^	0.6_0.4_^0.8^
TNKS2	60	0.9_0.7_^1.1^	0.9_0.7_^1.1^	1.6_1.3_^1.9^	0.9_0.7_^1.2^	1.2_0.9_^1.5^	1.0_0.8_^1.3^
TYK2	24	1.1_0.8_^1.5^	1.1_0.9_^1.5^	1.6_1.2_^2.0^	1.3_0.9_^1.6^	1.0_0.7_^1.2^	1.1_0.8_^1.5^

a Each row represents a target set (or “ALL” for all target sets combined) with a specified number *N* of perturbations followed by the RMSE between experimental and calculated values for the respective FF. The upper and lower bounds of the 95% confidence interval are given as sub- and superscript. All values are in kcal mol^–1^. The corresponding table with MUE instead of RMSE can be found in the Supporting Information, Figure S.2.

**Figure 2:**
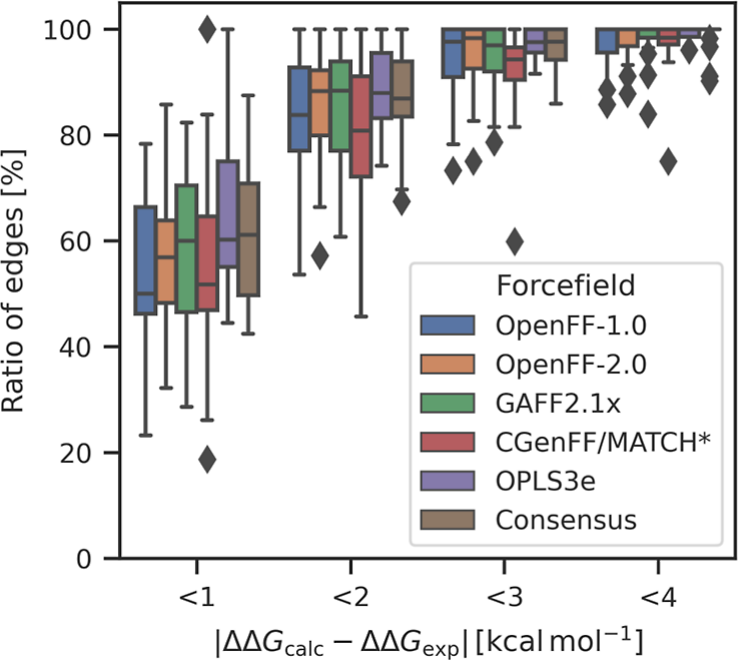
Ratio of calculated ΔΔ*G* within various different absolute error thresholds compared to the experimental value for the different force fields. Box-and-whiskers show the distribution across the various targets. Each box illustrates the first and third quartiles with the median shown as a horizontal bar inside the box and the whiskers are at 1.5× (interquartile range) from the box edges.

Although the aggregated RMSE statistics overall (Figure 1a) or per-target (Figure 1d) do not show a significant difference between the public FFs, the differences become more apparent by looking at the number of outliers. Figure 2 shows the ratio of perturbations with absolute errors versus experiments below a certain threshold. Each box illustrates the distribution across the various targets first and third quartiles, with the median shown as a horizontal bar inside the box, and the whiskers extend up to the minimum (least performing target) and maximum (highest performing target), but at most up to 1.5× (interquartile range) from the box edges (with outliers shown as markers). We observed differences between the FFs in minimum, median, and maximum ratios. For a threshold of 1 kcal mol^–1^ from experiment, the median across targets is at 50% of edges for OpenFF-1.0 and 52% for CGenFF/MATCH*. This median ratio is notably higher for OpenFF-2.0 (57%), GAFF2.1x (60%), OPLS3e (60%), and the consensus approach (61%). Also, the trend of the ratio for the worst performing targets is similar. For the public FFs, the worse performing targets exhibit between 19 and 32% of edges within a 1 kcal mol^–1^ threshold. For OPLS3e and the consensus approach, this ratio is considerably higher at 44 and 42%, respectively.

These trends persist when looking at higher unsigned error thresholds of 2, 3, or 4 kcal mol^–1^.

A strong target dependence of the accuracy of the results can be clearly seen. For OpenFF-1.0 and a threshold of <1 kcal mol^–1^ from the experiment (left blue box in Figure 2), only 23% of the edges agreed with the experiment within the threshold for the worst-performing target (SHP2). On the other hand, 78% of edges in the best-performing target (TNKS2) were correct considering the threshold. This difference between the worst- and best-performing targets can be reduced with the consensus approach, which seems to correct for large outliers. Various reasons can lead to a disproportionate number of outliers for a few targets. One reason can be inaccuracies in the setup of the starting structures. This could be the wrong starting poses of the ligand, inadequate protein preparation, or unlikely protonation or tautomeric states, both in the ligand and in the protein. If all FFs show low performance for a specific target it suggests a common preparation error. The protein and ligands might be more flexible in certain targets, and the free energy estimate only converges if two or more conformational states are sampled sufficiently. Thus, more sampling or even enhanced sampling would be needed to adequately model such a target. Some targets have ligand sets with more difficult perturbations. For example, charge changes, charge redistribution, or the creation/annihilation of large moieties like cyclohexyl groups are difficult perturbations, which either would require longer sampling times, or are even better treated with absolute binding free energy approaches.^96,97^ Some targets might feature certain chemical moieties, which are not adequately described by the respective FF. The use of inadequate parameters may explain why the use of OPLS3e leads to fewer outliers, as the use of custom parameters describes specific chemistries better than a general FF.^75,95,98^ Finally, the experimental results might not be entirely suitable for comparing to calculated binding free energies.^10^ The MD calculations may not mimic the exact experimental conditions (temperature, ion concentrations, and cosolvents), or the assay may only have limited correlation with the binding free energy that is targeted in the RBFE calculations. But this has a limited impact when comparing the different FFs, as they are all compared to the same data.

#### Accuracy of Predicted Δ*G*

**Figure 3:**
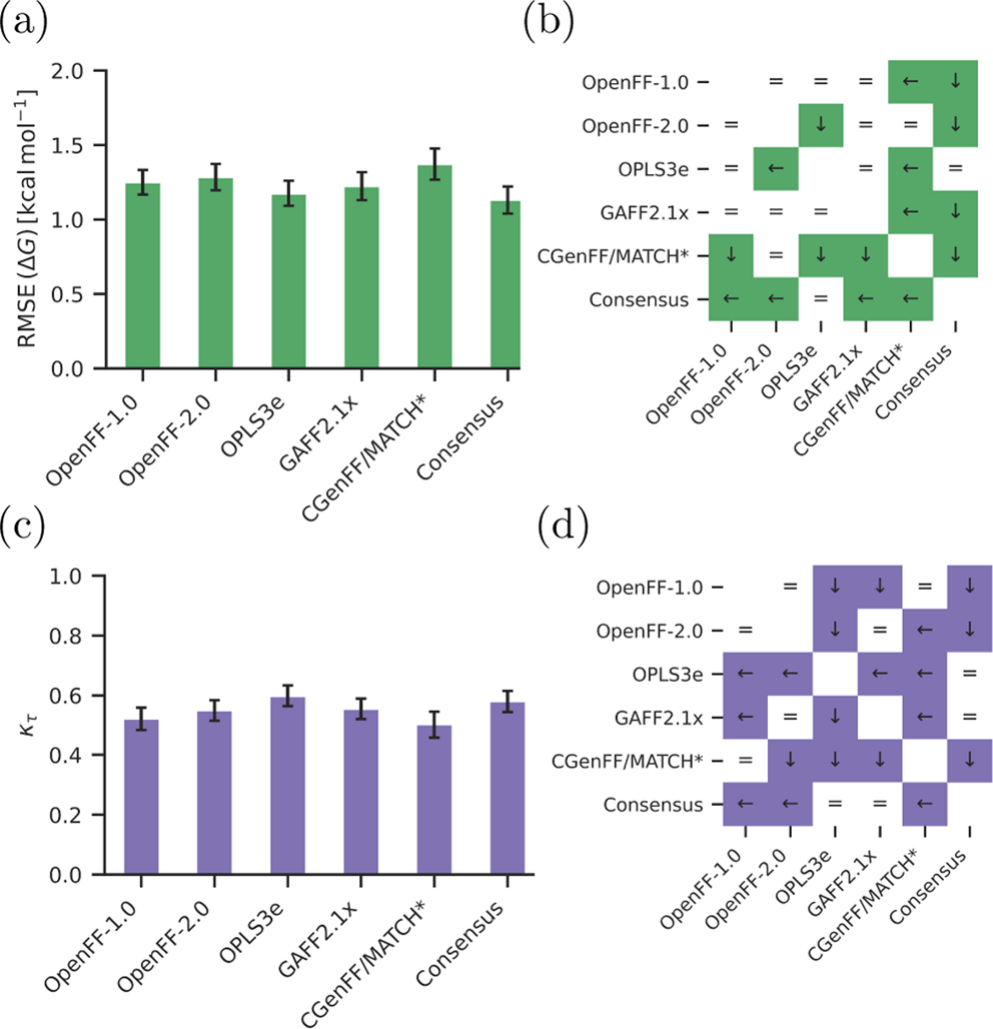
Comparison of Δ*G* values of the ligands obtained from calculations using the five force fields OpenFF-1.0, OpenFF-2.0, GAFF2.1x, CGenFF/MATCH*, and OPLS3e and the consensus approach. (a) RMSE comparison across all targets and 598 ligands. (b) Illustrations of significance of differences between the different sets. (c) Comparison of ranking metric τ_K_ across all targets and 598 ligands. (d) Illustrations of significance of differences between the different sets. Colors denote the different metrics (green for RMSE and purple for τ_K_). In panels (b) and (d), a white matrix element with an equal sign (“=”) means that the differences between the two force fields are statistically insignificant. Colored matrix element means there is a significant difference considering a 95% confidence interval. Arrow in a colored matrix element points at the force field, which has the lower error (either left or down).

Figure 3 shows the trend in significant differences between FFs changes when comparing accuracy in terms of back-calculated absolute binding free energies Δ*G*. In this analysis, in terms of RMSE to experimental measurement, OPLS3e still significantly outperforms OpenFF-2.0 and CGenFF/MATCH*; however, its difference to OpenFF-1.0 and GAFF2.1x is no longer significant (Figure 3a,b). The consensus approach outperforms the individual open source FFs, similarly as it was for the ΔΔ*G* comparison.

We also compared FF predictions in terms of their ability to correctly rank binders based on their Δ*G* values by using Kendall’s τ_K_ correlation coefficient (τ_K_). This measure again reveals the same two variants outperforming the others—OPLS3e and the consensus approach. While the pattern of significant differences between FFs is rather complex (Figure 3d), the differences are small in magnitude, showing that each of the FFs can be trusted to yield a compound ranking of similar quality. The Supporting Information, Figures S.5–S.8 illustrate aggregated statistics based on Δ*G* per target and across all targets for all the FFs, including the consensus approaches. The corresponding values can be found in the Supporting Information, Tables S.6–S.9. Additionally, correlation plots are provided for OpenFF-2.0, CGenFF/MATCH*, GAFF2.1x, OPLS3e, and the consensus approach in the Supporting Information, Figures S.9–S.12.

#### Determinants of the Prediction Accuracy

**Figure 4:**
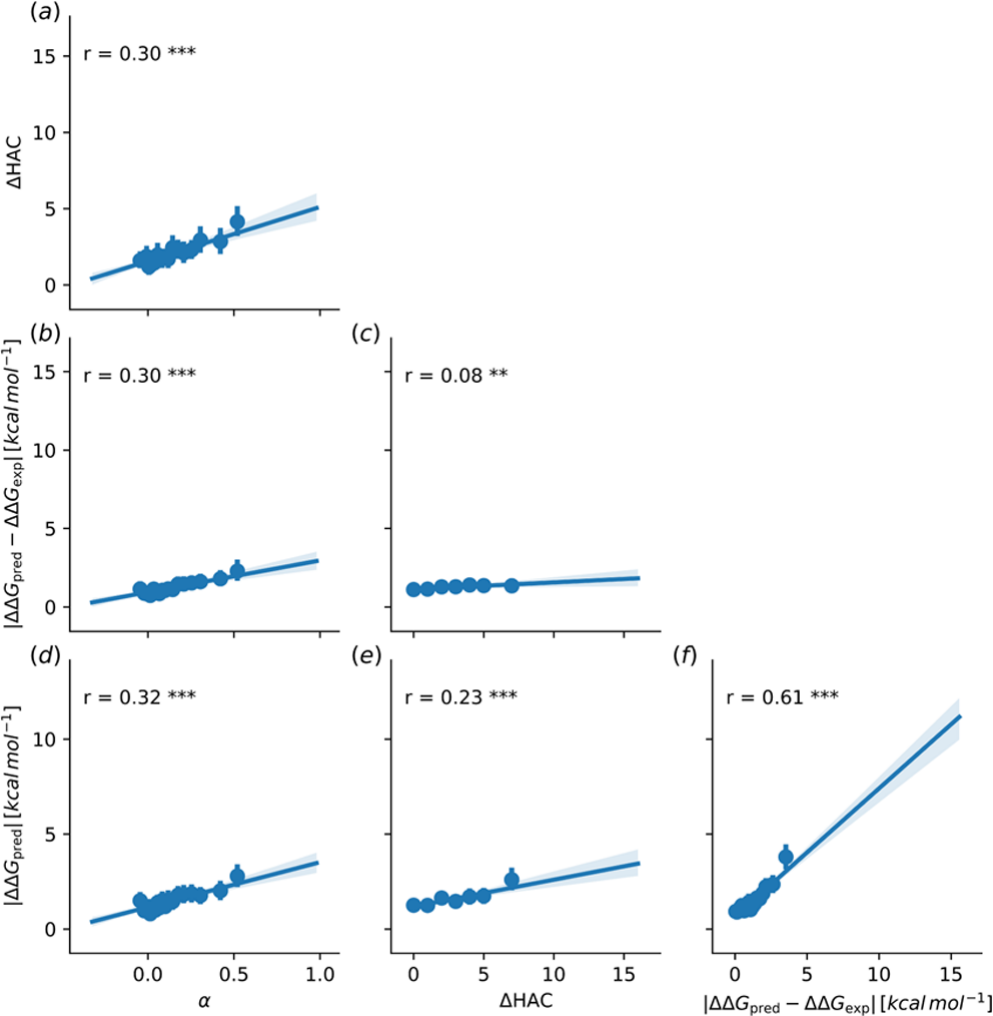
Visualization of pairwise relationships between the change in number of heavy atoms in the end states, the absolute error between experimental and calculated values |ΔΔ*G*_pred_ – ΔΔ*G*_exp._|, the calculated relative free energies ΔΔ*G*_pred_ (OpenFF-2.0), and the average convergence measure α^92^ (averaged over three solvent and three complex simulation legs). Subplots show linear regression plots between the respective properties. Pearson’s correlation coefficient is given in the graph together with its *p*-value indicated as stars (one, two, or three stars for the confidence level of <0.05, <0.01, and <0.001, respectively). For illustration purposes, the data was binned into 20 bins and their average with standard deviation are shown as dot with error bars. Regression was performed on the original data. Panels a, c, d, and f mark the trends described in the text. More detailed illustration of this figure is shown in the Supporting Information, Figure S.17.

There are numerous underlying causes for the differences in accuracy in addition to the small molecule FF, e.g., sampling, specifics of the calculation procedure, and initial system setup. In the analysis in Figure 4, we attempted to elucidate the main determinants underlying ΔΔ*G* prediction accuracy related to the convergence of an alchemical perturbation.

In particular, we noticed that larger calculated ΔΔ*G* values are associated with a larger error (Figure 4f). Namely, the alchemical approach can be expected to become less accurate when the predicted change in free energy of binding is large. This effect is in turn explained by the difficulty in converging such perturbations: predicted large free energy differences correlate with the lack in convergence of the estimates (Figure 4d). While there are many factors influencing the convergence of an alchemical perturbation, we observed that a simple count of heavy atoms that need to be introduced/annihilated shows a low, but statistically significant correlation (Pearson’s *r* = 0.08, *p*-value <0.01) with the absolute error (Figure 4c) and larger correlation with the convergence measure (Figure 4a). Similar trends as for the heavy atom count can be seen in the Supporting Information for the counts of rotatable bonds (Figure S.22), counts of rings (Figure S.23), changes or positions of the formal charges (Figure S.24), and the LOMAP score^26^ (Figure S.25). In Figure 4, we used the ΔΔ*G* values and convergence metric α of the simulations using OpenFF-2.0. Although the edges might show different levels of convergence between the FFs (Figure S.13), overall we found that the ratios of converged simulations differ insignificantly among OpenFF-1.0, OpenFF-2.0, GAFF2.1x, and CGenFF/MATCH* (Figure S.14). Moreover, we observed the same trends as described above for OpenFF-2.0 for the results calculated with OpenFF-1.0 (Figure S.16), GAFF2.1x (Figure S.18), and CGenFF/MATCH* (Figure S.19).

All in all, this simple trace through the dependencies in the data already reveals some of the determinants limiting the accuracy of our predictions. For larger perturbations, the calculation convergence suffers, thus reducing the agreement between the prediction and experiment. It is important to note, however, that the identified signal is noisy, i.e., not every large perturbation will be inaccurate and not all well converged simulations will yield perfect binding free energy predictions. The identified determinants for prediction accuracy are only general trends in a complex picture.

In addition to these factors, the accuracy of the prediction will also be influenced by the technical setup of the calculation procedure. For example, it has been observed that even file conversion by different software packages may introduce artifacts in molecular structure.^88^ Also, combining small molecule FFs with disparate charge models will have an effect on the prediction accuracy.^100,101^ Differences between simulation packages^102^ and free energy protocols^103^ will influence the sampling and, subsequently, the final free energy estimates. Considering the limited sampling used in the standard free energy calculation protocols, the starting structure quality often affects the prediction accuracy.^12−14^

### OpenFF Improvement

#### Nonconverged Results Are Less Accurate

**Figure 5:**
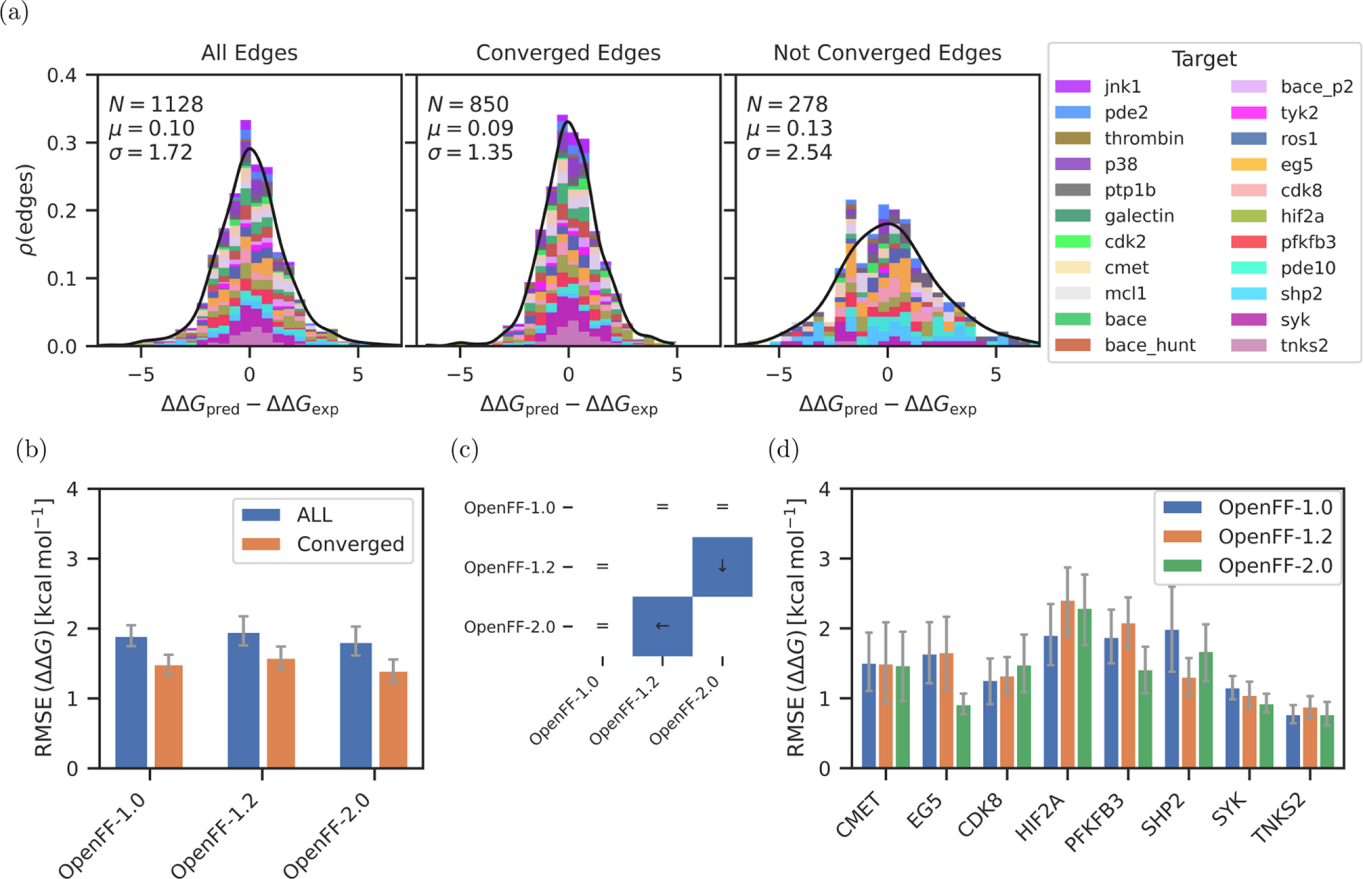
Comparison of the three force fields OpenFF-1.0, OpenFF-1.2, and OpenFF-2.0 based on the ΔΔ*G* values. Panel (a) shows the absolute error distributions between experimental and calculated ΔΔ*G* using OpenFF-2.0 for three sets of edges. First set in the left subpanel contains all edges, the second set in the center contains only converged edges, and the third set in the right contains the not converged edges (which is the difference set between the first and second set). See the Methods Section for more details about the convergence criteria. Different colors denote the different targets and the black line is a normal distribution fitted to the data. Text in the panel lists the number of edges *N*, the center μ, and the standard deviation σ of the normal distribution. Panel (b) shows the RMSE across all edges of 8 targets for the three force fields of the OpenFF family. Blue bars correspond to all edges and the orange bars only to the converged ones. Panel (c) illustrates significant differences between the force field sets shown in panel (b). White matrix element with an equal sign (“=”) means that the differences between the two force fields are statistically insignificant. Blue matrix element denotes a significant difference considering a 95% confidence interval. Arrow in a blue matrix element points at the force field, which has the lower error. Panel (d) shows the RMSE of the ΔΔ*G* values per target for the three force fields OpenFF-1.0, OpenFF-1.2, and OpenFF-2.0. Lower and upper bound of the 95% confidence interval are given as error bars. All values are in kcal mol^–1^.

The difference between the set of all results and the converged set is illustrated in Figure 5a as histograms of deviations between experimental and calculated values (see the Methods Section for details about the convergence criteria). Whereas all edges consisting of converged and nonconverged perturbations show a large standard deviation of 1.72 kcal mol^–1^, the filtered set of 850 converged edges has a reduced standard deviation of 1.35 kcal mol^–1^, while the remaining 278 not converged edges are enriched in outliers resulting in a larger standard deviation of 2.54 kcal mol^–1^. The convergence criteria can therefore be used to flag calculations, which are likely to have larger errors without prior knowledge of experimental results.

Figure 5b,d and Table 2 compare three OpenFF versions by means of RMSE between calculated and experimental ΔΔ*G* values for results obtained on a subset of 551 perturbations (of which 340 are converged) in eight different targets. While the intermediate version OpenFF-1.2 did not show an improvement over OpenFF-1.0, OpenFF-2.0 significantly improved in accuracy compared to the previous OpenFF-1.2 (Figure 5c). This trend holds both for all edges and the converged set of edges.

**Table 2:** Comparison of the Three Force Fields OpenFF-1.0, OpenFF-1.2, and OpenFF-2.0 Based on the RMSE of the ΔΔ*G* Values of the Converged Perturbations^a^

	*N*	RMSE [kcal mol^–^^1^]		
		OpenFF 1.0	OpenFF 1.2	OpenFF 2.0
ALL	320	1.5_1.4_^1.6^	1.5_1.4_^1.7^	1.4_1.2_^1.6^
CDK8	27	1.3_0.9_^1.6^	1.3_1.1_^1.6^	1.4_1.1_^1.9^
CMET	35	1.5_1.1_^2.0^	1.5_0.9_^2.1^	1.4_1.0_^1.9^
EG5	29	1.6_1.2_^2.1^	1.6_1.2_^2.1^	0.9_0.8_^1.1^
HIF2A	45	1.8_1.5_^2.3^	2.4_1.9_^2.9^	2.3_1.8_^2.8^
PFKFB3	42	1.9_1.5_^2.2^	2.0_1.7_^2.4^	1.4_1.1_^1.7^
SHP2	17	1.9_1.4_^2.5^	1.3_1.0_^1.6^	1.7_1.3_^2.1^
SYK	74	1.7_1.3_^2.1^	1.0_0.9_^1.2^	0.9_0.8_^1.1^
TNKS2	51	0.8_0.6_^0.9^	0.9_0.7_^1.0^	0.8_0.6_^1.0^

a Each row represents a target set (or “all” for all target sets combined) with a specified number *N* of perturbations followed by the RMSE between experimental and calculated values for the respective FF. The upper and lower bounds of the 95% confidence interval are given as sub- and superscript. All values are in kcal mol^–1^. The values are illustrated in Figure 5b,d.

#### Effect of Force Field Parameter Change from OpenFF-1.0 to OpenFF-2.0

**Figure 6:**
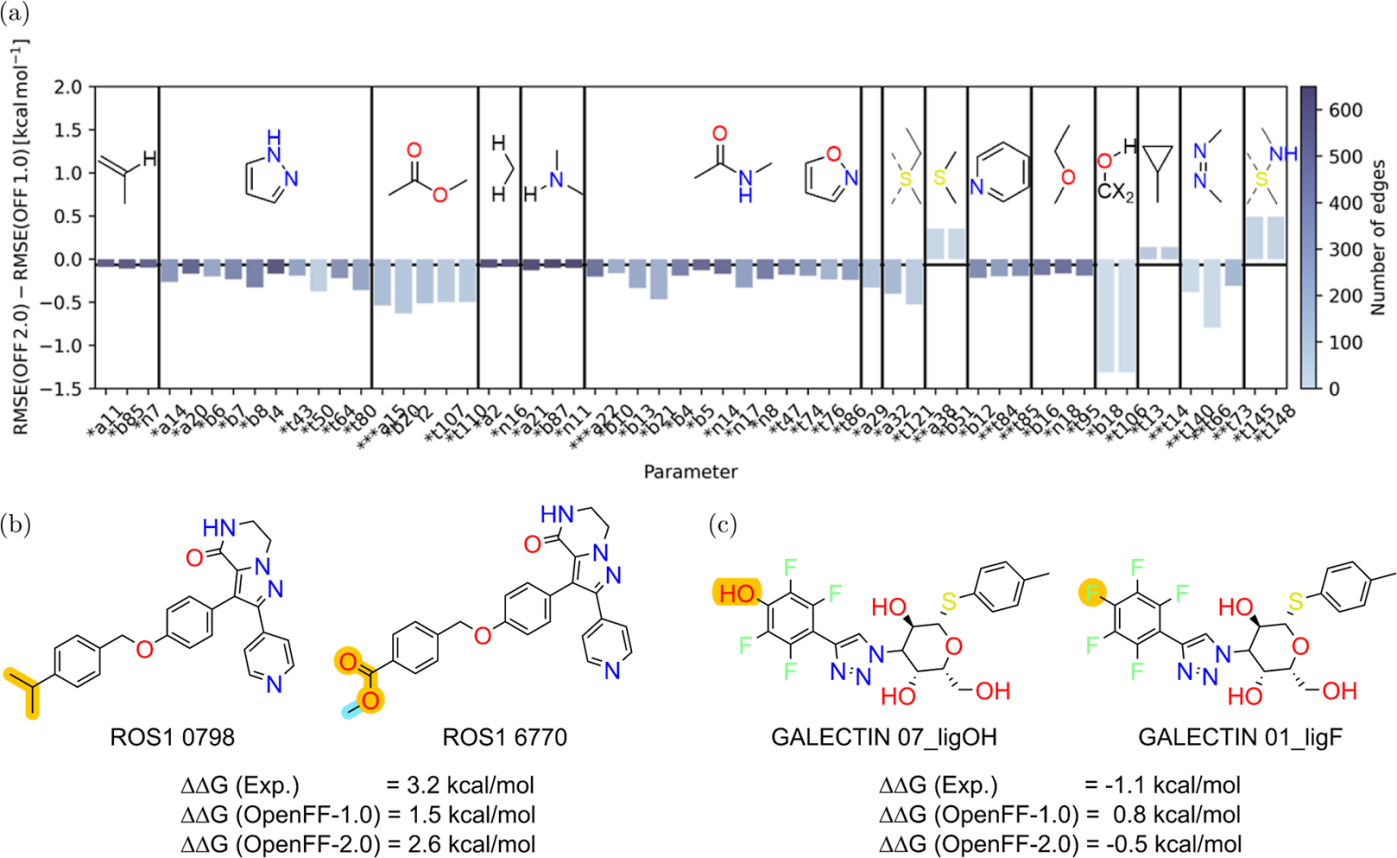
Analysis of parameter differences between OpenFF-1.0 and OpenFF-2.0. Panel (a) shows the RMSE difference between OpenFF-1.0 and OpenFF-2.0 for subsets of converged edges, where a certain parameter is perturbed (*x*-axis) and the difference between OpenFF-1.0 and OpenFF-2.0 is significant (CI 95%). Stars (*) in front of the parameter label denote how much the parameter changed between OpenFF-1.0 and OpenFF-2.0 (3 stars denote the largest change). Horizontal black line denotes the insignificant difference (−0.06 kcal mol^–1^) for the whole set of perturbations. Vertical bars separate groups of bars with high correlations, i.e., they are usually employed concurrently in perturbations. Chemical structure shows an example substructure where each group of parameters is employed. Panel (b) shows an example perturbation where an ester function (third group in panel (a)) is introduced. Free energy prediction improved from ΔΔ*G* = 1.5 kcal mol^–1^ (OpenFF-1.0) to ΔΔ*G* = 2.6 kcal mol^–1^ (OpenFF-2.0) with an experimental value of ΔΔ*G* = 3.2 kcal mol^–1^. Panel (c) shows an example perturbation where an aromatic hydroxy group (fourth group from the right in panel (a)) is morphed into a fluorine atom. Free energy prediction improved from ΔΔ*G* = 0.8 kcal mol^–1^ (OpenFF-1.0) to ΔΔ*G* = −0.5 kcal mol^–1^ (OpenFF-2.0) with an experimental value of ΔΔ*G* = −1.1 kcal mol^–1^. In panels (b) and (c), the perturbed atoms and bonds are highlighted in orange, whereas annihilated atoms and bonds are highlighted in cyan.

In Figure 6a, we highlight FF parameter changes between two OpenFF versions, 1.0 and 2.0, and their effect on the predicted free energy accuracy for the cases where the effect is statistically significant. In these cases, various other factors influencing the accuracy like starting conformations and convergence cannot be the cause for the difference; therefore, it is more likely that the underlying reason is the FF parameters. For example, an ester group is described by its angle (OpenFF code a15), bond (b20), improper (i2), and torsion (t107, t110) parameters, which were modified between the OpenFF releases. Altogether, the RMSE between the predicted and experimental ΔΔ*G* for the perturbations of the ester groups drops by 0.5 kcal mol^–1^ when going from OpenFF-1.0 to OpenFF-2.0 (Figure 6a). An example for a perturbation involving an ester group is shown in Figure 6b: in this case, the new OpenFF-2.0 parameters led to a reduction in the error of ΔΔ*G* by 1.1 kcal mol^–1^.

Similar trends are observed for the other significant changes in FF parameters: the predicted free energy difference is more accurate for the modified parameters. The largest improvement in this analysis was observed for the changes in the hydroxyl group bound to a sp^2^ carbon involving the bond (b18) and torsion (t106) parameters. Figure 6c illustrates a case where this improvement resulted in 1.3 kcal mol^–1^ increase in free energy calculation accuracy.

There are only a few parameter groups that result in decreased ΔΔ*G* prediction accuracy for OpenFF-2.0 compared to OpenFF-1.0. Namely, changes in parameters describing sulfur-containing groups like thioethers (a38, b51) or sulfonamides (t145, t148) and torsions (t13 and t14) describing cyclopropyl groups appear to have a detrimental effect on binding affinity accuracy.

The improvement of free energy results related to parameter changes is remarkable as the parameters were designed on the condensed phase and QM properties of small molecules. We show that improving the latter properties also has a positive and significant effect on the downstream free energy of binding calculation results.

## Conclusions

On a set of 598 ligands each binding to one of 22 targets, we showed that the public FFs OpenFF-1.0 (Parsley), OpenFF-2.0 (Sage), GAFF2.1x, and CGenFF/MATCH* are performing comparably based on aggregated statistics across the whole dataset, both in terms of the RMSE of relative binding free energies ΔΔ*G* (perturbations) and the RMSE and Kendall’s tau of binding free energies Δ*G*. The proprietary FF OPLS3e performs significantly better, but a consensus approach based on Sage, GAFF2.1x, and CGenFF/MATCH* is similarly accurate based on Δ*G* regarding the RMSE and Kendall’s τ. There is a clear target dependence, which can be attributed to input preparation, protein (binding pocket) flexibility, chemistry of ligands, and difficulty of perturbations (in terms of heavy atom changes). While Parsley and Sage are performing comparably based on aggregated statistics across the whole dataset, there are differences in terms of outliers. A parameter analysis revealed that improved parameters lead to significant improvement in the accuracy of affinity predictions on more than 50 subsets of the dataset involving those parameters, while six subsets involving certain parameters showed lower accuracy. Thus, we can show that there is a considerable improvement of successive OpenFF versions.

In the future, such a parameter analysis can be used to identify potentially problematic parameters, which can then be investigated and improved for next FF versions. Indeed, this study also allowed us to identify parameters in well converged but inaccurate perturbations, along with further calculations, this provides future investigation and possible avenues for FF improvement. However, for this to be successful, further work would be valuable to reduce the influence of other (non FF parameter) sources of errors like large or difficult perturbations, inadequate input preparation, or insufficient sampling.

## Data Availability

The used input structures, molecular dynamics topologies, and experimental data are provided as a Zenodo record (https://zenodo.org/records/10782775)^104^ or can be retrieved from the protein–ligand benchmark GitHub repository, release 0.2.1 (https://github.com/openforce-field/protein-ligand-benchmark/).^59^ Simulation input was prepared, and trajectories were analyzed with pmx, which is freely available on GitHub (https://github.com/deGrootLab/pmx).^60^ Corresponding input parameter files and workflow scripts used to prepare the simulations are also available on GitHub (https://github.com/dfhahn/pmx) or as a Zenodo record.^105,106^ The simulations were run with Gromacs (http://gromacs.org/).^107^ The analysis code and code to create figures are provided in the protein–ligand benchmark analysis repository, release 0.3.0 (https://github.com/dfhahn/protein-ligand-benchmark-analysis).^90^

## Supporting Information Available

Details about the employed target set, additional graphs and tables containing aggregated statistics and correlations with experiment in greater detail, and analysis of various properties of the simulated perturbations (PDF)
