# Supplementary material for: Current State of Open Source Force Fields in Protein–Ligand Binding Affinity Predictions
Source: J Chem Inf Model. 2024 Jun 19;64(13):5063–76. doi: 10.1021/acs.jcim.4c00417 (PMC11234369; doi:10.1021/acs.jcim.4c00417)
Supplement: Supplementary file 1 — ci4c00417_si_001.pdf [file ci4c00417_si_001.pdf]

Supporting Information
Current state of open source force fields in protein-ligand binding affinity predictions

David F. Hahn,[*,†] Vytautas Gapsys,[‡,†] Bert L. de Groot,[‡] David L. Mobley,[¶,§] and Gary Tresadern[†]

†*Computational Chemistry, Janssen Research & Development, Turnhoutseweg 30, 2340 Beerse, Belgium*

‡*Computational Biomolecular Dynamics Group, Max Planck Institute for Multidisciplinary Sciences, Am Fassberg 11, 37077 Göttingen, Germany*

¶*Department of Chemistry, University of California, Irvine, CA 92697*

§*Department of Pharmaceutical Sciences, University of California, Irvine, CA 92697*

E-mail: dhahn3@its.jnj.com

Dataset

Table S.1: *Summary and detailed data provenance for the benchmark set employed in here.* The successive entries are the target name/identifier, information about the crystal structures used, the source of the initial coordinates, ligand count, dynamic range and standard deviation of the activities, the number of perturbations, and the provenance of the calculation results. For each structure, the PDB ID is followed by the Iridium classification and Iridium score in the brackets. The Iridium classification categorizes each structure into not trustworthy (NT), medium trustworthy (MT) and highly trustworthy (HT) categories. The lower the Iridium score, the better the structure.[30] Also, the diffraction-component precision indeces (DPI) are listed. For the activity data ("Ligand Information"), the number of ligands (count), the dynamic range ($\max(\Delta G) - \min(\Delta G)$) and the standard deviation of the ΔG ($\mathrm{std}(\Delta G)$) are given. The letters G, B, S, and T for the provenance of input coordinates and calculation results stand for: G: Gapsys *et al.*;[31] P: Perez Benito *et al.*;[24] S: Schindler *et al.*[9] and T: this work.

Target	Used structure PDB	DPI	Input coordinates	Ligand Information Count	Dyn. Range [kcal mol^{-1}]	std(DG) [kcal mol^{-1}]	Edges Count	Calculations OpenFF all versions	GAFF2.1	GAFF2.11	CGenFF/MATCH*	OPLS3e
BACE[1,2]	4DJW (HT , 0.32)	0.11	G	36	3.9	0.8	58	T	G		G	G
BACE_HUNT[3–5]	4JPC (HT , 0.32)	0.12	G	32	4.9	1.2	60	T	G		G	G
BACE_P2[5,6]	3IN4 (HT , 0.59)	0.28	G	12	0.9	0.3	26	T	G		G	G
CDK2[2,7]	1H1Q (MT, 0.87)	0.28	G	16	4.3	1.2	25	T	G		G	G
CDK8[8,9]	5HNB (MT, 0.74)	0.22	S	33	5.7	1.3	54	T		T	T	S
c-MET[10]	4R1Y (MT,[h] 0.75)	0.17	S	24	6.2	1.7	57	T		T	T	S
EG5[9,11]	3L9H (MT, 0.88)	0.18	S	28	3.5	0.9	65	T		T	T	S
Galectin[12,13]	5E89 (MT, 1.04)	0.07	G	8	2.7	0.8	7	T	G		G	G
HIF2a[9,14]	5TBM (HT , 0.35)	0.17	S	42	4.6	1.1	80	T		T	T	S
Jnk1[2,15]	2GMX (NT ,)	0.77	G	21	3.4	0.8	31	T	G		G	G
MCL1[2,16]	4HW3 (HT , 0.41)	0.26	G	42	4.2	1.1	71	T	G		G	G
P38[2,17]	3FLY (HT , 0.6)	0.12	G	34	3.8	1.0	56	T	G		G	G
PDE2[18,19]	6EZF (MT, 0.3)	0.07	G	21	3.2	0.9	34	T	G		G	G
PDE10[20]	-[i] (- , -)	-	P	34			59	T		T	T	P
PFKFB3[9,21]	6HVI (HT , 0.31)	0.11	S	40	3.7	1.1	66	T		T	T	S
PTP1B[2,22]	2QBS (MT, 0.33)	0.15	G	16	4.3	1.2	49	T	G		G	G
SHP2[9,23]	5EHR (MT, 0.32)	0.1	S	26	4.3	1.2	56	T		T	T	G
ROS1[24]	-[i] (- , -)		P	28			63	T		T	T	P
SYK[9,25]	4PV0 (MT, 0.69)	0.19	S	44	6.0	1.0	101	T		T	T	S
Thrombin[2,26]	2ZFF (HT , 0.3)	0.06	G	11	1.7	0.5	16	T	G		G	G
TNKS2[27]	4UI5 (HT , 0.29)	0.08	S	27	4.3	1.0	60	T		T	T	S
TYK2[2,28,29]	4GIH (HT , 0.5)	0.15	G	16	4.3	1.3	24	T	G		G	G
all	(,)		-	598			1116					

G: Gapsys *et al.*[31]
P: Perez Benito *et al.*[24]
S: Schindler *et al.*[9]
T: This work

[a] structure was already available 6 month prior to publication of first benchmark study
[b] ligand similarity ¿ 1.4
[c] ligand considerably similar ¿ 0.8
[d] crystal contacts
[e] packing
[g] alternate conformations
[h] ligand density: 0.79
[i] Structure not deposited in the PDB, but available in the protein ligand benchmark set[32]

Performance of force fields based on $\Delta\Delta G$

All edges and all force fields

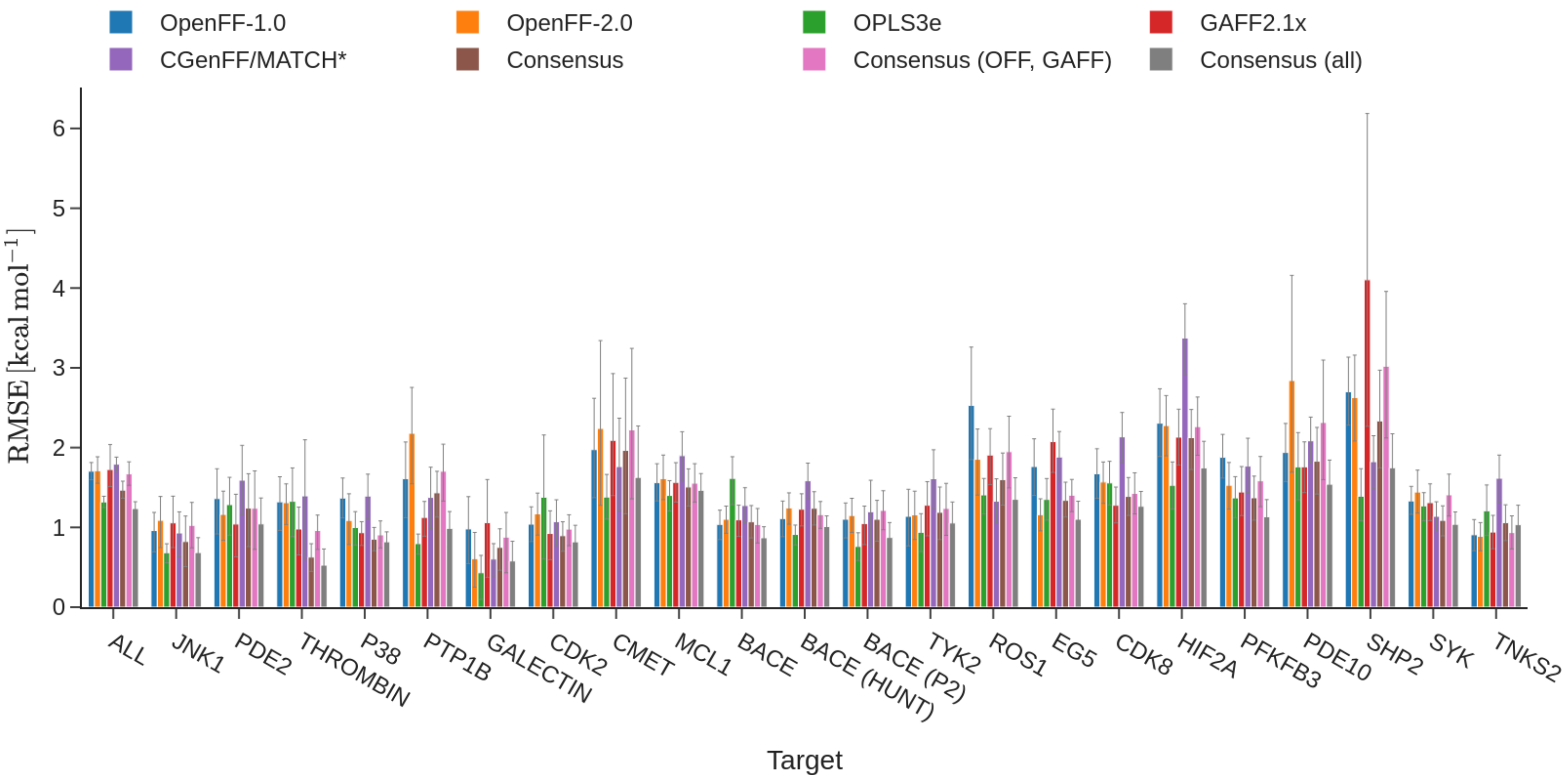

Figure S.1: *Comparison of the five force fields OpenFF-1.0, OpenFF-2.0, GAFF2.1x, CGenFF/MATCH*, OPLS3e and three consensus approaches based on the root mean squared error (RMSE) of the $\Delta\Delta G$ values of the perturbations.* Each group represents a target set (or 'all' for all target sets combined) with the RMSE values between experimental and calculated value for the respective force fields in different colors. The lower and upper bound of the 95% confidence interval are given as error bars. All values are in kcal mol^{-1}.

Table S.2: *Comparison of the five force fields OpenFF-1.0, OpenFF-2.0, GAFF2.1x, CGenFF/MATCH*, OPLS3e and three consensus approaches based on the root mean squared error (RMSE) of the* $\Delta\Delta G$ *values of the perturbations.* Each row represents a target set (or 'all' for all target sets combined) with a specified number N of perturbations followed by the RMSE between experimental and calculated value for the respective force field. The upper and lower and upper bound of the 95% confidence interval are given as sub- and superscript. All values are in kcal mol^{-1}.

	N	RMSE [kcal mol^{-1}]							
		OpenFF -1.0	OpenFF -2.0	CGenFF/ MATCH*	GAFF 2.1x	OPLS 3e	Consensus	Consensus (OFF, GAFF)	Consensus (all)
ALL	1116	$1.7^{1.8}_{1.6}$	$1.7^{1.9}_{1.6}$	$1.8^{1.9}_{1.7}$	$1.7^{2.0}_{1.5}$	$1.3^{1.4}_{1.3}$	$1.5^{1.6}_{1.4}$	$1.7^{1.8}_{1.5}$	$1.2^{1.3}_{1.2}$
BACE	58	$1.0^{1.2}_{0.8}$	$1.1^{1.3}_{0.9}$	$1.3^{1.5}_{1.0}$	$1.1^{1.3}_{0.9}$	$1.6^{1.9}_{1.3}$	$1.1^{1.3}_{0.9}$	$1.0^{1.3}_{0.8}$	$0.9^{1.0}_{0.7}$
BACE (HUNT)	60	$1.1^{1.3}_{0.9}$	$1.3^{1.4}_{1.0}$	$1.5^{1.8}_{1.4}$	$1.2^{1.4}_{1.0}$	$0.9^{1.0}_{0.8}$	$1.2^{1.5}_{1.0}$	$1.1^{1.3}_{1.0}$	$1.0^{1.2}_{0.9}$
BACE (P2)	26	$1.1^{1.3}_{0.9}$	$1.2^{1.3}_{1.0}$	$1.2^{1.6}_{0.8}$	$1.1^{1.3}_{0.8}$	$0.8^{0.9}_{0.6}$	$1.1^{1.3}_{0.8}$	$1.2^{1.4}_{1.0}$	$0.9^{1.1}_{0.7}$
CDK2	25	$1.0^{1.2}_{0.8}$	$1.2^{1.4}_{0.9}$	$1.0^{1.4}_{0.8}$	$0.9^{1.2}_{0.6}$	$1.4^{2.1}_{0.6}$	$0.9^{1.1}_{0.7}$	$1.0^{1.2}_{0.8}$	$0.8^{1.0}_{0.6}$
CDK8	54	$1.7^{2.0}_{1.4}$	$1.6^{1.8}_{1.3}$	$2.1^{2.4}_{1.8}$	$1.2^{1.5}_{1.1}$	$1.5^{1.8}_{1.3}$	$1.4^{1.6}_{1.2}$	$1.4^{1.7}_{1.1}$	$1.2^{1.5}_{1.1}$
CMET	57	$1.9^{2.6}_{1.4}$	$2.2^{3.3}_{1.3}$	$1.7^{2.4}_{1.2}$	$2.1^{2.9}_{1.4}$	$1.3^{1.7}_{1.1}$	$2.0^{2.9}_{1.2}$	$2.3^{3.2}_{1.3}$	$1.6^{2.3}_{1.1}$
EG5	65	$1.7^{2.2}_{1.4}$	$1.1^{1.4}_{1.0}$	$1.8^{2.2}_{1.6}$	$2.1^{2.5}_{1.6}$	$1.3^{1.6}_{1.1}$	$1.4^{1.5}_{1.1}$	$1.4^{1.6}_{1.2}$	$1.1^{1.3}_{0.9}$
GALECTIN	7	$1.0^{1.4}_{0.5}$	$0.6^{0.9}_{0.3}$	$0.6^{0.8}_{0.4}$	$1.0^{1.6}_{0.4}$	$0.4^{0.6}_{0.1}$	$0.7^{1.0}_{0.5}$	$0.8^{1.2}_{0.5}$	$0.6^{0.8}_{0.3}$
HIF2A	80	$2.2^{2.7}_{1.8}$	$2.3^{2.7}_{1.9}$	$3.5^{3.8}_{3.0}$	$2.1^{2.5}_{1.8}$	$1.4^{1.8}_{1.2}$	$2.1^{2.5}_{1.8}$	$2.3^{2.6}_{1.9}$	$1.7^{2.0}_{1.4}$
JNK1	31	$0.9^{1.2}_{0.7}$	$1.1^{1.4}_{0.8}$	$0.9^{1.2}_{0.6}$	$1.0^{1.4}_{0.8}$	$0.7^{0.8}_{0.6}$	$0.8^{1.1}_{0.5}$	$1.0^{1.3}_{0.7}$	$0.7^{0.9}_{0.5}$
MCL1	71	$1.5^{1.8}_{1.3}$	$1.6^{1.9}_{1.3}$	$1.8^{2.2}_{1.6}$	$1.6^{1.8}_{1.3}$	$1.4^{1.6}_{1.2}$	$1.5^{1.7}_{1.3}$	$1.6^{1.8}_{1.3}$	$1.5^{1.7}_{1.3}$
P38	56	$1.3^{1.6}_{1.1}$	$1.0^{1.4}_{0.8}$	$1.3^{1.7}_{1.1}$	$0.9^{1.1}_{0.8}$	$1.0^{1.2}_{0.8}$	$0.9^{1.0}_{0.7}$	$0.9^{1.1}_{0.7}$	$0.8^{0.9}_{0.7}$
PDE10	59	$1.9^{2.3}_{1.5}$	$2.9^{4.2}_{1.6}$	$2.1^{2.4}_{1.8}$	$1.7^{2.1}_{1.4}$	$1.7^{2.1}_{1.4}$	$1.7^{2.3}_{1.4}$	$2.2^{3.1}_{1.6}$	$1.5^{1.8}_{1.2}$
PDE2	34	$1.3^{1.7}_{0.9}$	$1.1^{1.4}_{0.8}$	$1.5^{2.0}_{1.2}$	$1.0^{1.4}_{0.7}$	$1.2^{1.6}_{0.9}$	$1.2^{1.7}_{0.7}$	$1.2^{1.8}_{0.7}$	$1.0^{1.4}_{0.7}$
PFKFB3	66	$1.8^{2.1}_{1.6}$	$1.5^{1.8}_{1.2}$	$1.6^{2.1}_{1.4}$	$1.4^{1.7}_{1.1}$	$1.4^{1.6}_{1.1}$	$1.4^{1.6}_{1.1}$	$1.6^{1.9}_{1.3}$	$1.1^{1.3}_{0.9}$
PTP1B	49	$1.6^{2.1}_{1.1}$	$2.3^{2.7}_{1.6}$	$1.4^{1.8}_{1.0}$	$1.1^{1.3}_{0.9}$	$0.8^{0.9}_{0.7}$	$1.5^{1.7}_{1.1}$	$1.7^{2.1}_{1.3}$	$1.0^{1.2}_{0.8}$
ROS1	61	$2.3^{3.3}_{1.8}$	$1.8^{2.2}_{1.4}$	$1.3^{1.6}_{1.1}$	$1.9^{2.3}_{1.5}$	$1.5^{1.6}_{1.2}$	$1.6^{1.9}_{1.2}$	$2.0^{2.4}_{1.5}$	$1.3^{1.6}_{1.1}$
SHP2	56	$2.6^{3.1}_{2.3}$	$2.6^{3.2}_{2.0}$	$1.8^{2.1}_{1.5}$	$4.3^{6.1}_{2.3}$	$1.3^{1.7}_{1.1}$	$2.3^{3.0}_{1.7}$	$3.1^{3.9}_{2.2}$	$1.7^{2.2}_{1.4}$
SYK	101	$1.3^{1.5}_{1.2}$	$1.4^{1.7}_{1.1}$	$1.1^{1.3}_{1.0}$	$1.4^{1.5}_{1.1}$	$1.2^{1.4}_{1.1}$	$1.1^{1.3}_{0.9}$	$1.4^{1.7}_{1.2}$	$1.0^{1.2}_{0.9}$
THROMBIN	16	$1.3^{1.6}_{1.0}$	$1.3^{1.5}_{1.1}$	$1.5^{2.1}_{0.5}$	$1.0^{1.2}_{0.6}$	$1.2^{1.7}_{0.9}$	$0.6^{0.8}_{0.4}$	$0.9^{1.2}_{0.7}$	$0.5^{0.7}_{0.3}$
TNKS2	60	$0.9^{1.1}_{0.7}$	$0.9^{1.1}_{0.7}$	$1.6^{1.9}_{1.3}$	$0.9^{1.2}_{0.7}$	$1.2^{1.5}_{0.9}$	$1.0^{1.3}_{0.8}$	$0.9^{1.2}_{0.7}$	$1.0^{1.2}_{0.8}$
TYK2	24	$1.1^{1.5}_{0.8}$	$1.1^{1.5}_{0.9}$	$1.6^{2.0}_{1.2}$	$1.3^{1.6}_{0.9}$	$1.0^{1.2}_{0.7}$	$1.1^{1.5}_{0.8}$	$1.3^{1.6}_{0.9}$	$1.0^{1.3}_{0.8}$

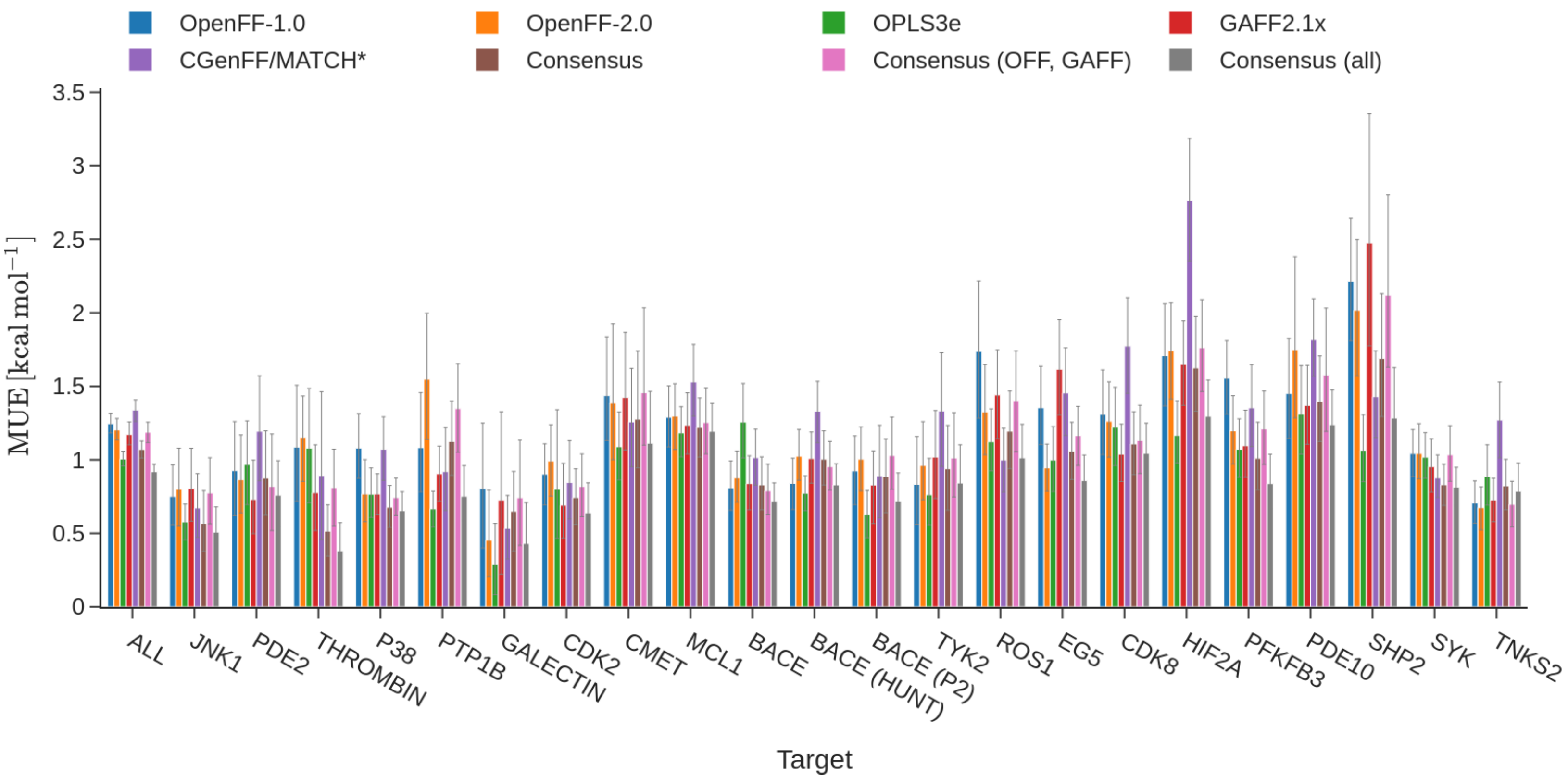

Figure S.2: *Comparison of the five force fields OpenFF-1.0, OpenFF-2.0, GAFF2.1x, CGenFF/MATCH*, OPLS3e and three consensus approaches based on the mean unsigned error (MUE) of the* $\Delta\Delta G$ *values of the perturbations.* Each group represents a target set (or 'all' for all target sets combined) with the MUE values between experimental and calculated value for the respective force fields in different colors. The lower and upper bound of the 95% confidence interval are given as error bars. All values are in kcal mol^{-1}.

Table S.3: *Comparison of the five force fields OpenFF-1.0, OpenFF-2.0, GAFF2.1x, CGenFF/MATCH*, OPLS3e and three consensus approaches based on the mean unsigned error (MUE) of the $\Delta\Delta G$ values of the perturbations.* Each row represents a target set (or 'all' for all target sets combined) with a specified number N of perturbations followed by the MUE between experimental and calculated value for the respective force field. The upper and lower and upper bound of the 95% confidence interval are given as sub- and superscript. All values are in kcal mol^{-1}.

	N	MUE [kcal mol^{-1}]							
		OpenFF -1.0	OpenFF -2.0	CGenFF/ MATCH*	GAFF 2.1x	OPLS 3e	Consensus	Consensus (OFF, GAFF)	Consensus (all)
ALL	1116	$1.2^{1.3}_{1.2}$	$1.2^{1.3}_{1.1}$	$1.3^{1.4}_{1.3}$	$1.2^{1.2}_{1.1}$	$1.0^{1.1}_{1.0}$	$1.1^{1.1}_{1.0}$	$1.2^{1.3}_{1.1}$	$0.9^{1.0}_{0.9}$
BACE	58	$0.8^{1.0}_{0.7}$	$0.9^{1.1}_{0.7}$	$1.0^{1.2}_{0.8}$	$0.8^{1.0}_{0.7}$	$1.3^{1.5}_{1.0}$	$0.8^{1.0}_{0.7}$	$0.7^{0.8}_{0.6}$	$0.7^{0.8}_{0.6}$
BACE (HUNT)	60	$0.9^{1.0}_{0.7}$	$1.0^{1.2}_{0.8}$	$1.3^{1.6}_{1.1}$	$1.0^{1.2}_{0.8}$	$0.8^{0.9}_{0.7}$	$1.0^{1.2}_{0.8}$	$0.9^{1.1}_{0.8}$	$0.8^{1.0}_{0.7}$
BACE (P2)	26	$0.9^{1.2}_{0.7}$	$1.0^{1.2}_{0.8}$	$0.9^{1.2}_{0.6}$	$0.8^{1.1}_{0.6}$	$0.6^{0.8}_{0.5}$	$0.9^{1.2}_{0.7}$	$1.0^{1.3}_{0.8}$	$0.7^{0.9}_{0.5}$
CDK2	25	$0.9^{1.1}_{0.7}$	$1.0^{1.3}_{0.8}$	$0.8^{1.1}_{0.6}$	$0.7^{0.9}_{0.5}$	$0.8^{1.3}_{0.5}$	$0.7^{0.9}_{0.5}$	$0.8^{1.0}_{0.6}$	$0.6^{0.8}_{0.4}$
CDK8	54	$1.3^{1.6}_{1.0}$	$1.3^{1.5}_{1.0}$	$1.8^{2.1}_{1.5}$	$1.1^{1.2}_{0.8}$	$1.3^{1.5}_{1.0}$	$1.1^{1.3}_{0.9}$	$1.1^{1.4}_{0.9}$	$1.0^{1.2}_{0.9}$
CMET	57	$1.4^{1.8}_{1.1}$	$1.3^{1.9}_{1.0}$	$1.2^{1.6}_{1.0}$	$1.4^{1.9}_{1.0}$	$1.1^{1.3}_{0.9}$	$1.2^{1.7}_{1.0}$	$1.4^{1.9}_{1.1}$	$1.1^{1.5}_{0.8}$
EG5	65	$1.4^{1.6}_{1.1}$	$0.9^{1.1}_{0.8}$	$1.4^{1.7}_{1.2}$	$1.5^{1.9}_{1.3}$	$1.0^{1.2}_{0.8}$	$1.0^{1.2}_{0.9}$	$1.2^{1.4}_{1.0}$	$0.9^{1.0}_{0.7}$
GALECTIN	7	$0.7^{1.3}_{0.4}$	$0.4^{0.8}_{0.2}$	$0.5^{0.8}_{0.3}$	$0.6^{1.3}_{0.2}$	$0.2^{0.5}_{0.1}$	$0.7^{0.9}_{0.4}$	$0.7^{1.1}_{0.4}$	$0.4^{0.7}_{0.2}$
HIF2A	80	$1.7^{2.1}_{1.4}$	$1.7^{2.1}_{1.4}$	$2.8^{3.2}_{2.3}$	$1.7^{2.0}_{1.4}$	$1.1^{1.4}_{1.0}$	$1.6^{1.9}_{1.3}$	$1.8^{2.1}_{1.5}$	$1.3^{1.6}_{1.0}$
JNK1	31	$0.8^{1.0}_{0.6}$	$0.7^{1.1}_{0.6}$	$0.7^{0.9}_{0.5}$	$0.7^{1.1}_{0.6}$	$0.6^{0.7}_{0.4}$	$0.6^{0.8}_{0.4}$	$0.7^{1.0}_{0.5}$	$0.5^{0.7}_{0.4}$
MCL1	71	$1.3^{1.5}_{1.1}$	$1.3^{1.5}_{1.1}$	$1.5^{1.8}_{1.3}$	$1.2^{1.5}_{1.0}$	$1.2^{1.4}_{1.0}$	$1.2^{1.4}_{1.0}$	$1.2^{1.5}_{1.0}$	$1.2^{1.4}_{1.0}$
P38	56	$1.0^{1.3}_{0.9}$	$0.7^{1.0}_{0.6}$	$1.1^{1.3}_{0.9}$	$0.8^{0.9}_{0.6}$	$0.8^{0.9}_{0.6}$	$0.7^{0.8}_{0.5}$	$0.7^{0.9}_{0.6}$	$0.7^{0.8}_{0.5}$
PDE10	59	$1.4^{1.8}_{1.1}$	$1.7^{2.4}_{1.3}$	$1.8^{2.1}_{1.6}$	$1.4^{1.7}_{1.1}$	$1.3^{1.6}_{1.0}$	$1.4^{1.7}_{1.1}$	$1.5^{2.1}_{1.2}$	$1.2^{1.5}_{1.0}$
PDE2	34	$1.0^{1.3}_{0.6}$	$0.9^{1.1}_{0.6}$	$1.3^{1.5}_{0.9}$	$0.7^{1.0}_{0.5}$	$0.9^{1.3}_{0.7}$	$0.9^{1.2}_{0.6}$	$0.8^{1.2}_{0.5}$	$0.8^{1.0}_{0.5}$
PFKFB3	66	$1.6^{1.8}_{1.3}$	$1.2^{1.4}_{1.0}$	$1.4^{1.6}_{1.1}$	$1.1^{1.3}_{0.9}$	$1.1^{1.3}_{0.9}$	$1.0^{1.2}_{0.8}$	$1.2^{1.5}_{1.0}$	$0.8^{1.0}_{0.6}$
PTP1B	49	$1.1^{1.4}_{0.8}$	$1.5^{2.0}_{1.1}$	$0.9^{1.2}_{0.7}$	$0.9^{1.1}_{0.7}$	$0.7^{0.8}_{0.5}$	$1.1^{1.4}_{0.9}$	$1.3^{1.6}_{1.1}$	$0.7^{0.9}_{0.6}$
ROS1	61	$1.7^{2.2}_{1.3}$	$1.4^{1.7}_{1.0}$	$1.0^{1.2}_{0.8}$	$1.4^{1.8}_{1.1}$	$1.1^{1.3}_{0.9}$	$1.2^{1.5}_{0.9}$	$1.4^{1.7}_{1.1}$	$1.0^{1.3}_{0.8}$
SHP2	56	$2.2^{2.6}_{1.9}$	$1.9^{2.5}_{1.6}$	$1.4^{1.7}_{1.1}$	$2.3^{3.4}_{1.7}$	$1.1^{1.3}_{0.8}$	$1.7^{2.1}_{1.3}$	$2.1^{2.7}_{1.6}$	$1.3^{1.6}_{1.0}$
SYK	101	$1.1^{1.2}_{0.9}$	$1.1^{1.2}_{0.9}$	$0.9^{1.0}_{0.7}$	$0.9^{1.1}_{0.8}$	$1.0^{1.2}_{0.9}$	$0.8^{1.0}_{0.7}$	$1.0^{1.2}_{0.9}$	$0.8^{0.9}_{0.7}$
THROMBIN	16	$1.1^{1.5}_{0.7}$	$1.2^{1.4}_{0.8}$	$0.7^{1.4}_{0.4}$	$0.8^{1.1}_{0.5}$	$1.2^{1.5}_{0.7}$	$0.5^{0.7}_{0.3}$	$0.8^{1.1}_{0.6}$	$0.3^{0.6}_{0.2}$
TNKS2	60	$0.7^{0.9}_{0.6}$	$0.7^{0.8}_{0.5}$	$1.2^{1.5}_{1.0}$	$0.7^{0.9}_{0.6}$	$0.9^{1.1}_{0.7}$	$0.8^{1.0}_{0.7}$	$0.7^{0.9}_{0.6}$	$0.8^{1.0}_{0.6}$
TYK2	24	$0.9^{1.1}_{0.5}$	$1.0^{1.2}_{0.7}$	$1.4^{1.7}_{1.0}$	$1.0^{1.4}_{0.7}$	$0.7^{1.0}_{0.5}$	$0.9^{1.2}_{0.7}$	$1.0^{1.3}_{0.8}$	$0.9^{1.1}_{0.6}$

Converged edges and force fields of the OpenFF family

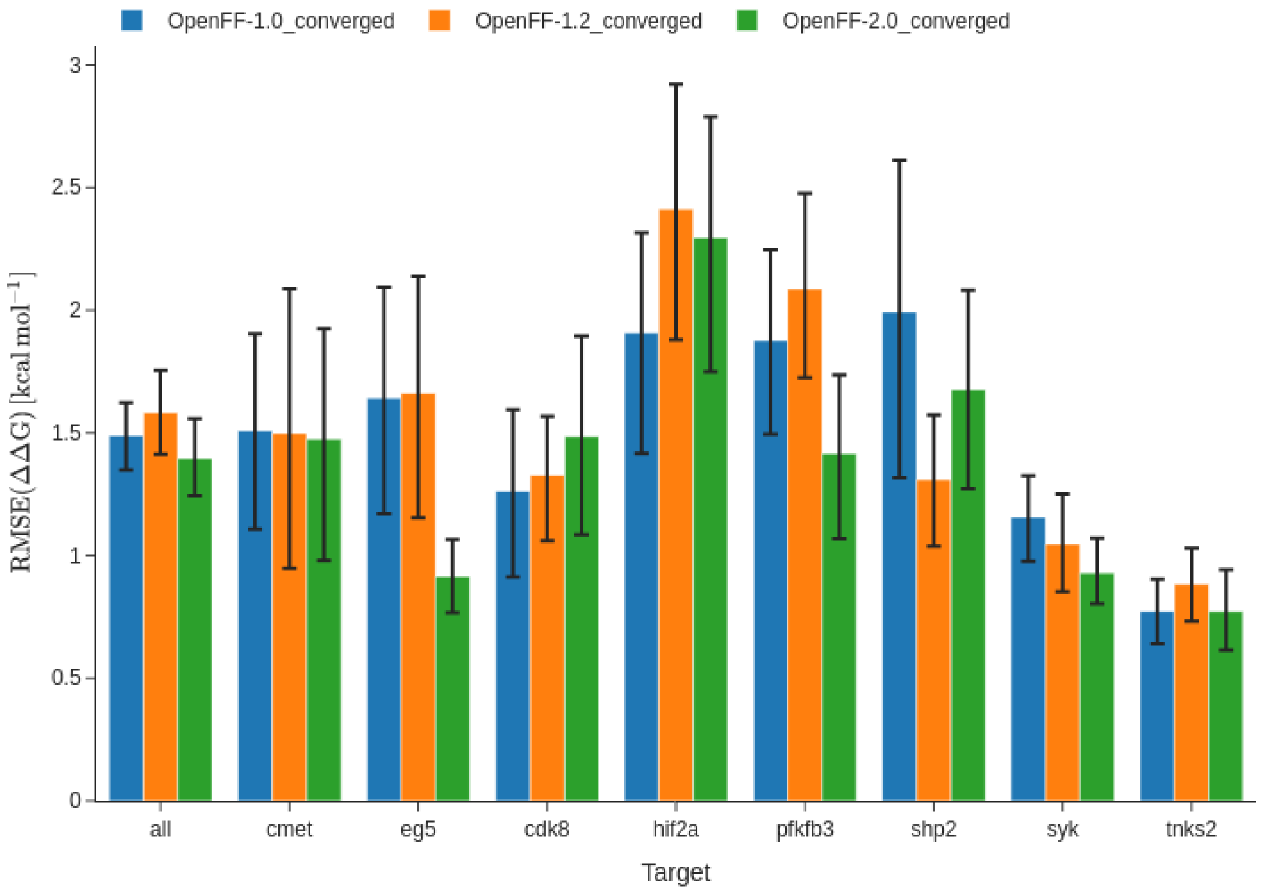

Figure S.3: *Comparison of the three force fields OpenFF-1.0, OpenFF-1.2, and OpenFF-2.0 based on the root mean square error (RMSE) of the $\Delta\Delta G$ values of the perturbations.* Each group represents a target set (or 'all' for all target sets combined) with the RMSE values between experimental and calculated value for the respective force fields in different colors. The lower and upper bound of the 95% confidence interval are given as error bars. All values are in $\mathrm{kcal\,mol^{-1}}$.

Table S.4: *Comparison of the three force fields OpenFF-1.0, OpenFF-1.2, and OpenFF-2.0 based on the root mean square error (RMSE) of the $\Delta\Delta G$ values of the perturbations.* Each row represents a target set (or 'all' for all target sets combined) with a specified number N of perturbations followed by the RMSE between experimental and calculated value for the respective force field. The upper and lower and upper bound of the 95% confidence interval are given as sub- and superscript. All values are in $\mathrm{kcal\,mol^{-1}}$.

	N	RMSE [$\mathrm{kcal\,mol^{-1}}$]		
		OpenFF-1.0	OpenFF-1.2	OpenFF-2.0
ALL	320	$1.5_{1.4}^{1.6}$	$1.5_{1.4}^{1.7}$	$1.4_{1.2}^{1.6}$
CDK8	27	$1.3_{0.9}^{1.6}$	$1.3_{1.1}^{1.6}$	$1.4_{1.1}^{1.9}$
CMET	35	$1.5_{1.1}^{2.0}$	$1.5_{0.9}^{2.1}$	$1.4_{1.0}^{1.9}$
EG5	29	$1.6_{1.2}^{2.1}$	$1.6_{1.2}^{2.1}$	$0.9_{0.8}^{1.1}$
HIF2A	45	$1.8_{1.5}^{2.3}$	$2.4_{1.9}^{2.9}$	$2.3_{1.8}^{2.8}$
PFKFB3	42	$1.9_{1.5}^{2.2}$	$2.0_{1.7}^{2.4}$	$1.4_{1.1}^{1.7}$
SHP2	17	$1.9_{1.4}^{2.5}$	$1.3_{1.0}^{1.6}$	$1.7_{1.3}^{2.1}$
SYK	74	$1.2_{1.0}^{1.3}$	$1.0_{0.9}^{1.2}$	$0.9_{0.8}^{1.1}$
TNKS2	51	$0.8_{0.6}^{0.9}$	$0.9_{0.7}^{1.0}$	$0.8_{0.6}^{1.0}$

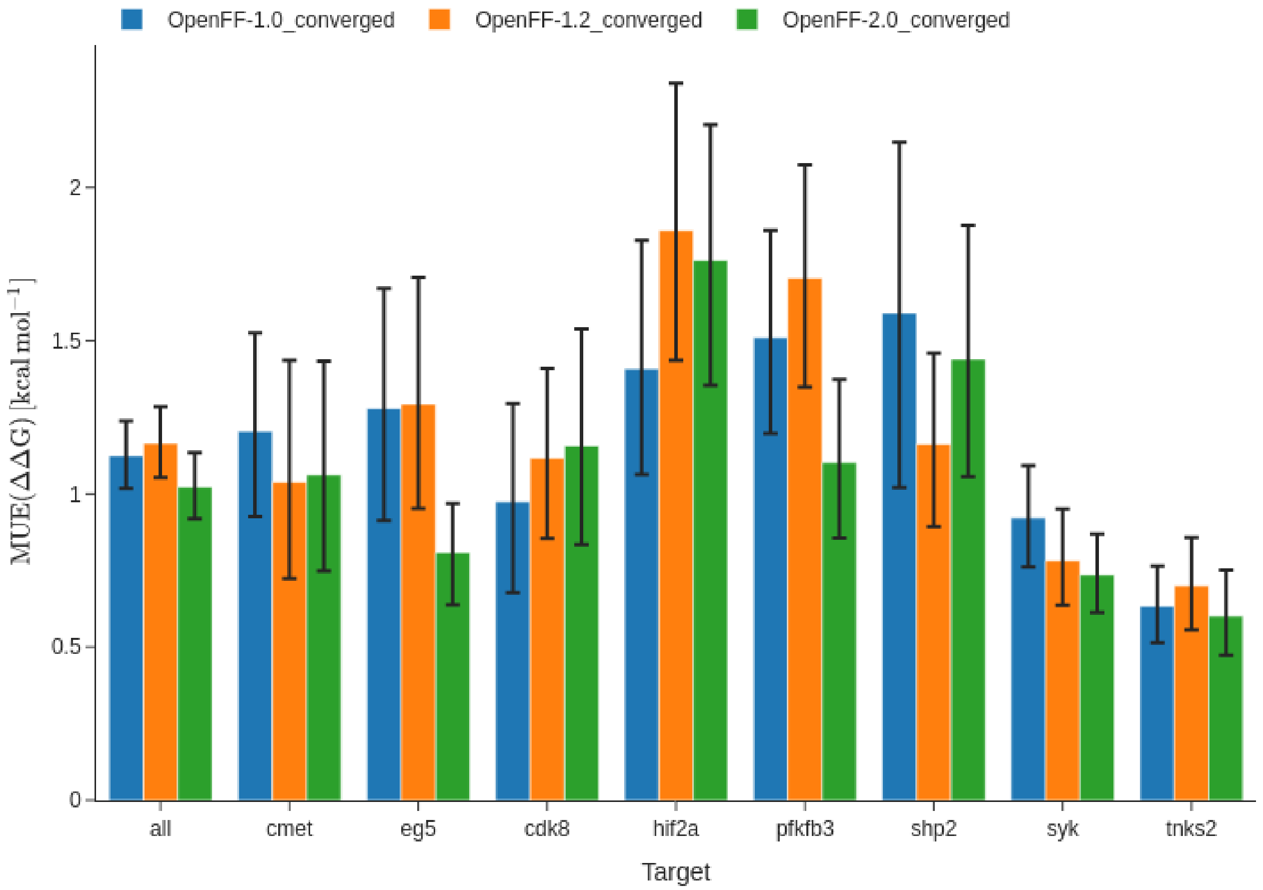

Figure S.4: *Comparison of the three force fields OpenFF-1.0, OpenFF-1.2, and OpenFF-2.0 based on the mean unsigned error (MUE) of the* $\Delta\Delta G$ *values of the perturbations.* Each group represents a target set (or 'all' for all target sets combined) with the MUE values between experimental and calculated value for the respective force fields in different colors. The lower and upper bound of the 95% confidence interval are given as error bars. All values are in kcal mol^{-1}.

Table S.5: *Comparison of the three force fields OpenFF-1.0, OpenFF-1.2, and OpenFF-2.0 based on the mean unsigned error (MUE) of the* $\Delta\Delta G$ *values of the perturbations.* Each row represents a target set (or 'all' for all target sets combined) with a specified number N of perturbations followed by the MUE between experimental and calculated value for the respective force field. The upper and lower and upper bound of the 95% confidence interval are given as sub- and superscript. All values are in kcal mol^{-1}.

	N	MUE [kcal mol^{-1}]		
		OpenFF-1.0	OpenFF-1.2	OpenFF-2.0
ALL	320	$1.1_{1.0}^{1.2}$	$1.1_{1.0}^{1.3}$	$1.0_{0.9}^{1.1}$
CDK8	27	$1.0_{0.7}^{1.3}$	$1.1_{0.9}^{1.4}$	$1.1_{0.8}^{1.5}$
CMET	35	$1.3_{0.9}^{1.5}$	$1.0_{0.7}^{1.5}$	$1.0_{0.7}^{1.4}$
EG5	29	$1.3_{0.9}^{1.7}$	$1.3_{0.9}^{1.7}$	$0.8_{0.7}^{1.0}$
HIF2A	45	$1.4_{1.0}^{1.8}$	$1.9_{1.4}^{2.3}$	$1.7_{1.3}^{2.2}$
PFKFB3	42	$1.5_{1.2}^{1.8}$	$1.7_{1.3}^{2.1}$	$1.1_{0.8}^{1.4}$
SHP2	17	$1.6_{1.0}^{2.2}$	$1.2_{0.9}^{1.4}$	$1.4_{1.0}^{1.9}$
SYK	74	$0.9_{0.8}^{1.1}$	$0.8_{0.6}^{0.9}$	$0.7_{0.6}^{0.9}$
TNKS2	51	$0.6_{0.5}^{0.8}$	$0.7_{0.6}^{0.9}$	$0.6_{0.5}^{0.7}$

Performance of force fields based on ΔG

Aggregated statistics

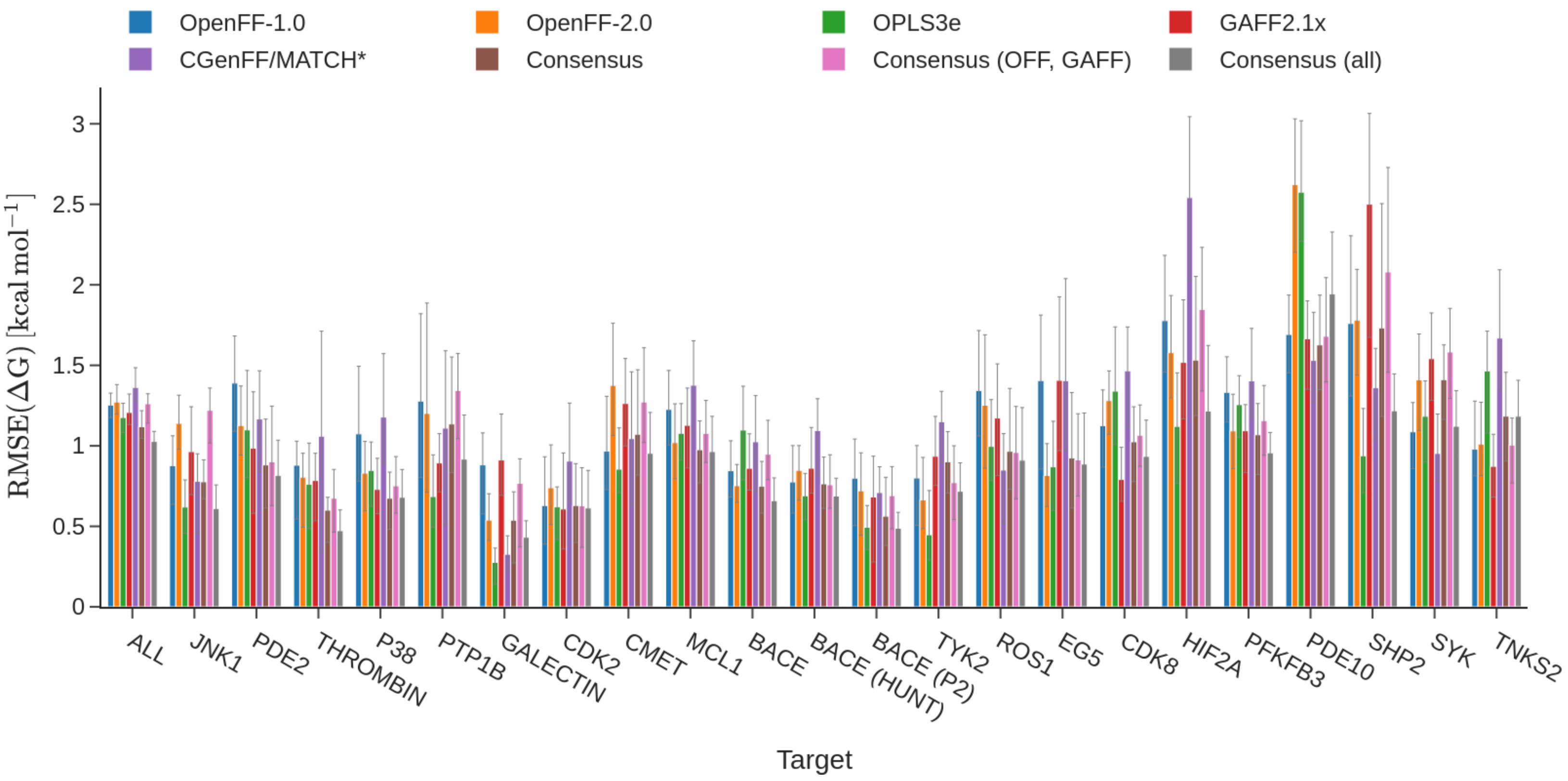

Figure S.5: *Comparison of the five force fields OpenFF-1.0, OpenFF-2.0, GAFF2.1x, CGenFF/MATCH*, OPLS3e and three consensus approaches based on the root mean squared error (RMSE) of the ΔG values of the ligands.* Each group represents a target set (or 'all' for all target sets combined) with the RMSE values between experimental and calculated value for the respective force fields in different colors. The lower and upper bound of the 95% confidence interval are given as error bars. All values are in kcal mol^{-1}.

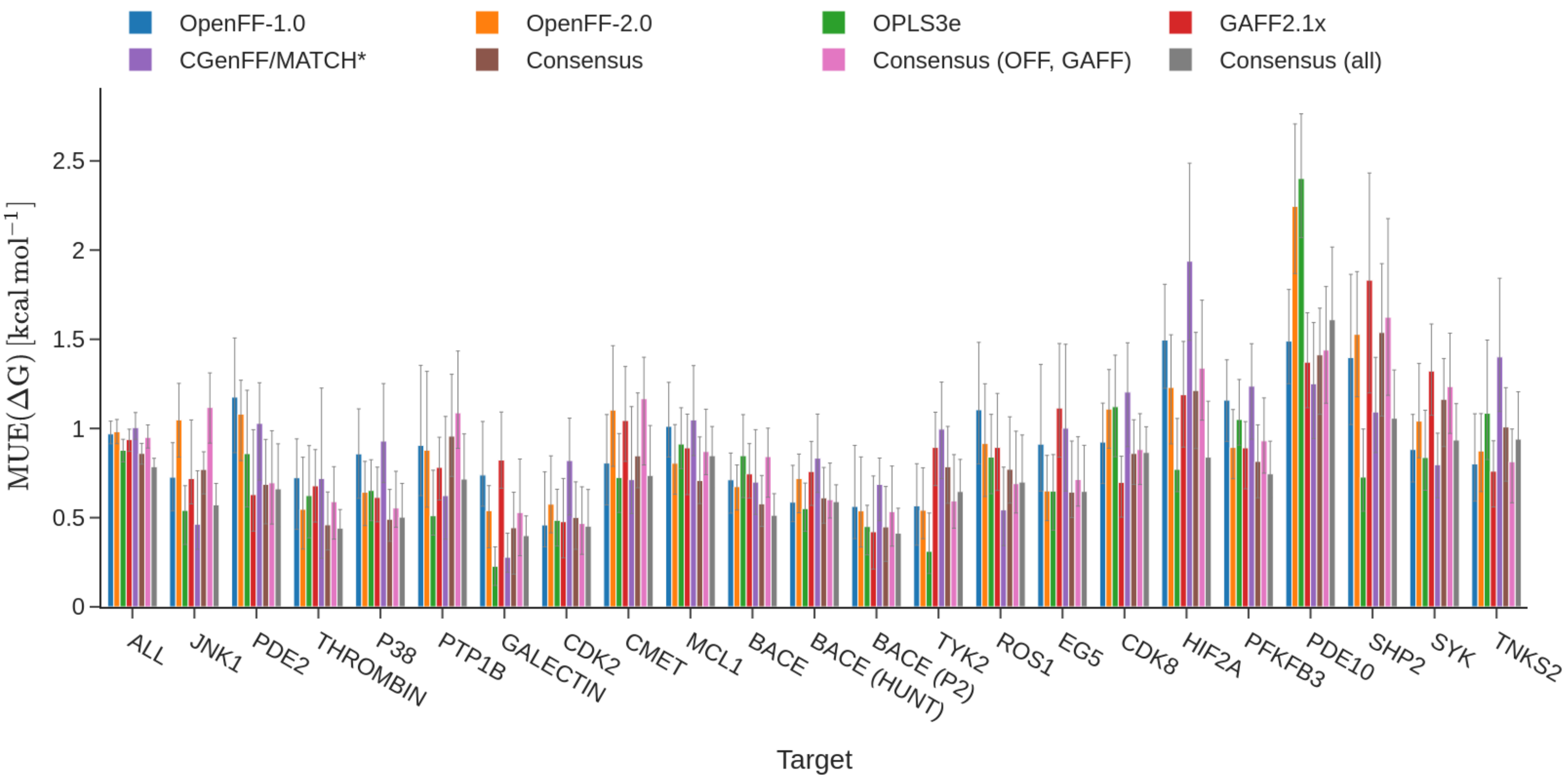

Figure S.6: *Comparison of the five force fields OpenFF-1.0, OpenFF-2.0, GAFF2.1x, CGenFF/MATCH*, OPLS3e and three consensus approaches based on the mean unsigned error (MUE) of the* ΔG *values of the ligands.* Each group represents a target set (or 'all' for all target sets combined) with the MUE values between experimental and calculated value for the respective force fields in different colors. The lower and upper bound of the 95% confidence interval are given as error bars. All values are in $\mathrm{kcal\,mol^{-1}}$.

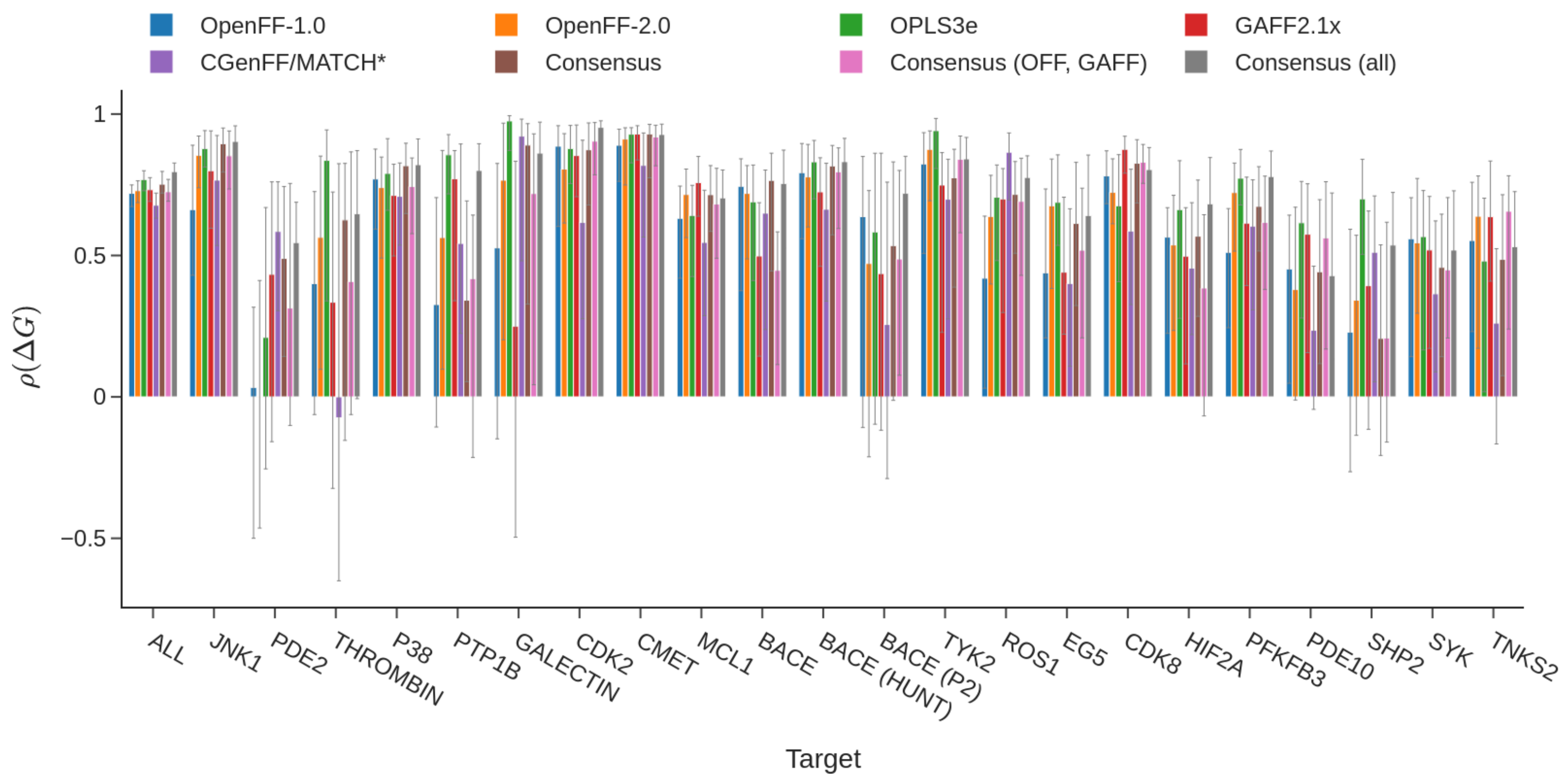

Figure S.7: *Comparison of the five force fields OpenFF-1.0, OpenFF-2.0, GAFF2.1x, CGenFF/MATCH*, OPLS3e and three consensus approaches based on the Spearman's ρ of the ΔG values of the ligands.* Each group represents a target set (or 'all' for all target sets combined) with the Spearman's ρ values between experimental and calculated value for the respective force fields in different colors. The lower and upper bound of the 95% confidence interval are given as error bars.

Table S.6: *Comparison of the five force fields OpenFF-1.0, OpenFF-2.0, GAFF2.1x, CGenFF/MATCH*, OPLS3e and three consensus approaches based on the root mean squared error (RMSE) of the* ΔG *values of the ligands.* Each row represents a target set (or 'all' for all target sets combined) with a specified number N of perturbations followed by the RMSE between experimental and calculated value for the respective force field. The upper and lower and upper bound of the 95% confidence interval are given as sub- and superscript. All values are in kcal mol^{-1}.

	N	RMSE [kcal mol^{-1}]							
		OpenFF -1.0	OpenFF -2.0	CGenFF/ MATCH*	GAFF 2.1x	OPLS 3e	Consensus	Consensus (OFF, GAFF)	Consensus (all)
ALL	598	$1.3^{1.2}_{1.3}$	$1.3^{1.2}_{1.4}$	$1.4^{1.3}_{1.5}$	$1.2^{1.1}_{1.3}$	$1.2^{1.1}_{1.3}$	$1.1^{1.0}_{1.2}$	$1.2^{1.2}_{1.3}$	$1.0^{0.9}_{1.1}$
BACE	36	$0.9^{0.7}_{1.0}$	$0.8^{0.7}_{0.9}$	$1.0^{0.7}_{1.3}$	$0.9^{0.7}_{1.1}$	$1.0^{0.8}_{1.3}$	$0.8^{0.6}_{0.9}$	$1.0^{0.8}_{1.2}$	$0.7^{0.5}_{0.8}$
BACE (HUNT)	32	$0.8^{0.6}_{1.0}$	$0.8^{0.7}_{1.0}$	$1.1^{0.8}_{1.3}$	$0.9^{0.7}_{1.1}$	$0.7^{0.5}_{0.8}$	$0.8^{0.6}_{0.9}$	$0.8^{0.6}_{0.9}$	$0.7^{0.5}_{0.8}$
BACE (P2)	12	$0.8^{0.5}_{1.1}$	$0.7^{0.5}_{1.0}$	$0.7^{0.6}_{0.9}$	$0.7^{0.3}_{0.9}$	$0.5^{0.4}_{0.6}$	$0.6^{0.4}_{0.8}$	$0.7^{0.5}_{0.9}$	$0.5^{0.4}_{0.6}$
CDK2	16	$0.6^{0.4}_{0.9}$	$0.7^{0.5}_{1.0}$	$1.0^{0.7}_{1.3}$	$0.7^{0.4}_{1.0}$	$0.6^{0.4}_{0.7}$	$0.6^{0.4}_{0.9}$	$0.6^{0.4}_{0.9}$	$0.6^{0.3}_{0.9}$
CDK8	33	$1.1^{0.9}_{1.3}$	$1.2^{1.1}_{1.5}$	$1.5^{1.1}_{1.7}$	$0.8^{0.7}_{1.0}$	$1.3^{1.0}_{1.8}$	$1.0^{0.8}_{1.2}$	$1.0^{0.9}_{1.2}$	$0.9^{0.8}_{1.2}$
CMET	24	$1.0^{0.7}_{1.3}$	$1.4^{1.1}_{1.7}$	$1.0^{0.6}_{1.5}$	$1.2^{1.0}_{1.5}$	$0.9^{0.7}_{1.1}$	$1.2^{0.8}_{1.5}$	$1.3^{1.0}_{1.6}$	$0.9^{0.7}_{1.2}$
EG5	28	$1.4^{0.8}_{1.8}$	$0.8^{0.6}_{1.0}$	$1.5^{1.0}_{2.0}$	$1.4^{1.0}_{1.9}$	$0.8^{0.6}_{1.2}$	$0.9^{0.6}_{1.3}$	$0.9^{0.7}_{1.2}$	$0.9^{0.6}_{1.2}$
GALECTIN	8	$0.9^{0.6}_{1.1}$	$0.6^{0.4}_{0.7}$	$0.3^{0.2}_{0.4}$	$0.9^{0.7}_{1.2}$	$0.3^{0.1}_{0.4}$	$0.5^{0.3}_{0.7}$	$0.6^{0.4}_{0.9}$	$0.4^{0.3}_{0.5}$
HIF2A	42	$1.8^{1.5}_{2.1}$	$1.6^{1.3}_{1.9}$	$2.6^{2.1}_{3.1}$	$1.5^{1.2}_{1.9}$	$1.2^{0.8}_{1.5}$	$1.7^{1.2}_{2.0}$	$1.8^{1.3}_{2.3}$	$1.3^{0.8}_{1.6}$
JNK1	21	$0.9^{0.6}_{1.1}$	$1.2^{1.0}_{1.3}$	$0.8^{0.4}_{1.0}$	$0.9^{0.7}_{1.2}$	$0.7^{0.5}_{0.8}$	$0.8^{0.7}_{0.9}$	$1.2^{1.0}_{1.4}$	$0.6^{0.5}_{0.8}$
MCL1	42	$1.2^{1.0}_{1.5}$	$1.0^{0.8}_{1.3}$	$1.4^{1.1}_{1.6}$	$1.1^{0.9}_{1.4}$	$1.1^{0.9}_{1.3}$	$1.0^{0.8}_{1.2}$	$1.1^{0.9}_{1.3}$	$1.0^{0.8}_{1.2}$
P38	34	$1.1^{0.8}_{1.5}$	$0.8^{0.6}_{1.0}$	$1.2^{0.9}_{1.6}$	$0.8^{0.6}_{0.9}$	$0.8^{0.6}_{1.0}$	$0.7^{0.5}_{0.9}$	$0.8^{0.6}_{0.9}$	$0.7^{0.5}_{0.9}$
PDE10	34	$1.6^{1.4}_{2.0}$	$2.6^{2.2}_{3.0}$	$1.6^{1.3}_{1.8}$	$1.6^{1.3}_{1.9}$	$2.7^{2.3}_{3.0}$	$1.7^{1.3}_{1.9}$	$1.7^{1.4}_{2.1}$	$1.9^{1.6}_{2.3}$
PDE2	21	$1.4^{1.1}_{1.7}$	$1.2^{1.0}_{1.4}$	$1.2^{0.9}_{1.5}$	$0.9^{0.6}_{1.4}$	$1.2^{0.8}_{1.5}$	$0.8^{0.6}_{1.2}$	$0.8^{0.6}_{1.2}$	$0.9^{0.6}_{1.0}$
PFKFB3	40	$1.4^{1.1}_{1.6}$	$1.1^{0.9}_{1.3}$	$1.5^{1.2}_{1.7}$	$1.1^{0.8}_{1.3}$	$1.2^{1.1}_{1.4}$	$1.1^{0.8}_{1.3}$	$1.2^{0.9}_{1.4}$	$1.0^{0.7}_{1.1}$
PTP1B	23	$1.3^{0.8}_{1.8}$	$1.4^{0.7}_{1.9}$	$1.1^{0.5}_{1.6}$	$0.9^{0.7}_{1.1}$	$0.7^{0.5}_{0.9}$	$1.2^{0.8}_{1.6}$	$1.4^{1.1}_{1.6}$	$0.8^{0.6}_{1.2}$
ROS1	28	$1.4^{1.1}_{1.8}$	$1.2^{0.8}_{1.6}$	$0.8^{0.5}_{1.1}$	$1.1^{0.8}_{1.5}$	$1.0^{0.8}_{1.3}$	$1.0^{0.7}_{1.3}$	$1.0^{0.7}_{1.2}$	$0.9^{0.6}_{1.2}$
SHP2	26	$1.7^{1.3}_{2.3}$	$1.8^{1.4}_{2.1}$	$1.3^{1.1}_{1.6}$	$2.4^{1.7}_{3.1}$	$0.9^{0.7}_{1.2}$	$1.9^{1.3}_{2.5}$	$2.1^{1.4}_{2.7}$	$1.2^{1.0}_{1.5}$
SYK	44	$1.1^{0.9}_{1.2}$	$1.4^{1.1}_{1.7}$	$1.0^{0.8}_{1.2}$	$1.5^{1.3}_{1.8}$	$1.1^{0.9}_{1.4}$	$1.4^{1.2}_{1.7}$	$1.6^{1.3}_{1.9}$	$1.1^{0.9}_{1.3}$
THROMBIN	11	$0.8^{0.5}_{1.0}$	$0.7^{0.5}_{1.0}$	$1.0^{0.3}_{1.7}$	$0.7^{0.5}_{1.0}$	$0.8^{0.5}_{1.0}$	$0.6^{0.4}_{0.7}$	$0.7^{0.5}_{0.9}$	$0.5^{0.4}_{0.6}$
TNKS2	27	$1.0^{0.8}_{1.3}$	$1.1^{0.8}_{1.2}$	$1.7^{1.3}_{2.1}$	$0.9^{0.7}_{1.1}$	$1.4^{1.1}_{1.7}$	$1.2^{0.9}_{1.4}$	$0.9^{0.8}_{1.2}$	$1.2^{0.9}_{1.4}$
TYK2	16	$0.8^{0.5}_{1.0}$	$0.7^{0.5}_{0.9}$	$1.2^{0.9}_{1.3}$	$0.9^{0.8}_{1.2}$	$0.5^{0.3}_{0.7}$	$0.9^{0.7}_{1.1}$	$0.7^{0.6}_{1.0}$	$0.7^{0.6}_{0.9}$

Table S.7: *Comparison of the five force fields OpenFF-1.0, OpenFF-2.0, GAFF2.1x, CGenFF/MATCH*, OPLS3e and three consensus approaches based on the mean unsigned error (MUE) of the* ΔG *values of the perturbations.* Each row represents a target set (or 'all' for all target sets combined) with a specified number N of perturbations followed by the MUE between experimental and calculated value for the respective force field. The upper and lower and upper bound of the 95% confidence interval are given as sub- and superscript. All values are in $\mathrm{kcal\,mol^{-1}}$.

	N	MUE [$\mathrm{kcal\,mol^{-1}}$]							
		OpenFF -1.0	OpenFF -2.0	CGenFF/ MATCH*	GAFF 2.1x	OPLS 3e	Consensus	Consensus (OFF, GAFF)	Consensus (all)
ALL	598	$1.0^{0.9}_{1.0}$	$1.0^{0.9}_{1.0}$	$1.0^{0.9}_{1.1}$	$0.9^{0.9}_{1.0}$	$0.9^{0.8}_{0.9}$	$0.9^{0.8}_{0.9}$	$1.0^{0.9}_{1.0}$	$0.8^{0.7}_{0.8}$
BACE	36	$0.7^{0.5}_{0.9}$	$0.6^{0.5}_{0.8}$	$0.7^{0.5}_{1.0}$	$0.8^{0.6}_{0.9}$	$0.8^{0.6}_{1.0}$	$0.6^{0.4}_{0.7}$	$0.8^{0.6}_{1.0}$	$0.5^{0.4}_{0.6}$
BACE (HUNT)	32	$0.6^{0.5}_{0.8}$	$0.7^{0.5}_{0.9}$	$0.9^{0.6}_{1.1}$	$0.7^{0.6}_{0.9}$	$0.6^{0.4}_{0.7}$	$0.6^{0.5}_{0.8}$	$0.6^{0.5}_{0.8}$	$0.5^{0.4}_{0.7}$
BACE (P2)	12	$0.6^{0.4}_{0.9}$	$0.6^{0.3}_{0.8}$	$0.6^{0.5}_{0.8}$	$0.4^{0.2}_{0.7}$	$0.4^{0.3}_{0.6}$	$0.4^{0.2}_{0.7}$	$0.6^{0.3}_{0.8}$	$0.4^{0.3}_{0.6}$
CDK2	16	$0.5^{0.3}_{0.8}$	$0.6^{0.4}_{0.9}$	$0.7^{0.5}_{1.0}$	$0.5^{0.3}_{0.7}$	$0.5^{0.3}_{0.6}$	$0.5^{0.3}_{0.7}$	$0.5^{0.3}_{0.7}$	$0.4^{0.3}_{0.7}$
CDK8	33	$0.9^{0.7}_{1.1}$	$1.1^{0.9}_{1.3}$	$1.1^{0.9}_{1.5}$	$0.7^{0.5}_{0.8}$	$1.1^{0.9}_{1.4}$	$0.8^{0.7}_{1.0}$	$0.9^{0.7}_{1.1}$	$0.8^{0.7}_{1.0}$
CMET	24	$0.8^{0.6}_{1.1}$	$1.2^{0.8}_{1.5}$	$0.7^{0.5}_{1.1}$	$1.0^{0.8}_{1.4}$	$0.7^{0.6}_{1.0}$	$0.8^{0.7}_{1.2}$	$1.2^{0.8}_{1.4}$	$0.7^{0.6}_{1.0}$
EG5	28	$0.9^{0.6}_{1.3}$	$0.6^{0.5}_{0.9}$	$1.0^{0.7}_{1.5}$	$1.1^{0.8}_{1.5}$	$0.6^{0.5}_{0.9}$	$0.7^{0.5}_{0.9}$	$0.8^{0.6}_{1.0}$	$0.7^{0.5}_{0.9}$
GALECTIN	8	$0.8^{0.6}_{1.0}$	$0.5^{0.3}_{0.7}$	$0.3^{0.2}_{0.4}$	$0.8^{0.7}_{1.1}$	$0.2^{0.1}_{0.3}$	$0.4^{0.2}_{0.6}$	$0.5^{0.3}_{0.9}$	$0.4^{0.3}_{0.5}$
HIF2A	42	$1.5^{1.2}_{1.8}$	$1.2^{0.9}_{1.5}$	$2.0^{1.5}_{2.5}$	$1.2^{0.9}_{1.5}$	$0.9^{0.6}_{1.1}$	$1.1^{0.9}_{1.5}$	$1.4^{1.1}_{1.7}$	$0.9^{0.6}_{1.2}$
JNK1	21	$0.7^{0.5}_{0.9}$	$1.1^{0.8}_{1.2}$	$0.5^{0.3}_{0.7}$	$0.8^{0.6}_{1.1}$	$0.5^{0.3}_{0.7}$	$0.7^{0.6}_{0.9}$	$1.1^{0.9}_{1.3}$	$0.6^{0.5}_{0.7}$
MCL1	42	$1.0^{0.8}_{1.3}$	$0.9^{0.6}_{1.0}$	$1.1^{0.9}_{1.3}$	$0.8^{0.7}_{1.1}$	$0.9^{0.8}_{1.1}$	$0.7^{0.6}_{0.9}$	$1.0^{0.7}_{1.1}$	$0.9^{0.7}_{1.0}$
P38	34	$0.9^{0.6}_{1.1}$	$0.6^{0.5}_{0.8}$	$0.9^{0.7}_{1.2}$	$0.6^{0.5}_{0.8}$	$0.6^{0.5}_{0.8}$	$0.5^{0.4}_{0.7}$	$0.6^{0.4}_{0.8}$	$0.5^{0.4}_{0.7}$
PDE10	34	$1.5^{1.2}_{1.8}$	$2.3^{1.9}_{2.7}$	$1.3^{1.0}_{1.6}$	$1.4^{1.1}_{1.7}$	$2.4^{2.0}_{2.8}$	$1.4^{1.1}_{1.7}$	$1.5^{1.1}_{1.8}$	$1.6^{1.4}_{2.0}$
PDE2	21	$1.1^{0.9}_{1.5}$	$1.1^{0.8}_{1.3}$	$1.0^{0.7}_{1.3}$	$0.6^{0.4}_{1.0}$	$0.9^{0.6}_{1.2}$	$0.7^{0.5}_{0.9}$	$0.7^{0.5}_{1.0}$	$0.7^{0.4}_{0.9}$
PFKFB3	40	$1.1^{0.9}_{1.4}$	$0.9^{0.7}_{1.1}$	$1.2^{0.9}_{1.5}$	$0.9^{0.7}_{1.1}$	$1.1^{0.9}_{1.3}$	$0.8^{0.6}_{1.0}$	$1.0^{0.8}_{1.2}$	$0.8^{0.6}_{0.9}$
PTP1B	23	$0.9^{0.6}_{1.4}$	$0.8^{0.6}_{1.3}$	$0.6^{0.4}_{1.1}$	$0.8^{0.6}_{1.0}$	$0.5^{0.4}_{0.8}$	$1.0^{0.8}_{1.3}$	$1.2^{0.9}_{1.4}$	$0.7^{0.5}_{1.0}$
ROS1	28	$1.1^{0.8}_{1.4}$	$0.9^{0.6}_{1.3}$	$0.5^{0.3}_{0.8}$	$0.9^{0.7}_{1.2}$	$0.8^{0.6}_{1.1}$	$0.8^{0.6}_{1.1}$	$0.7^{0.5}_{1.0}$	$0.7^{0.5}_{0.9}$
SHP2	26	$1.4^{1.0}_{1.9}$	$1.5^{1.2}_{1.9}$	$1.1^{0.8}_{1.4}$	$1.8^{1.2}_{2.5}$	$0.7^{0.5}_{1.0}$	$1.3^{1.1}_{1.9}$	$1.6^{1.2}_{2.1}$	$1.1^{0.8}_{1.3}$
SYK	44	$0.9^{0.7}_{1.1}$	$1.0^{0.8}_{1.4}$	$0.7^{0.6}_{1.0}$	$1.3^{1.1}_{1.6}$	$0.8^{0.7}_{1.1}$	$1.1^{0.9}_{1.4}$	$1.2^{1.0}_{1.5}$	$0.9^{0.8}_{1.1}$
THROMBIN	11	$0.6^{0.4}_{0.9}$	$0.6^{0.3}_{0.9}$	$0.7^{0.3}_{1.2}$	$0.7^{0.5}_{0.9}$	$0.6^{0.4}_{0.9}$	$0.4^{0.3}_{0.6}$	$0.6^{0.4}_{0.8}$	$0.4^{0.3}_{0.6}$
TNKS2	27	$0.8^{0.6}_{1.1}$	$0.9^{0.6}_{1.1}$	$1.5^{1.1}_{1.8}$	$0.7^{0.6}_{0.9}$	$1.1^{0.8}_{1.5}$	$0.9^{0.7}_{1.2}$	$0.8^{0.6}_{1.0}$	$1.0^{0.7}_{1.2}$
TYK2	16	$0.6^{0.4}_{0.8}$	$0.6^{0.4}_{0.8}$	$1.1^{0.7}_{1.3}$	$0.9^{0.7}_{1.1}$	$0.3^{0.2}_{0.5}$	$0.8^{0.6}_{1.0}$	$0.7^{0.4}_{0.8}$	$0.6^{0.5}_{0.8}$

Table S.8: *Comparison of the five force fields OpenFF-1.0, OpenFF-2.0, GAFF2.1x, CGenFF/MATCH*, OPLS3e and three consensus approaches based on the Spearman's ρ of the ΔG values of the ligands.* Each row represents a target set (or 'all' for all target sets combined) with a specified number N of perturbations followed by the ρ between experimental and calculated value for the respective force field. The upper and lower and upper bound of the 95% confidence interval are given as sub- and superscript.

	N	ρ							
		OpenFF -1.0	OpenFF -2.0	CGenFF/ MATCH*	GAFF 2.1x	OPLS 3e	Consensus	Consensus (OFF, GAFF)	Consensus (all)
ALL	598	$0.7^{0.7}_{0.7}$	$0.7^{0.7}_{0.8}$	$0.7^{0.6}_{0.7}$	$0.7^{0.7}_{0.8}$	$0.8^{0.7}_{0.8}$	$0.8^{0.7}_{0.8}$	$0.7^{0.7}_{0.8}$	$0.8^{0.8}_{0.8}$
BACE	36	$0.7^{0.4}_{0.8}$	$0.7^{0.5}_{0.8}$	$0.6^{0.2}_{0.8}$	$0.5^{0.1}_{0.7}$	$0.7^{0.4}_{0.8}$	$0.7^{0.4}_{0.9}$	$0.4^{0.1}_{0.6}$	$0.8^{0.5}_{0.9}$
BACE (HUNT)	32	$0.8^{0.5}_{0.9}$	$0.8^{0.6}_{0.9}$	$0.7^{0.3}_{0.8}$	$0.7^{0.5}_{0.8}$	$0.8^{0.7}_{0.9}$	$0.8^{0.6}_{0.9}$	$0.8^{0.6}_{0.9}$	$0.8^{0.7}_{0.9}$
BACE (P2)	12	$0.6^{-0.1}_{0.9}$	$0.4^{-0.2}_{0.7}$	$0.3^{-0.3}_{0.7}$	$0.5^{-0.1}_{0.9}$	$0.6^{-0.1}_{0.9}$	$0.5^{-0.1}_{0.8}$	$0.5^{0.1}_{0.8}$	$0.7^{0.2}_{0.9}$
CDK2	16	$0.9^{0.6}_{1.0}$	$0.8^{0.6}_{0.9}$	$0.6^{0.3}_{0.9}$	$0.9^{0.7}_{1.0}$	$0.9^{0.8}_{1.0}$	$0.9^{0.7}_{1.0}$	$0.9^{0.8}_{1.0}$	$1.0^{0.7}_{1.0}$
CDK8	33	$0.8^{0.7}_{0.9}$	$0.8^{0.6}_{0.8}$	$0.7^{0.4}_{0.8}$	$0.9^{0.8}_{0.9}$	$0.7^{0.4}_{0.9}$	$0.9^{0.7}_{0.9}$	$0.8^{0.7}_{0.9}$	$0.8^{0.7}_{0.9}$
CMET	24	$0.9^{0.8}_{0.9}$	$0.9^{0.7}_{1.0}$	$0.9^{0.5}_{0.9}$	$0.9^{0.8}_{1.0}$	$0.9^{0.8}_{0.9}$	$0.9^{0.8}_{1.0}$	$0.9^{0.8}_{1.0}$	$0.9^{0.8}_{1.0}$
EG5	28	$0.4^{0.2}_{0.7}$	$0.6^{0.4}_{0.8}$	$0.5^{0.1}_{0.7}$	$0.4^{0.2}_{0.7}$	$0.7^{0.5}_{0.9}$	$0.6^{0.3}_{0.8}$	$0.5^{0.2}_{0.7}$	$0.7^{0.4}_{0.9}$
GALECTIN	8	$0.5^{-0.2}_{0.8}$	$0.8^{0.2}_{0.9}$	$0.9^{0.5}_{1.0}$	$0.3^{-0.5}_{0.8}$	$1.0^{0.8}_{1.0}$	$0.8^{0.3}_{1.0}$	$0.6^{0.0}_{0.9}$	$0.9^{0.5}_{1.0}$
HIF2A	42	$0.5^{0.2}_{0.7}$	$0.5^{0.2}_{0.7}$	$0.5^{0.3}_{0.7}$	$0.4^{0.1}_{0.7}$	$0.7^{0.3}_{0.8}$	$0.6^{0.3}_{0.8}$	$0.4^{-0.0}_{0.7}$	$0.7^{0.3}_{0.8}$
JNK1	21	$0.7^{0.4}_{0.9}$	$0.9^{0.7}_{0.9}$	$0.8^{0.5}_{0.9}$	$0.8^{0.6}_{0.9}$	$0.9^{0.8}_{0.9}$	$0.9^{0.8}_{1.0}$	$0.9^{0.7}_{0.9}$	$0.9^{0.8}_{1.0}$
MCL1	42	$0.6^{0.4}_{0.7}$	$0.7^{0.5}_{0.8}$	$0.6^{0.3}_{0.7}$	$0.8^{0.7}_{0.9}$	$0.6^{0.4}_{0.7}$	$0.7^{0.6}_{0.8}$	$0.7^{0.5}_{0.8}$	$0.7^{0.5}_{0.8}$
P38	34	$0.7^{0.6}_{0.9}$	$0.7^{0.5}_{0.8}$	$0.7^{0.5}_{0.8}$	$0.7^{0.5}_{0.8}$	$0.8^{0.6}_{0.9}$	$0.8^{0.6}_{0.9}$	$0.7^{0.6}_{0.8}$	$0.8^{0.7}_{0.9}$
PDE10	34	$0.3^{0.0}_{0.6}$	$0.4^{-0.0}_{0.7}$	$0.3^{-0.0}_{0.5}$	$0.6^{0.2}_{0.8}$	$0.6^{0.3}_{0.7}$	$0.5^{0.1}_{0.7}$	$0.6^{0.2}_{0.7}$	$0.6^{0.1}_{0.7}$
PDE2	21	$-0.1^{-0.5}_{0.3}$	$0.0^{-0.5}_{0.4}$	$0.5^{0.3}_{0.8}$	$0.3^{-0.2}_{0.8}$	$0.3^{-0.2}_{0.6}$	$0.4^{0.1}_{0.8}$	$0.4^{-0.1}_{0.7}$	$0.5^{0.2}_{0.7}$
PFKFB3	40	$0.5^{0.2}_{0.7}$	$0.7^{0.5}_{0.8}$	$0.6^{0.3}_{0.8}$	$0.7^{0.4}_{0.8}$	$0.8^{0.7}_{0.9}$	$0.7^{0.5}_{0.8}$	$0.7^{0.4}_{0.8}$	$0.8^{0.6}_{0.9}$
PTP1B	23	$0.3^{-0.1}_{0.7}$	$0.6^{0.1}_{0.9}$	$0.6^{0.2}_{0.9}$	$0.7^{0.3}_{0.9}$	$0.9^{0.7}_{0.9}$	$0.5^{0.0}_{0.7}$	$0.4^{-0.2}_{0.6}$	$0.8^{0.5}_{0.9}$
ROS1	28	$0.4^{0.0}_{0.6}$	$0.7^{0.4}_{0.8}$	$0.9^{0.7}_{0.9}$	$0.6^{0.3}_{0.8}$	$0.7^{0.5}_{0.8}$	$0.7^{0.5}_{0.8}$	$0.7^{0.4}_{0.8}$	$0.8^{0.6}_{0.8}$
SHP2	26	$0.3^{-0.2}_{0.6}$	$0.3^{-0.1}_{0.6}$	$0.5^{0.1}_{0.7}$	$0.4^{-0.1}_{0.7}$	$0.7^{0.5}_{0.8}$	$0.1^{-0.2}_{0.5}$	$0.3^{-0.2}_{0.6}$	$0.5^{0.2}_{0.7}$
SYK	44	$0.5^{0.2}_{0.7}$	$0.6^{0.3}_{0.8}$	$0.4^{0.1}_{0.6}$	$0.5^{0.2}_{0.7}$	$0.6^{0.1}_{0.7}$	$0.5^{0.1}_{0.6}$	$0.6^{0.2}_{0.7}$	$0.5^{0.2}_{0.7}$
THROMBIN	11	$0.4^{-0.1}_{0.7}$	$0.6^{0.1}_{0.8}$	$-0.2^{-0.7}_{0.8}$	$0.2^{-0.3}_{0.7}$	$0.8^{0.4}_{0.9}$	$0.5^{-0.2}_{0.8}$	$0.4^{-0.1}_{0.8}$	$0.6^{-0.0}_{0.9}$
TNKS2	27	$0.6^{0.2}_{0.8}$	$0.6^{0.2}_{0.8}$	$0.3^{-0.1}_{0.5}$	$0.7^{0.4}_{0.8}$	$0.4^{0.1}_{0.7}$	$0.5^{0.1}_{0.7}$	$0.6^{0.2}_{0.8}$	$0.5^{0.1}_{0.7}$
TYK2	16	$0.8^{0.5}_{0.9}$	$0.9^{0.7}_{0.9}$	$0.7^{0.2}_{0.8}$	$0.7^{0.3}_{0.9}$	$0.9^{0.8}_{1.0}$	$0.8^{0.4}_{0.9}$	$0.9^{0.6}_{0.9}$	$0.8^{0.6}_{0.9}$

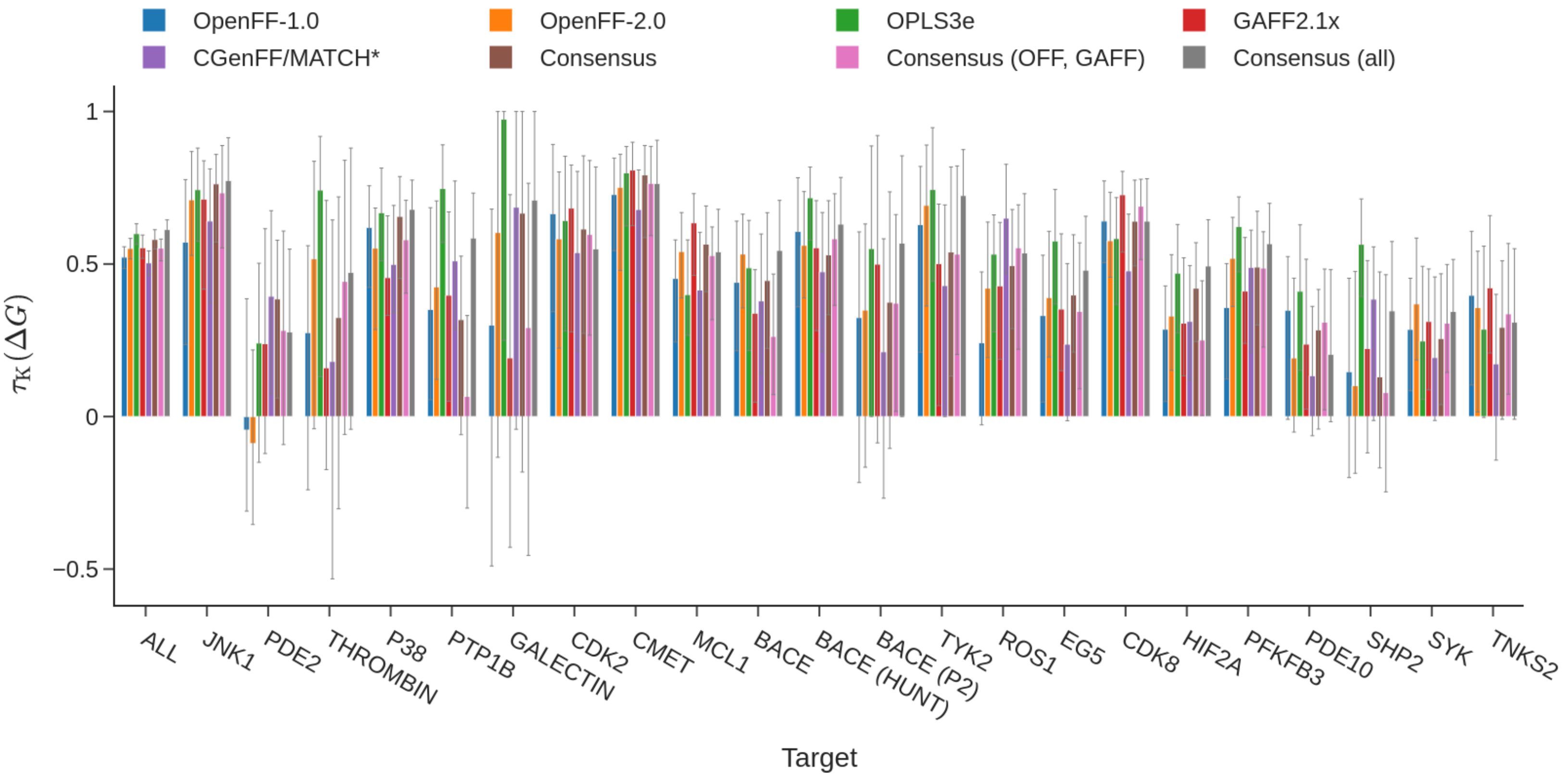

Figure S.8: *Comparison of the five force fields OpenFF-1.0, OpenFF-2.0, GAFF2.1x, CGenFF/MATCH*, OPLS3e and three consensus approaches based on the Kendall's τ_{K} of the ΔG values of the ligands.* Each group represents a target set (or 'all' for all target sets combined) with the τ_{K} values between experimental and calculated value for the respective force fields in different colors. The lower and upper bound of the 95% confidence interval are given as error bars.

Table S.9: *Comparison of the five force fields OpenFF-1.0, OpenFF-2.0, GAFF2.1x, CGenFF/MATCH*, OPLS3e and three consensus approaches based on the Kendall's* τ_K *of the* ΔG *values of the perturbations.* Each row represents a target set (or 'all' for all target sets combined) with a specified number N of perturbations followed by the τ_K between experimental and calculated value for the respective force field. The upper and lower and upper bound of the 95% confidence interval are given as sub- and superscript.

	N	τ_K							
		OpenFF -1.0	OpenFF -2.0	CGenFF/ MATCH*	GAFF 2.1x	OPLS 3e	Consensus	Consensus (OFF, GAFF)	Consensus (all)
ALL	598	$0.5_{0.6}^{0.5}$	$0.6_{0.6}^{0.5}$	$0.5_{0.5}^{0.5}$	$0.6_{0.6}^{0.5}$	$0.6_{0.6}^{0.6}$	$0.6_{0.6}^{0.5}$	$0.5_{0.6}^{0.5}$	$0.6_{0.6}^{0.6}$
BACE	36	$0.5_{0.6}^{0.2}$	$0.5_{0.7}^{0.4}$	$0.4_{0.6}^{0.2}$	$0.3_{0.5}^{0.0}$	$0.4_{0.7}^{0.2}$	$0.5_{0.7}^{0.2}$	$0.3_{0.5}^{0.1}$	$0.5_{0.7}^{0.3}$
BACE (HUNT)	32	$0.6_{0.8}^{0.4}$	$0.5_{0.8}^{0.4}$	$0.5_{0.7}^{0.2}$	$0.5_{0.7}^{0.3}$	$0.7_{0.8}^{0.6}$	$0.5_{0.7}^{0.3}$	$0.6_{0.7}^{0.4}$	$0.6_{0.8}^{0.4}$
BACE (P2)	12	$0.3_{0.6}^{-0.2}$	$0.3_{0.6}^{-0.2}$	$0.3_{0.7}^{-0.3}$	$0.5_{0.9}^{-0.1}$	$0.5_{0.9}^{-0.0}$	$0.4_{0.7}^{-0.1}$	$0.4_{0.7}^{0.0}$	$0.5_{0.8}^{0.0}$
CDK2	16	$0.7_{0.9}^{0.3}$	$0.5_{0.8}^{0.3}$	$0.6_{0.8}^{0.2}$	$0.6_{0.8}^{0.3}$	$0.6_{0.9}^{0.3}$	$0.6_{0.9}^{0.3}$	$0.6_{0.9}^{0.3}$	$0.6_{0.8}^{0.3}$
CDK8	33	$0.7_{0.8}^{0.5}$	$0.6_{0.7}^{0.5}$	$0.5_{0.7}^{0.2}$	$0.7_{0.8}^{0.5}$	$0.6_{0.7}^{0.4}$	$0.7_{0.8}^{0.5}$	$0.6_{0.8}^{0.5}$	$0.7_{0.8}^{0.5}$
CMET	24	$0.7_{0.8}^{0.5}$	$0.7_{0.9}^{0.5}$	$0.6_{0.8}^{0.4}$	$0.8_{0.9}^{0.6}$	$0.8_{0.9}^{0.6}$	$0.8_{0.9}^{0.6}$	$0.8_{0.9}^{0.6}$	$0.7_{0.9}^{0.6}$
EG5	28	$0.3_{0.5}^{0.1}$	$0.4_{0.6}^{0.2}$	$0.3_{0.5}^{-0.0}$	$0.4_{0.6}^{0.2}$	$0.6_{0.7}^{0.4}$	$0.4_{0.6}^{0.2}$	$0.4_{0.6}^{0.1}$	$0.5_{0.7}^{0.3}$
GALECTIN	8	$0.3_{0.7}^{-0.4}$	$0.6_{0.9}^{-0.0}$	$0.7_{1.0}^{0.0}$	$0.2_{0.7}^{-0.4}$	$1.0_{1.0}^{0.2}$	$0.5_{1.0}^{-0.2}$	$0.3_{0.8}^{-0.4}$	$0.7_{1.0}^{0.0}$
HIF2A	42	$0.2_{0.4}^{0.1}$	$0.4_{0.5}^{0.1}$	$0.3_{0.5}^{0.1}$	$0.4_{0.5}^{0.1}$	$0.5_{0.6}^{0.3}$	$0.4_{0.6}^{0.2}$	$0.2_{0.4}^{0.0}$	$0.5_{0.6}^{0.3}$
JNK1	21	$0.6_{0.8}^{0.3}$	$0.7_{0.9}^{0.5}$	$0.6_{0.8}^{0.4}$	$0.6_{0.8}^{0.4}$	$0.8_{0.9}^{0.6}$	$0.7_{0.9}^{0.6}$	$0.7_{0.9}^{0.6}$	$0.8_{0.9}^{0.6}$
MCL1	42	$0.4_{0.6}^{0.2}$	$0.6_{0.7}^{0.4}$	$0.4_{0.6}^{0.2}$	$0.6_{0.7}^{0.5}$	$0.5_{0.6}^{0.3}$	$0.5_{0.7}^{0.4}$	$0.4_{0.6}^{0.3}$	$0.6_{0.7}^{0.4}$
P38	34	$0.6_{0.7}^{0.4}$	$0.5_{0.7}^{0.3}$	$0.6_{0.7}^{0.3}$	$0.5_{0.7}^{0.3}$	$0.6_{0.8}^{0.5}$	$0.7_{0.8}^{0.5}$	$0.6_{0.7}^{0.4}$	$0.7_{0.8}^{0.4}$
PDE10	34	$0.2_{0.5}^{-0.0}$	$0.2_{0.5}^{-0.0}$	$0.2_{0.4}^{-0.1}$	$0.3_{0.5}^{0.0}$	$0.4_{0.6}^{0.2}$	$0.2_{0.4}^{-0.0}$	$0.3_{0.5}^{0.0}$	$0.3_{0.5}^{-0.0}$
PDE2	21	$0.0_{0.4}^{-0.3}$	$-0.1_{0.2}^{-0.4}$	$0.4_{0.7}^{0.1}$	$0.3_{0.6}^{-0.2}$	$0.2_{0.5}^{-0.2}$	$0.4_{0.6}^{0.1}$	$0.3_{0.6}^{-0.1}$	$0.3_{0.6}^{0.0}$
PFKFB3	40	$0.3_{0.5}^{0.1}$	$0.5_{0.7}^{0.4}$	$0.4_{0.6}^{0.2}$	$0.5_{0.6}^{0.2}$	$0.6_{0.7}^{0.5}$	$0.5_{0.7}^{0.3}$	$0.4_{0.6}^{0.2}$	$0.6_{0.7}^{0.4}$
PTP1B	23	$0.4_{0.7}^{0.1}$	$0.4_{0.7}^{0.1}$	$0.6_{0.8}^{0.3}$	$0.3_{0.7}^{0.0}$	$0.7_{0.9}^{0.6}$	$0.2_{0.5}^{-0.0}$	$0.0_{0.3}^{-0.3}$	$0.6_{0.7}^{0.3}$
ROS1	28	$0.3_{0.5}^{-0.0}$	$0.5_{0.6}^{0.2}$	$0.7_{0.8}^{0.5}$	$0.4_{0.6}^{0.2}$	$0.5_{0.7}^{0.3}$	$0.5_{0.7}^{0.3}$	$0.5_{0.7}^{0.2}$	$0.6_{0.7}^{0.3}$
SHP2	26	$0.1_{0.4}^{-0.2}$	$0.2_{0.4}^{-0.2}$	$0.3_{0.6}^{-0.0}$	$0.2_{0.5}^{-0.1}$	$0.5_{0.7}^{0.4}$	$0.1_{0.4}^{-0.2}$	$0.2_{0.5}^{-0.2}$	$0.2_{0.6}^{0.1}$
SYK	44	$0.3_{0.5}^{0.1}$	$0.4_{0.6}^{0.2}$	$0.2_{0.4}^{-0.0}$	$0.3_{0.5}^{0.1}$	$0.3_{0.5}^{0.1}$	$0.2_{0.5}^{0.0}$	$0.3_{0.5}^{0.2}$	$0.3_{0.5}^{0.1}$
THROMBIN	11	$0.2_{0.6}^{-0.3}$	$0.4_{0.8}^{-0.0}$	$-0.0_{0.7}^{-0.6}$	$0.2_{0.7}^{-0.2}$	$0.6_{0.9}^{0.2}$	$0.3_{0.7}^{-0.3}$	$0.3_{0.8}^{-0.1}$	$0.5_{0.9}^{0.0}$
TNKS2	27	$0.4_{0.6}^{0.1}$	$0.3_{0.6}^{0.0}$	$0.1_{0.4}^{-0.1}$	$0.4_{0.7}^{0.2}$	$0.3_{0.5}^{0.0}$	$0.3_{0.5}^{-0.0}$	$0.4_{0.6}^{0.1}$	$0.3_{0.5}^{-0.0}$
TYK2	16	$0.6_{0.8}^{0.2}$	$0.7_{0.9}^{0.4}$	$0.4_{0.7}^{0.0}$	$0.4_{0.7}^{-0.0}$	$0.8_{0.9}^{0.4}$	$0.6_{0.8}^{0.2}$	$0.6_{0.8}^{0.2}$	$0.7_{0.9}^{0.4}$

Correlation plots

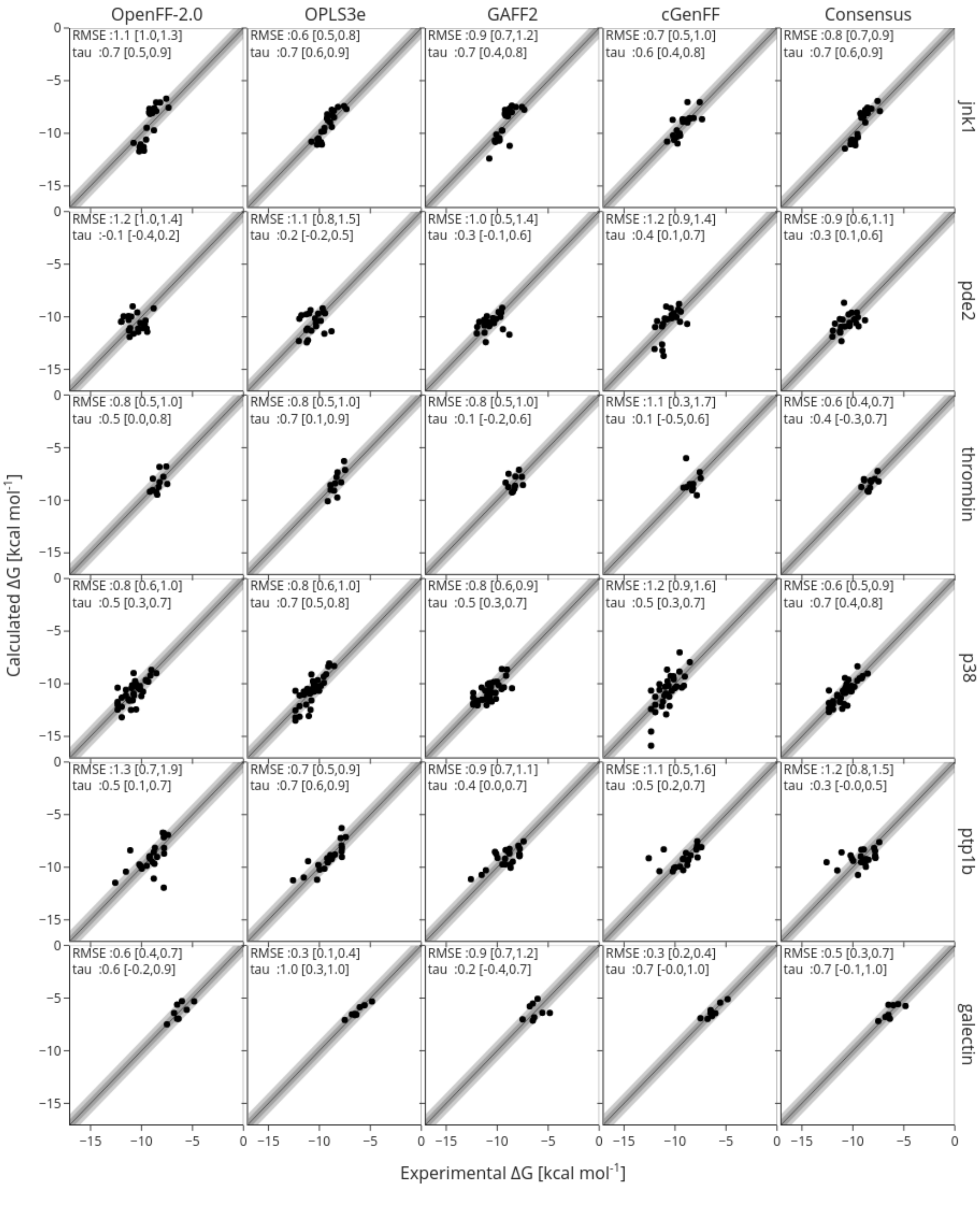

Figure S.9: *Correlation of the ΔG values obtained from calculations with the force fields OpenFF-2.0, GAFF2.1x, CGenFF/MATCH*, OPLS3e and the Consensus approach versus the experimentally derived values.* Each column of plots correspond to one target. In each plot, the RMSE in kcal mol^{-1}and Kendall's τ is listed with the 95% confidence interval. Correlation plots for the other targets are found in Figures S.10, S.11 and S.12.

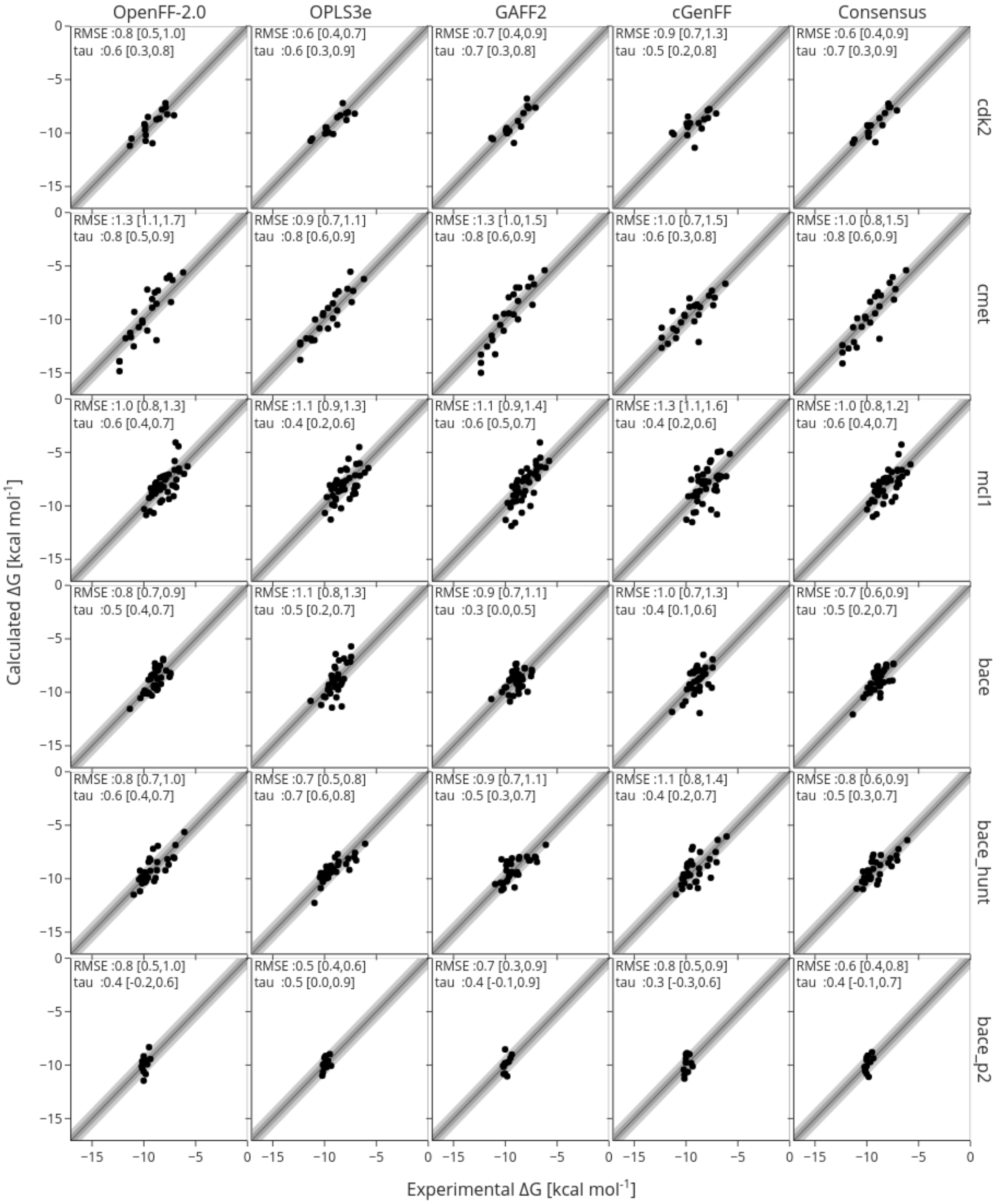

Figure S.10: *Correlation of the ΔG values obtained from calculations with the force fields OpenFF-2.0, GAFF2.1x, CGenFF/MATCH*, OPLS3e and the Consensus approach versus the experimentally derived values.* Each column of plots correspond to one target. In each plot, the RMSE in kcal mol^{-1}and Kendall's τ is listed with the 95% confidence interval. Correlation plots for the other targets are found in Figures S.9, S.11 and S.12.

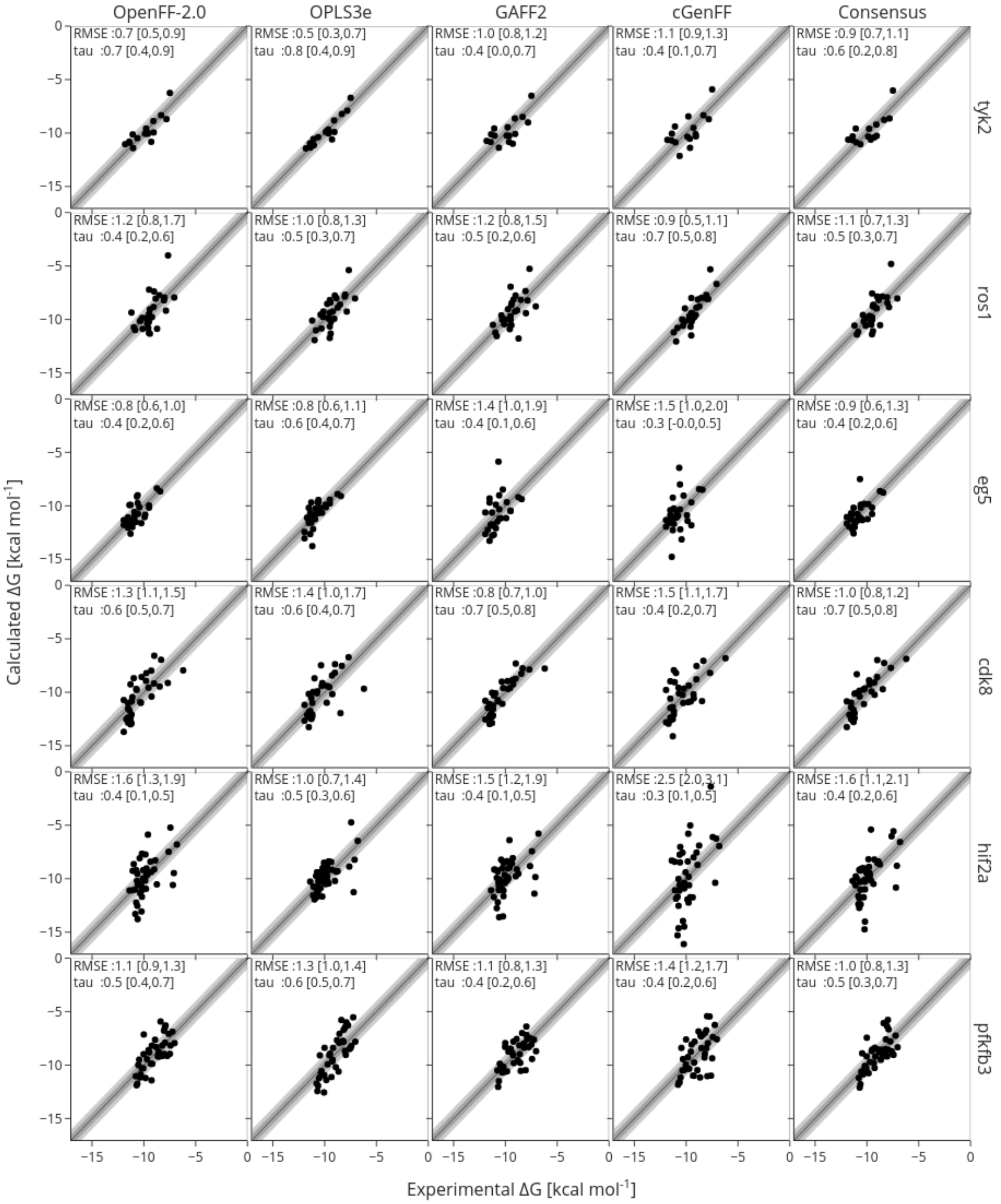

Figure S.11: *Correlation of the ΔG values obtained from calculations with the force fields OpenFF-2.0, GAFF2.1x, CGenFF/MATCH*, OPLS3e and the Consensus approach versus the experimentally derived values.* Each column of plots correspond to one target. In each plot, the RMSE in kcal mol^{-1}and Kendall's τ is listed with the 95% confidence interval. Correlation plots for the other targets are found in Figures S.9, S.10 and S.12.

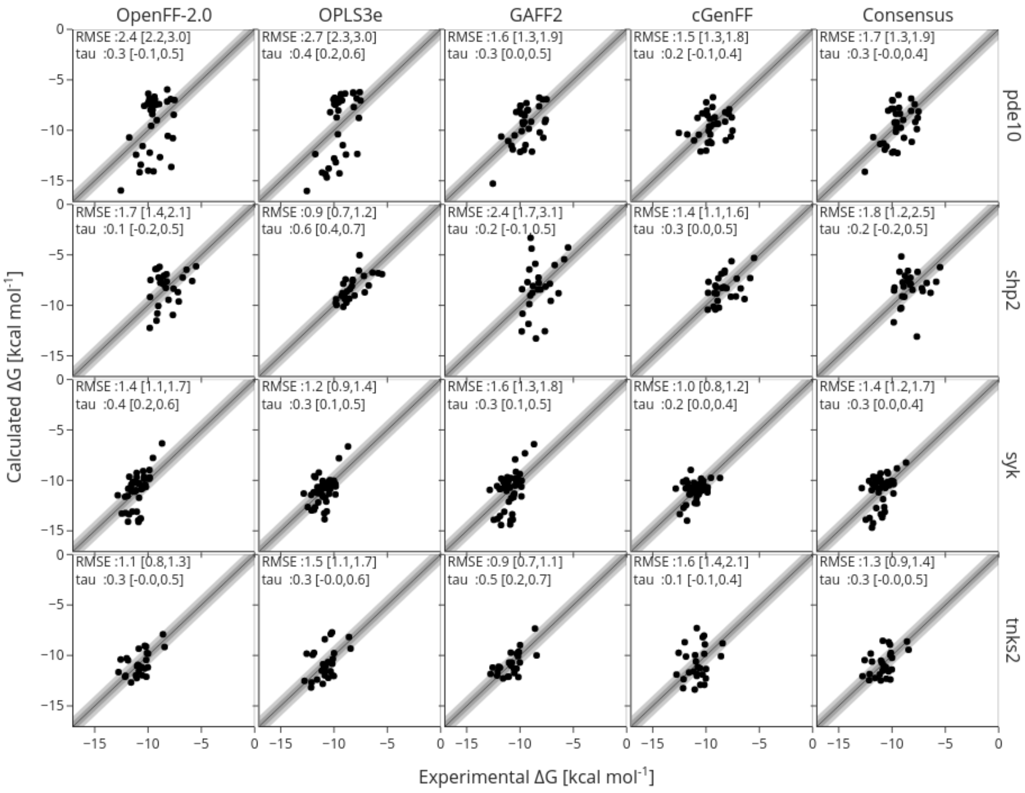

Figure S.12: *Correlation of the ΔG values obtained from calculations with the force fields OpenFF-2.0, GAFF2.1x, CGenFF/MATCH*, OPLS3e and the Consensus approach versus the experimentally derived values.* Each column of plots correspond to one target. In each plot, the RMSE in kcal mol^{-1}and Kendall's τ is listed with the 95% confidence interval. Correlation plots for the other targets are found in Figures S.9, S.10 and S.11.

Additional analysis to the convergence measure α

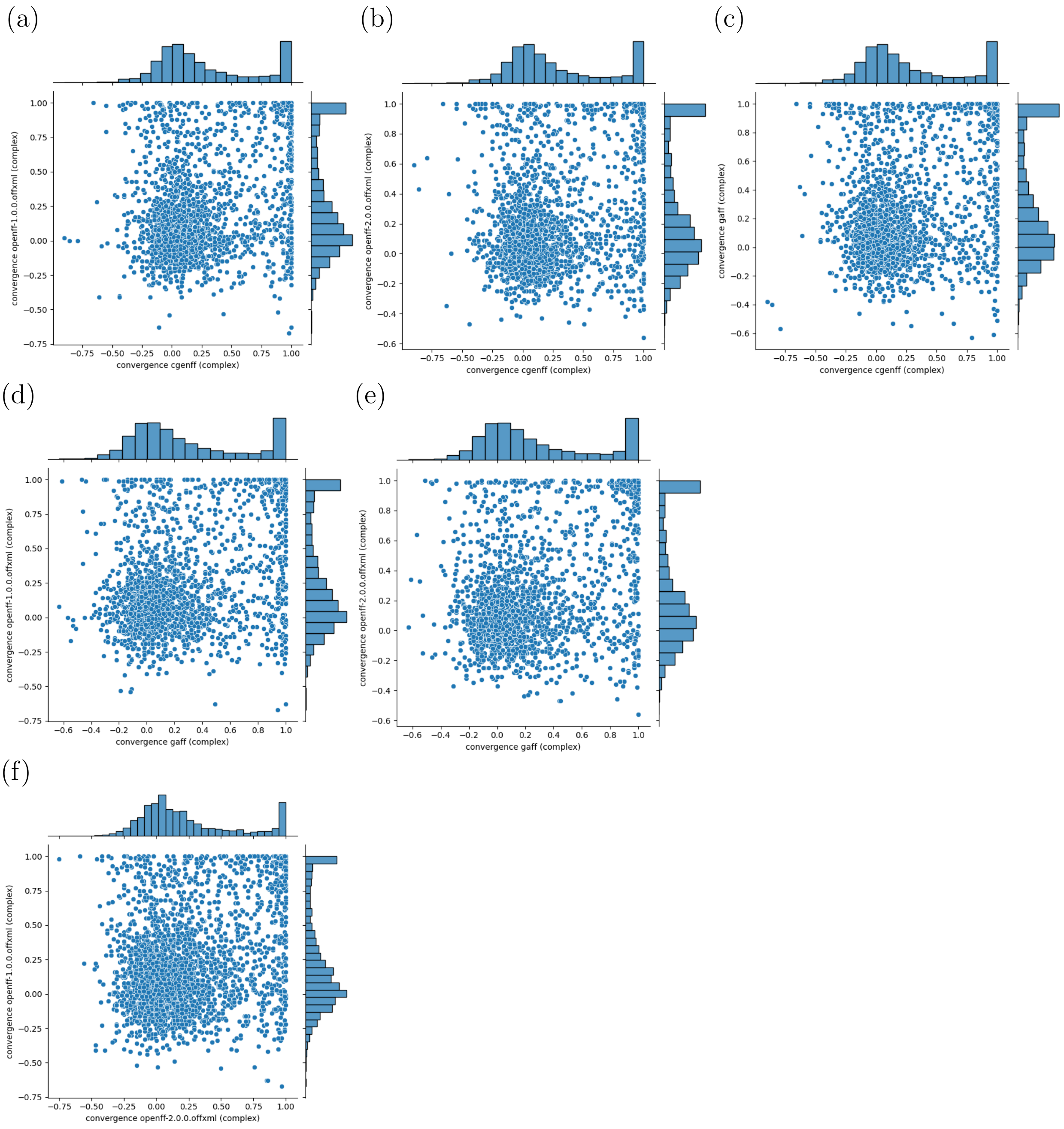

Figure S.13: *Comparison of the convergence measure α for the force fields OpenFF-1.0, OpenFF-2.0, GAFF2.1x and CGenFF/MATCH*.* The central plot of each panel shows the correlation between the convergence measures α of simulations of the protein-ligand complexes performed with two different force fields, as specified in the axes labels. The corresponding histograms of α of the simulations with the force fields are shown at the top and the right of the central plot.

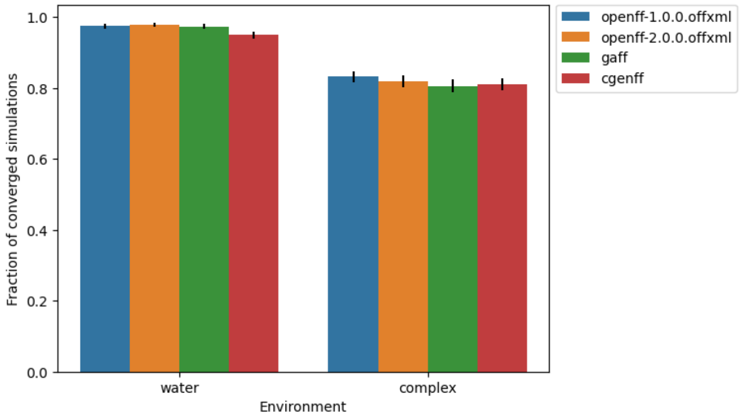

Figure S.14: *Ratio of converged simulations of ligands in water or in complex with the force fields OpenFF-1.0, OpenFF-2.0, GAFF2.1x and CGenFF/MATCH*.* The bars shows the fractions of simulations with a convergence measure $\alpha < 0.8$, which are considered converged in this manuscript. The error bars show the lower and upper ends of the 95% confidence interval, as determined by bootstrapping using 1000 bootstrap samples.

Analysis of the effect of off-site charges

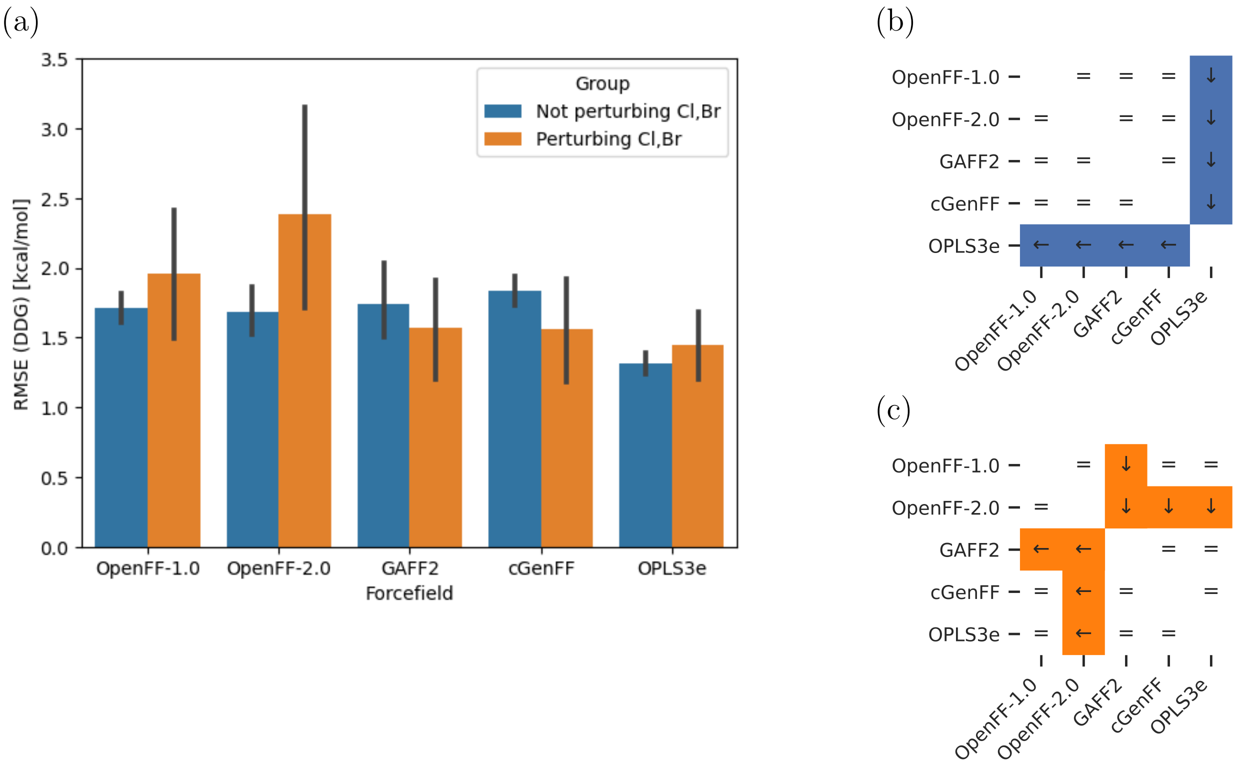

Figure S.15: *Analysis of the effect of off-site charges for chlorine and bromine in binding free energy estimates calculated from simulations using GAFF2.1x and CGenFF/MATCH*.* We separately analyzed 51 perturbations concerning chlorine and bromine and the 1077 perturbations not concerning chlorine and bromine. Panel (a) The RMSE of calculated $\Delta\Delta G$ values compared to experiment are shown across the edges not perturbing clorine and bromine (blue) and the edges perturbing clorine and bromine (orange). Whereas the edges perturbing clorine and bromine (orange bars) perform worse compared to the remaining edges (blue bars) for the OpenFF forcefields, they perform better for GAFF2.1x and CGenFF/MATCH*, which both employ virtual sites for chlorine and bromine atoms. Therefore, GAFF2.1x and CGenFF/MATCH* may benefit from the virtual sites. Panel (b) Illustrations of significance of differences between the sets concerning different forcefields and edges not perturbing clorine and bromine. Panel (c): Shows the same as Panel (b), but for the edges perturbing clorine and bromine. In panels (b) and (c), a white matrix element with an equal sign ("=") means that the differences between the two force fields are statistically insignificant. A colored matrix element denotes a significant difference considering a 95% confidence interval. The arrow in a colored matrix element points at the force field which has the lower error (either left or down). It is important to note that our observations regarding force field differences across the whole set of 1116 perturbations still hold true irrespective of the virtual sites, as only a small subset of perturbations ($< 5\%$) is affected by the addition of virtual sites. The same conclusions can be drawn from Figures 1a and 1b and the blue bars and matrix Panel (b).

Determinants of accuracy

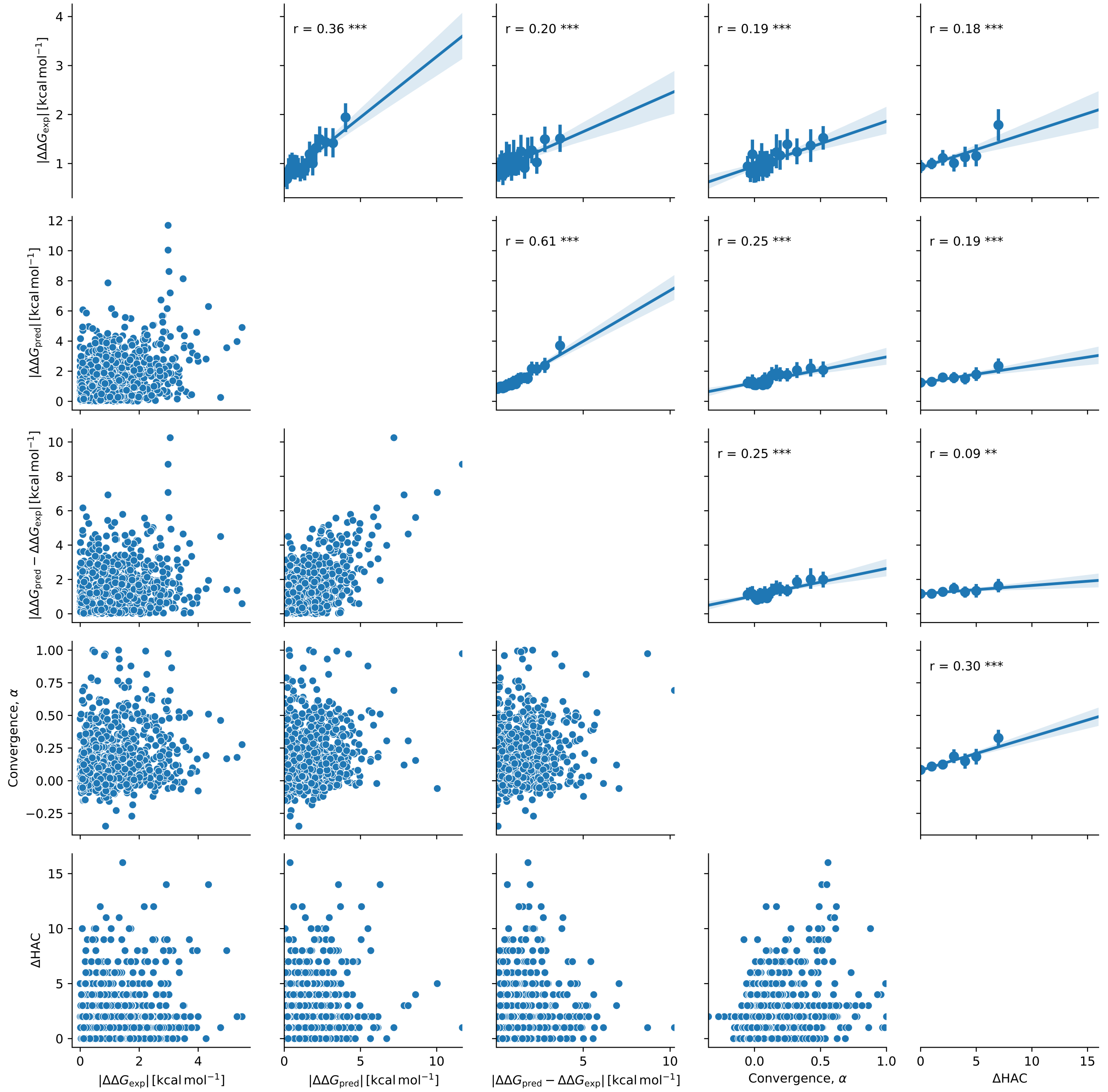

Figure S.16: *Visualization of pairwise relationships between the experimental relative free energies $\Delta\Delta G_{\text{exp.}}$, the calculated relative free energies $\Delta\Delta G_{\text{pred}}$ (OpenFF-1.0), the absolute error between experimental and calculated values $|\Delta\Delta G_{\text{pred}} - \Delta\Delta G_{\text{exp.}}|$, the average convergence measure α[33] (averaged over three solvent and three complex OpenFF-1.0 simulation legs), the change in number of heavy atoms in the end states.* The lower left triangle shows the original 1116 datapoints. The upper right triangle plots show linear regression plots. The Pearson's correlation coefficient is given in the graph together with its p-value indicated as stars (one, two, or three stars for a confidence level of < 0.05, < 0.01, and < 0.001, respectively). For illustration purposes, the data was binned into 20 bins and their average with standard deviation are shown as dot with error bars. The regression was performed on the original data.

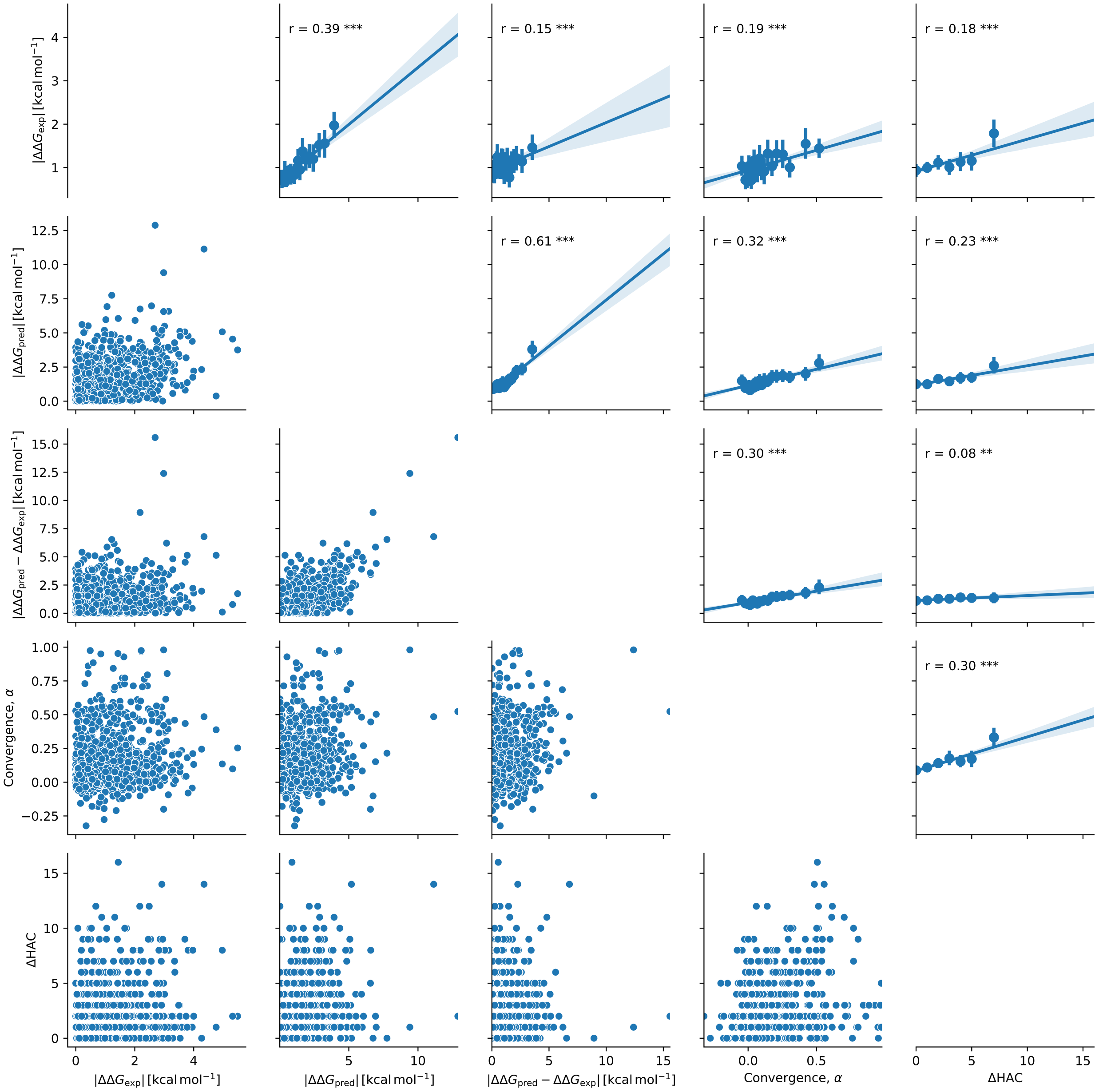

Figure S.17: *Visualization of pairwise relationships between the experimental relative free energies $\Delta\Delta G_{\text{exp.}}$, the calculated relative free energies $\Delta\Delta G_{\text{pred}}$ (OpenFF-2.0), the absolute error between experimental and calculated values $|\Delta\Delta G_{\text{pred}} - \Delta\Delta G_{\text{exp.}}|$, the average convergence measure α[33] (averaged over three solvent and three complex OpenFF-2.0 simulation legs), the change in number of heavy atoms in the end states.* The lower left triangle shows the original 1116 datapoints. The upper right triangle plots show linear regression plots. The Pearson's correlation coefficient is given in the graph together with its p-value indicated as stars (one, two, or three stars for a confidence level of < 0.05, < 0.01, and < 0.001, respectively). For illustration purposes, the data was binned into 20 bins and their average with standard deviation are shown as dot with error bars. The regression was performed on the original data.

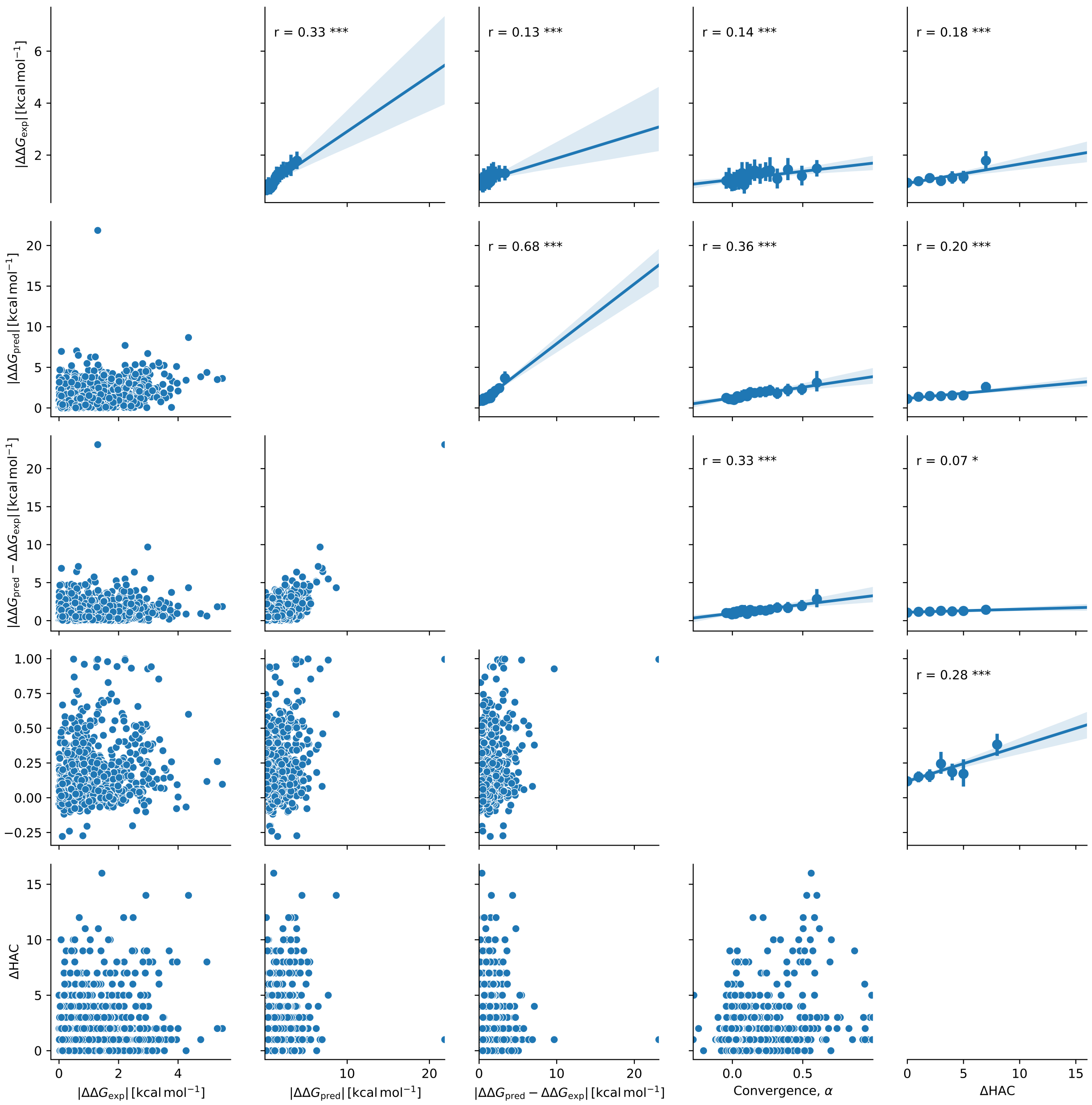

Figure S.18: *Visualization of pairwise relationships between the experimental relative free energies $\Delta\Delta G_{\text{exp.}}$, the calculated relative free energies $\Delta\Delta G_{\text{pred}}$ (GAFF2.1x), the absolute error between experimental and calculated values $|\Delta\Delta G_{\text{pred}} - \Delta\Delta G_{\text{exp.}}|$, the average convergence measure α[33] (averaged over three solvent and three complex GAFF2.1x simulation legs), the change in number of heavy atoms in the end states.* The lower left triangle shows the original 1116 datapoints. The upper right triangle plots show linear regression plots. The Pearson's correlation coefficient is given in the graph together with its p-value indicated as stars (one, two, or three stars for a confidence level of < 0.05, < 0.01, and < 0.001, respectively). For illustration purposes, the data was binned into 20 bins and their average with standard deviation are shown as dot with error bars. The regression was performed on the original data.

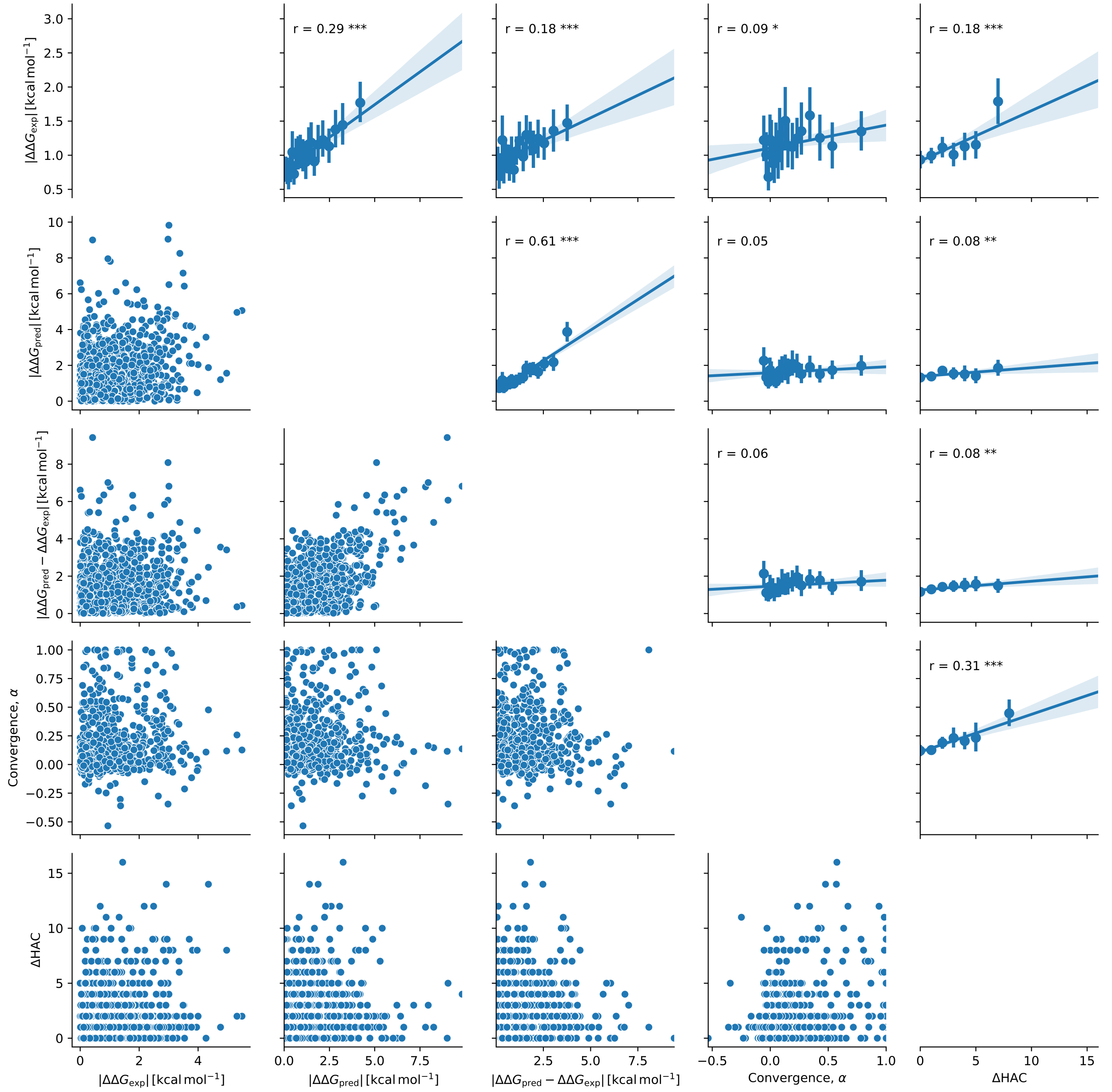

Figure S.19: *Visualization of pairwise relationships between the experimental relative free energies* $\Delta\Delta G_{\mathrm{exp.}}$*, the calculated relative free energies* $\Delta\Delta G_{\mathrm{pred}}$ *(CGenFF/MATCH*), the absolute error between experimental and calculated values* $|\Delta\Delta G_{\mathrm{pred}} - \Delta\Delta G_{\mathrm{exp.}}|$*, the average convergence measure* α[33] *(averaged over three solvent and three complex CGenFF/MATCH* simulation legs), the change in number of heavy atoms in the end states.* The lower left triangle shows the original 1116 datapoints. The upper right triangle plots show linear regression plots. The Pearson's correlation coefficient is given in the graph together with its p-value indicated as stars (one, two, or three stars for a confidence level of < 0.05, < 0.01, and < 0.001, respectively). For illustration purposes, the data was binned into 20 bins and their average with standard deviation are shown as dot with error bars. The regression was performed on the original data.

Accuracy dependent on perturbation properties

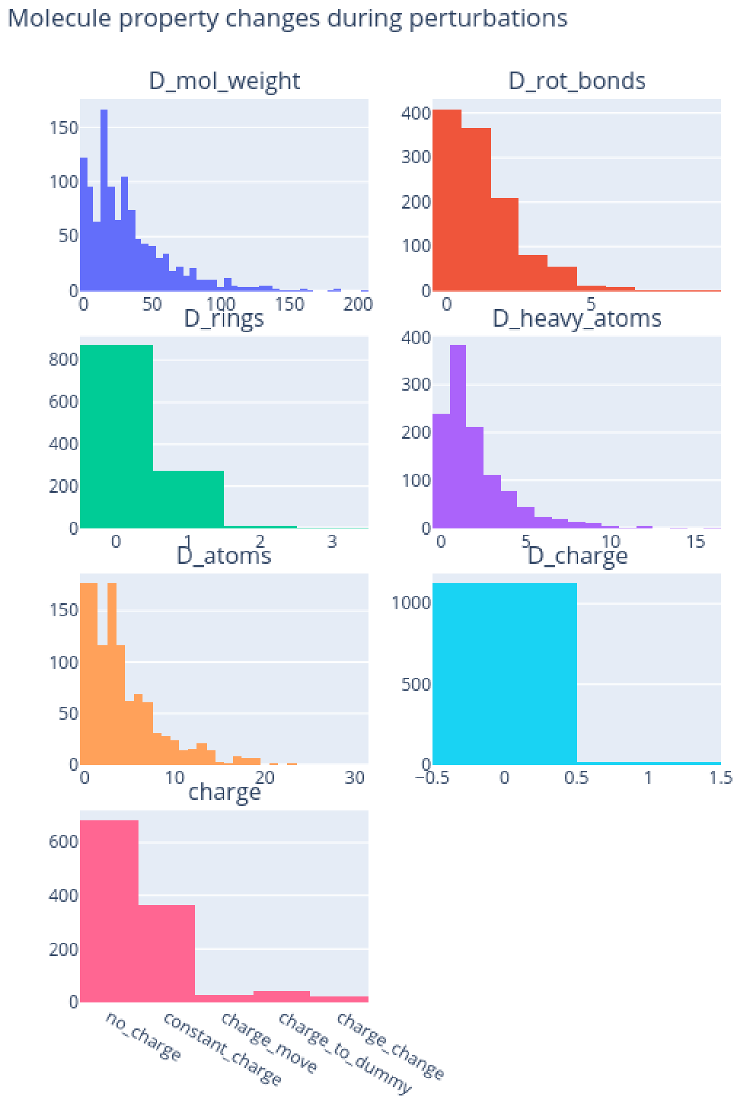

Figure S.20: *Illustration of property changes of all considered perturbations.* The histograms show the absolute changes in the following molecular properties during the perturbations: molecular weight, number of rotational bonds, number of rings, number of heavy atoms, number of atoms, charge change (0 means no charge change), and type of charge perturbation. In the bottom histogram, the perturbations with zero charge change are also separated into the categories uncharged, charged, charged where the charge moves and charge into dummy (and vice versa), depending on the mapping of the two molecules onto each other.

The accuracy decreases with more difficult perturbations We categorized the perturbations into different levels of difficulty by using molecular properties of the two end states. Histograms of the different properties can be found in Figure S.20.

The following Figures S.21-S.25 show the mean unsigned error between the calculated results for various forcefields and experiment of subsets categorized by the different properties of the perturbations.

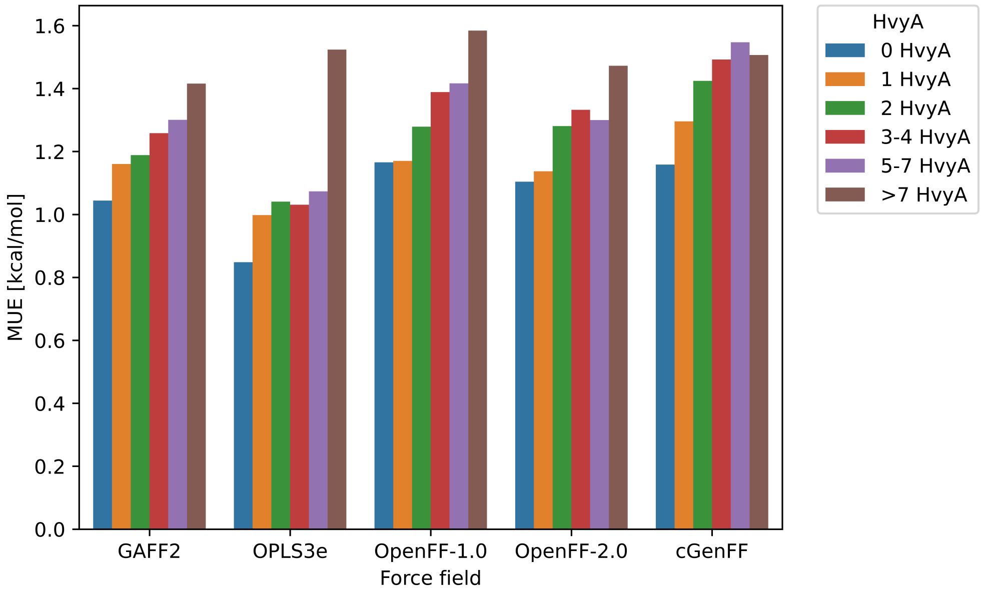

Figure S.21: The mean unsigned error increases with increasing heavy atom difference between the end states of alchemical perturbations. The bars show the mean unsigned error of $\Delta\Delta G$ values compared between calculations and experiment, categorized by the heavy atom difference between the two end states. Note that the difference in number of heavy atoms does not tell anything about the number of perturbed atoms, i.e. a perturbation from a cyclohexyl moiety to a pyridyl moiety would have a have an atom difference of 0 (both have 6 heavy atoms), but the number of perturbed atoms would be 12.

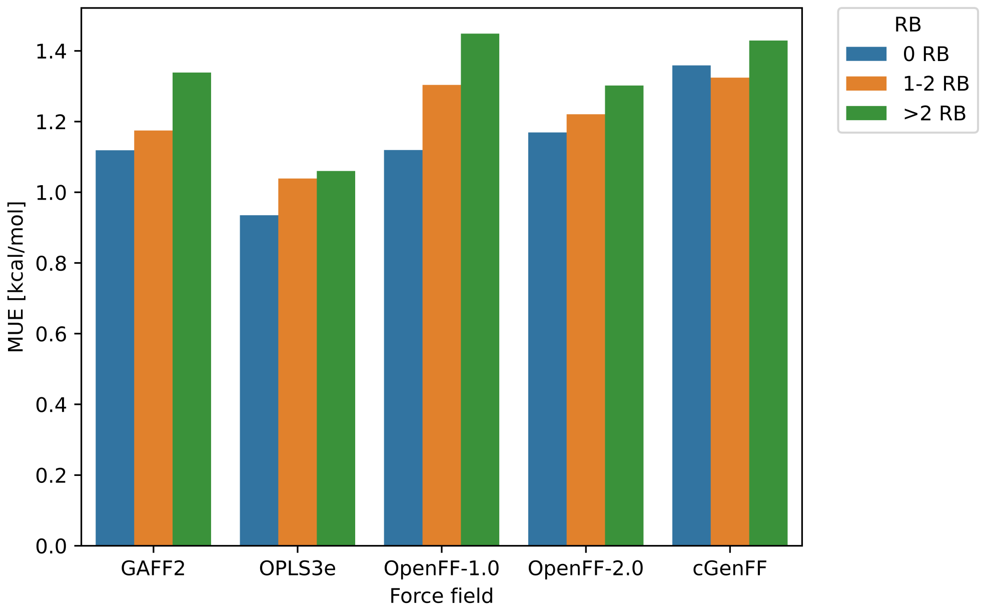

Figure S.22: The mean unsigned error increases with increasing number of rotational bonds difference between the end states of alchemical perturbations. The bars show the mean unsigned error of $\Delta\Delta G$ values compared between calculations and experiment, categorized by the number of rotational bonds difference between the two end states.

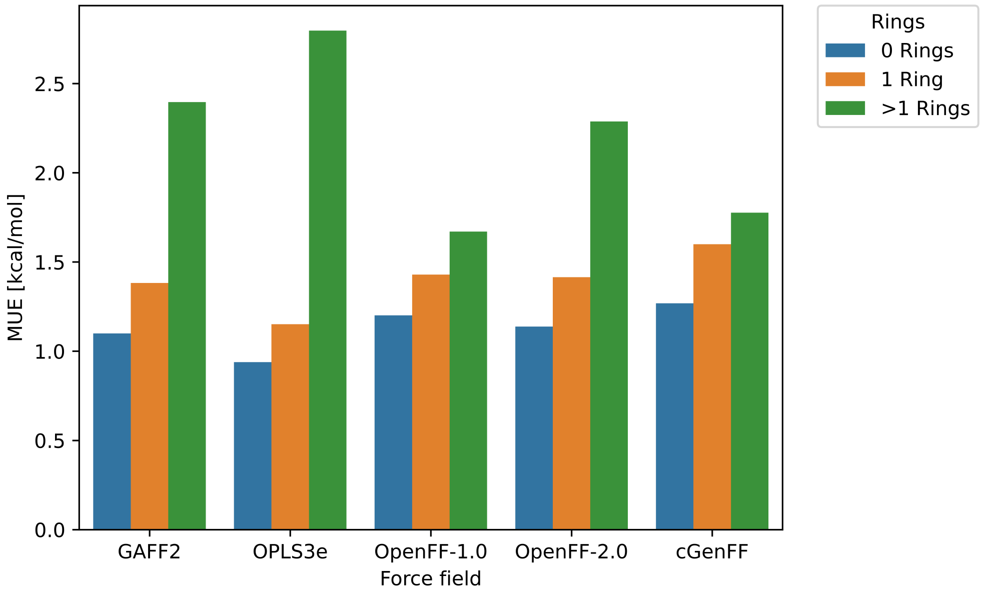

Figure S.23: The mean unsigned error increases with increasing number of rings difference between the end states of alchemical perturbations. The bars show the mean unsigned error of $\Delta\Delta G$ values compared between calculations and experiment, categorized by the number of rings difference between the two end states.

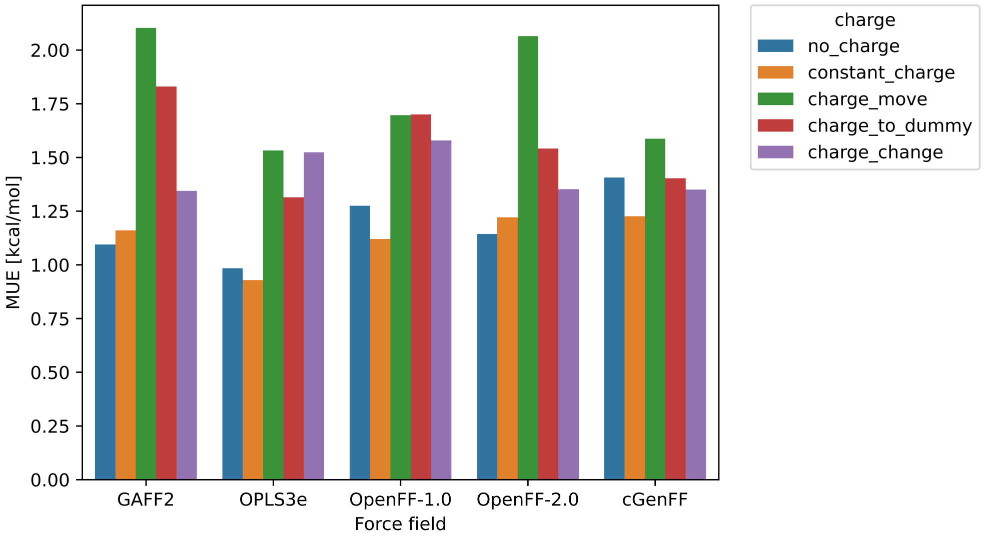

Figure S.24: The mean unsigned error increases with more difficult charge categories of the perturbations. The bars show the mean unsigned error of $\Delta\Delta G$ values compared between calculations and experiment, categorized by type of charge change of the perturbation.

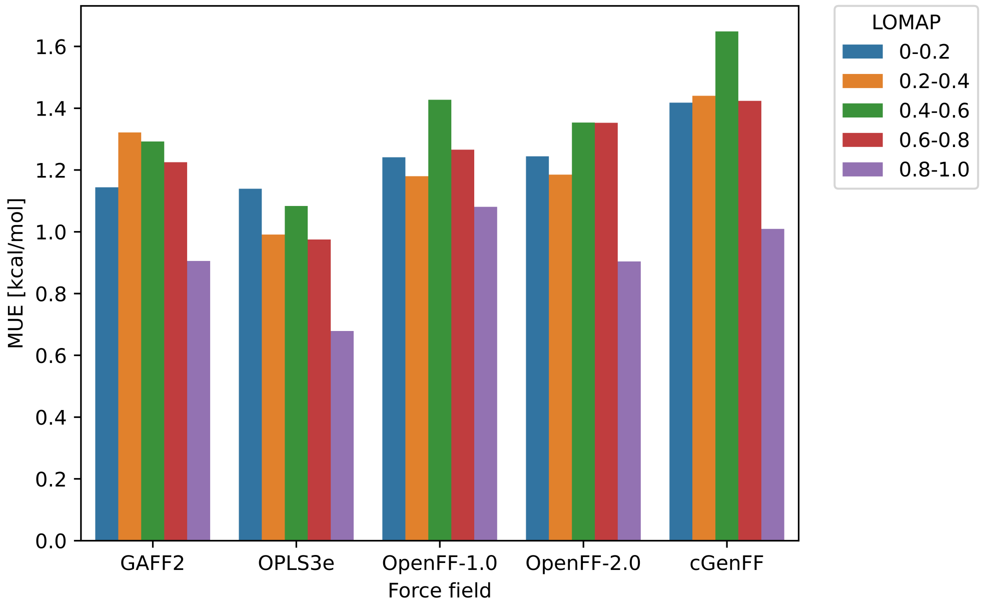

Figure S.25: The mean unsigned error increases with decreasing LOMAP scores of alchemical perturbations. The bars show the mean unsigned error of $\Delta\Delta G$ values compared between calculations and experiment, categorized by the LOMAP score between the two end states.

References

(1) Cumming, J. N.; Smith, E. M.; Wang, L.; Misiaszek, J.; Durkin, J.; Pan, J.; Iserloh, U.; Wu, Y.; Zhu, Z.; Strickland, C.; Voigt, J.; Chen, X.; Kennedy, M. E.; Kuvelkar, R.; Hyde, L. A.; Cox, K.; Favreau, L.; Czarniecki, M. F.; Greenlee, W. J.; McKittrick, B. A.; Parker, E. M.; Stamford, A. W. Structure based design of iminohydantoin BACE1 inhibitors: Identification of an orally available, centrally active BACE1 inhibitor. *Bioorg. Med. Chem. Lett.* **2012**, *22*, 2444–2449, tex.ids: cumming_structure_2012a.

(2) Wang, L.; Wu, Y.; Deng, Y.; Kim, B.; Pierce, L.; Krilov, G.; Lupyan, D.; Robinson, S.; Dahlgren, M. K.; Greenwood, J.; Romero, D. L.; Masse, C.; Knight, J. L.; Steinbrecher, T.; Beuming, T.; Damm, W.; Harder, E.; Sherman, W.; Brewer, M.; Wester, R.; Murcko, M.; Frye, L.; Farid, R.; Lin, T.; Mobley, D. L.; Jorgensen, W. L.; Berne, B. J.; Friesner, R. A.; Abel, R. Accurate and Reliable Prediction of Relative Ligand Binding Potency in Prospective Drug Discovery by Way of a Modern Free-Energy Calculation Protocol and Force Field. *J. Am. Chem. Soc.* **2015**, *137*, 2695–2703.

(3) Hunt, K. W.; Cook, A. W.; Watts, R. J.; Clark, C. T.; Vigers, G.; Smith, D.; Metcalf, A. T.; Gunawardana, I. W.; Burkard, M.; Cox, A. A.; Geck Do, M. K.; Dutcher, D.; Thomas, A. A.; Rana, S.; Kallan, N. C.; DeLisle, R. K.; Rizzi, J. P.; Regal, K.; Sammond, D.; Groneberg, R.; Siu, M.; Purkey, H.; Lyssikatos, J. P.; Marlow, A.; Liu, X.; Tang, T. P. Spirocyclic β-Site Amyloid Precursor Protein Cleaving Enzyme 1 (BACE1) Inhibitors: From Hit to Lowering of Cerebrospinal Fluid (CSF) Amyloid β in a Higher Species. *J. Med. Chem.* **2013**, *56*, 3379–3403.

(4) Ciordia, M.; Pérez-Benito, L.; Delgado, F.; Trabanco, A. A.; Tresadern, G. Application of Free Energy Perturbation for the Design of BACE1 Inhibitors. *J. Chem. Inf. Model.* **2016**, *56*, 1856–1871.

(5) Keränen, H.; Pérez-Benito, L.; Ciordia, M.; Delgado, F.; Steinbrecher, T. B.;

Oehlrich, D.; van Vlijmen, H. W. T.; Trabanco, A. A.; Tresadern, G. Acylguanidine Beta Secretase 1 Inhibitors: A Combined Experimental and Free Energy Perturbation Study. *J. Chem. Theory Comput.* **2017**, *13*, 1439–1453.

(6) Malamas, M. S.; Erdei, J.; Gunawan, I.; Turner, J.; Hu, Y.; Wagner, E.; Fan, K.; Chopra, R.; Olland, A.; Bard, J.; Jacobsen, S.; Magolda, R. L.; Pangalos, M.; Robichaud, A. J. Design and Synthesis of 5,5′-Disubstituted Aminohydantoins as Potent and Selective Human β-Secretase (BACE1) Inhibitors. *J. Med. Chem.* **2010**, *53*, 1146–1158.

(7) Hardcastle, I. R.; Arris, C. E.; Bentley, J.; Boyle, F. T.; Chen, Y.; Curtin, N. J.; Endicott, J. A.; Gibson, A. E.; Golding, B. T.; Griffin, R. J.; Jewsbury, P.; Menyerol, J.; Mesguiche, V.; Newell, D. R.; Noble, M. E. M.; Pratt, D. J.; Wang, L.-Z.; Whitfield, H. J. N ^{2} -Substituted *O* ^{6} -Cyclohexylmethylguanine Derivatives: Potent Inhibitors of Cyclin-Dependent Kinases 1 and 2. *J. Med. Chem.* **2004**, *47*, 3710–3722.

(8) Schiemann, K.; Mallinger, A.; Wienke, D.; Esdar, C.; Poeschke, O.; Busch, M.; Rohdich, F.; Eccles, S. A.; Schneider, R.; Raynaud, F. I.; Czodrowski, P.; Musil, D.; Schwarz, D.; Urbahns, K.; Blagg, J. Discovery of Potent and Selective CDK8 Inhibitors from an HSP90 Pharmacophore. *Bioorg. Med. Chem. Lett.* **2016**, *26*, 1443–1451.

(9) Schindler, C. E. M.; Baumann, H.; Blum, A.; Böse, D.; Buchstaller, H.-P.; Burgdorf, L.; Cappel, D.; Chekler, E.; Czodrowski, P.; Dorsch, D.; Eguida, M. K. I.; Follows, B.; Fuchß, T.; Grädler, U.; Gunera, J.; Johnson, T.; Jorand Lebrun, C.; Karra, S.; Klein, M.; Knehans, T.; Koetzner, L.; Krier, M.; Leiendecker, M.; Leuthner, B.; Li, L.; Mochalkin, I.; Musil, D.; Neagu, C.; Rippmann, F.; Schiemann, K.; Schulz, R.; Steinbrecher, T.; Tanzer, E.-M.; Unzue Lopez, A.; Viacava Follis, A.; Wegener, A.; Kuhn, D. Large-Scale Assessment of Binding Free Energy Calculations in Active Drug Discovery Projects. *J. Chem. Inf. Model.* **2020**, *60*, 5457–5474.

(10) Dorsch, D.; Schadt, O.; Stieber, F.; Meyring, M.; Grädler, U.; Bladt, F.; Friese-Hamim, M.; Knühl, C.; Pehl, U.; Blaukat, A. Identification and optimization of pyridazinones as potent and selective c-Met kinase inhibitors. *Bioorg. Med. Chem. Lett.* **2015**, *25*, 1597–1602.

(11) Schiemann, K.; Finsinger, D.; Zenke, F.; Amendt, C.; Knöchel, T.; Bruge, D.; Buchstaller, H.-P.; Emde, U.; Stähle, W.; Anzali, S. The Discovery and Optimization of Hexahydro-2H-Pyrano[3,2-c]Quinolines (HHPQs) as Potent and Selective Inhibitors of the Mitotic Kinesin-5. *Bioorg. Med. Chem. Lett.* **2010**, *20*, 1491–1495.

(12) Delaine, T.; Collins, P.; MacKinnon, A.; Sharma, G.; Stegmayr, J.; Rajput, V. K.; Mandal, S.; Cumpstey, I.; Larumbe, A.; Salameh, B. A.; Kahl-Knutsson, B.; van Hattum, H.; van Scherpenzeel, M.; Pieters, R. J.; Sethi, T.; Schambye, H.; Oredsson, S.; Leffler, H.; Blanchard, H.; Nilsson, U. J. Galectin-3-Binding Glycomimetics That Strongly Reduce Bleomycin-Induced Lung Fibrosis and Modulate Intracellular Glycan Recognition. *ChemBioChem* **2016**, *17*, 1759–1770.

(13) Manzoni, F.; Ryde, U. Assessing the Stability of Free-Energy Perturbation Calculations by Performing Variations in the Method. *J. Comput.-Aided Mol. Des.* **2018**, *32*, 529–536.

(14) Wallace, E. M.; Rizzi, J. P.; Han, G.; Wehn, P. M.; Cao, Z.; Du, X.; Cheng, T.; Czerwinski, R. M.; Dixon, D. D.; Goggin, B. S.; Grina, J. A.; Halfmann, M. M.; Maddie, M. A.; Olive, S. R.; Schlachter, S. T.; Tan, H.; Wang, B.; Wang, K.; Xie, S.; Xu, R.; Yang, H.; Josey, J. A. A Small-Molecule Antagonist of HIF2α Is Efficacious in Preclinical Models of Renal Cell Carcinoma. *Cancer Res.* **2016**, *76*, 5491–5500.

(15) Szczepankiewicz, B. G.; Kosogof, C.; Nelson, L. T. J.; Liu, G.; Liu, B.; Zhao, H.; Serby, M. D.; Xin, Z.; Liu, M.; Gum, R. J.; Haasch, D. L.; Wang, S.; Clampit, J. E.; Johnson, E. F.; Lubben, T. H.; Stashko, M. A.; Olejniczak, E. T.; Sun, C.; Dor-

win, S. A.; Haskins, K.; Abad-Zapatero, C.; Fry, E. H.; Hutchins, C. W.; Sham, H. L.; Rondinone, C. M.; Trevillyan, J. M. Aminopyridine-Based c-Jun N-Terminal Kinase Inhibitors with Cellular Activity and Minimal Cross-Kinase Activity †. *J. Med. Chem.* **2006**, *49*, 3563–3580.

(16) Friberg, A.; Vigil, D.; Zhao, B.; Daniels, R. N.; Burke, J. P.; Garcia-Barrantes, P. M.; Camper, D.; Chauder, B. A.; Lee, T.; Olejniczak, E. T.; Fesik, S. W. Discovery of Potent Myeloid Cell Leukemia 1 (Mcl-1) Inhibitors Using Fragment-Based Methods and Structure-Based Design. *J. Med. Chem.* **2013**, *56*, 15–30.

(17) Goldstein, D. M.; Soth, M.; Gabriel, T.; Dewdney, N.; Kuglstatter, A.; Arzeno, H.; Chen, J.; Bingenheimer, W.; Dalrymple, S. A.; Dunn, J.; Farrell, R.; Frauchiger, S.; La Fargue, J.; Ghate, M.; Graves, B.; Hill, R. J.; Li, F.; Litman, R.; Loe, B.; McIntosh, J.; McWeeney, D.; Papp, E.; Park, J.; Reese, H. F.; Roberts, R. T.; Rotstein, D.; San Pablo, B.; Sarma, K.; Stahl, M.; Sung, M.-L.; Suttman, R. T.; Sjogren, E. B.; Tan, Y.; Trejo, A.; Welch, M.; Weller, P.; Wong, B. R.; Zecic, H. Discovery of 6-(2,4-Difluorophenoxy)-2-[3-Hydroxy-1-(2-Hydroxyethyl)Propylamino]-8-Methyl-8 *H* -Pyrido[2,3- *d*]Pyrimidin-7-One (Pamapimod) and 6-(2,4-Difluorophenoxy)-8-Methyl-2-(Tetrahydro-2 *H* -Pyran-4-Ylamino)Pyrido[2,3- *d*]Pyrimidin-7(8 *H*)-One (R1487) as Orally Bioavailable and Highly Selective Inhibitors of P38α Mitogen-Activated Protein Kinase. *J. Med. Chem.* **2011**, *54*, 2255–2265.

(18) Buijnsters, P.; De Angelis, M.; Langlois, X.; Rombouts, F. J. R.; Sanderson, W.; Tresadern, G.; Ritchie, A.; Trabanco, A. A.; VanHoof, G.; Roosbroeck, Y. V.; Andrés, J.-I. Structure-Based Design of a Potent, Selective, and Brain Penetrating PDE2 Inhibitor with Demonstrated Target Engagement. *ACS Med. Chem. Lett.* **2014**, *5*, 1049–1053.

(19) Pérez-Benito, L.; Keränen, H.; van Vlijmen, H.; Tresadern, G. Predicting Binding Free Energies of PDE2 Inhibitors. The Difficulties of Protein Conformation. *Sci. Rep.* **2018**, *8*, 4883.

(20) Bartolomé-Nebreda, J. M.; Delgado, F.; Martín-Martín, M. L.; Martínez-Viturro, C. M.; Pastor, J.; Tong, H. M.; Iturrino, L.; Macdonald, G. J.; Sanderson, W.; Megens, A.; Langlois, X.; Somers, M.; Vanhoof, G.; Conde-Ceide, S. Discovery of a Potent, Selective, and Orally Active Phosphodiesterase 10A Inhibitor for the Potential Treatment of Schizophrenia. *J. Med. Chem.* **2014**, *57*, 4196–4212.

(21) Boutard, N.; Białas, A.; Sabiniarz, A.; Guzik, P.; Banaszak, K.; Biela, A.; Bień, M.; Buda, A.; Bugaj, B.; Cieluch, E.; Cierpich, A.; Dudek, Ł.; Eggenweiler, H.-M.; Fogt, J.; Gaik, M.; Gondela, A.; Jakubiec, K.; Jurzak, M.; Kitlińska, A.; Kowalczyk, P.; Kujawa, M.; Kwiecińska, K.; Leś, M.; Lindemann, R.; Maciuszek, M.; Mikulski, M.; Niedziejko, P.; Obara, A.; Pawlik, H.; Rzymski, T.; Sieprawska-Lupa, M.; Sowińska, M.; Szeremeta-Spisak, J.; Stachowicz, A.; Tomczyk, M. M.; Wiklik, K.; Włoszczak, Ł.; Ziemiańska, S.; Zarkebski, A.; Brzózka, K.; Nowak, M.; Fabritius, C.-H. Discovery and Structure-Activity Relationships of *N* -Aryl 6-Aminoquinoxalines as Potent PFKFB3 Kinase Inhibitors. *ChemMedChem* **2019**, *14*, 169–181.

(22) Wilson, D. P.; Wan, Z.-K.; Xu, W.-X.; Kirincich, S. J.; Follows, B. C.; Joseph-McCarthy, D.; Foreman, K.; Moretto, A.; Wu, J.; Zhu, M.; Binnun, E.; Zhang, Y.-L.; Tam, M.; Erbe, D. V.; Tobin, J.; Xu, X.; Leung, L.; Shilling, A.; Tam, S. Y.; Mansour, T. S.; Lee, J. Structure-Based Optimization of Protein Tyrosine Phosphatase 1B Inhibitors: From the Active Site to the Second Phosphotyrosine Binding Site. *J. Med. Chem.* **2007**, *50*, 4681–4698.

(23) Chen, Y.-N. P.; LaMarche, M. J.; Chan, H. M.; Fekkes, P.; Garcia-Fortanet, J.; Acker, M. G.; Antonakos, B.; Chen, C. H.-T.; Chen, Z.; Cooke, V. G.; Dobson, J. R.; Deng, Z.; Fei, F.; Firestone, B.; Fodor, M.; Fridrich, C.; Gao, H.; Grunenfelder, D.; Hao, H.-X.; Jacob, J.; Ho, S.; Hsiao, K.; Kang, Z. B.; Karki, R.; Kato, M.; Larrow, J.; La Bonte, L. R.; Lenoir, F.; Liu, G.; Liu, S.; Majumdar, D.; Meyer, M. J.; Palermo, M.; Perez, L.; Pu, M.; Price, E.; Quinn, C.; Shakya, S.; Shultz, M. D.; Slisz, J.; Venkate-

san, K.; Wang, P.; Warmuth, M.; Williams, S.; Yang, G.; Yuan, J.; Zhang, J.-H.; Zhu, P.; Ramsey, T.; Keen, N. J.; Sellers, W. R.; Stams, T.; Fortin, P. D. Allosteric Inhibition of SHP2 Phosphatase Inhibits Cancers Driven by Receptor Tyrosine Kinases. *Nature* **2016**, *535*, 148–152.

(24) Pérez-Benito, L.; Casajuana-Martin, N.; Jiménez-Rosés, M.; van Vlijmen, H.; Tresadern, G. Predicting Activity Cliffs with Free-Energy Perturbation. *J. Chem. Theory Comput.* **2019**, *15*, 1884–1895.

(25) Currie, K. S.; Kropf, J. E.; Lee, T.; Blomgren, P.; Xu, J.; Zhao, Z.; Gallion, S.; Whitney, J. A.; Maclin, D.; Lansdon, E. B.; Maciejewski, P.; Rossi, A. M.; Rong, H.; Macaluso, J.; Barbosa, J.; Di Paolo, J. A.; Mitchell, S. A. Discovery of GS-9973, a Selective and Orally Efficacious Inhibitor of Spleen Tyrosine Kinase. *J. Med. Chem.* **2014**, *57*, 3856–3873.

(26) Baum, B.; Mohamed, M.; Zayed, M.; Gerlach, C.; Heine, A.; Hangauer, D.; Klebe, G. More than a Simple Lipophilic Contact: A Detailed Thermodynamic Analysis of Nonbasic Residues in the S1 Pocket of Thrombin. *J. Mol. Biol.* **2009**, *390*, 56–69.

(27) Buchstaller, H.-P.; Anlauf, U.; Dorsch, D.; Kuhn, D.; Lehmann, M.; Leuthner, B.; Musil, D.; Radtki, D.; Ritzert, C.; Rohdich, F.; Schneider, R.; Esdar, C. Discovery and Optimization of 2-Arylquinazolin-4-Ones into a Potent and Selective Tankyrase Inhibitor Modulating Wnt Pathway Activity. *J. Med. Chem.* **2019**, *62*, 7897–7909.

(28) Liang, J.; Tsui, V.; Van Abbema, A.; Bao, L.; Barrett, K.; Beresini, M.; Berezhkovskiy, L.; Blair, W. S.; Chang, C.; Driscoll, J.; Eigenbrot, C.; Ghilardi, N.; Gibbons, P.; Halladay, J.; Johnson, A.; Kohli, P. B.; Lai, Y.; Liimatta, M.; Mantik, P.; Menghrajani, K.; Murray, J.; Sambrone, A.; Xiao, Y.; Shia, S.; Shin, Y.; Smith, J.; Sohn, S.; Stanley, M.; Ultsch, M.; Zhang, B.; Wu, L. C.; Magnuson, S. Lead Identi-

fication of Novel and Selective TYK2 Inhibitors. *European J. Med. Chem.* **2013**, *67*, 175–187.

(29) Liang, J.; van Abbema, A.; Balazs, M.; Barrett, K.; Berezhkovsky, L.; Blair, W.; Chang, C.; Delarosa, D.; DeVoss, J.; Driscoll, J.; Eigenbrot, C.; Ghilardi, N.; Gibbons, P.; Halladay, J.; Johnson, A.; Kohli, P. B.; Lai, Y.; Liu, Y.; Lyssikatos, J.; Mantik, P.; Menghrajani, K.; Murray, J.; Peng, I.; Sambrone, A.; Shia, S.; Shin, Y.; Smith, J.; Sohn, S.; Tsui, V.; Ultsch, M.; Wu, L. C.; Xiao, Y.; Yang, W.; Young, J.; Zhang, B.; Zhu, B.-y.; Magnuson, S. Lead Optimization of a 4-Aminopyridine Benzamide Scaffold To Identify Potent, Selective, and Orally Bioavailable TYK2 Inhibitors. *J. Med. Chem.* **2013**, *56*, 4521–4536.

(30) Warren, G. L.; Do, T. D.; Kelley, B. P.; Nicholls, A.; Warren, S. D. Essential Considerations for Using Protein–Ligand Structures in Drug Discovery. *Drug Discovery Today* **2012**, *17*, 1270–1281.

(31) Gapsys, V.; Pérez-Benito, L.; Aldeghi, M.; Seeliger, D.; van Vlijmen, H.; Tresadern, G.; de Groot, B. L. Large Scale Relative Protein Ligand Binding Affinities Using Non-Equilibrium Alchemy. *Chem. Sci.* **2020**, *11*, 1140–1152.

(32) Hahn, D. F.; Wagner, J. R. Protein-Ligand Benchmark Dataset for Free Energy Calculations. 2022; `https://zenodo.org/record/6600875`, (accessed 2024-04-23).

(33) Hahn, A. M.; Then, H. Measuring the convergence of Monte Carlo free-energy calculations. *Phys. Rev. E* **2010**, *81*, 041117.
